# Vitrectomy, subretinal Tissue plasminogen activator and Intravitreal Gas for submacular haemorrhage secondary to Exudative Age-Related macular degeneration (TIGER): update to study protocol and addition of a statistical analysis plan and health economic analysis plan for a randomised controlled surgical trial

**DOI:** 10.1186/s13063-025-08727-8

**Published:** 2025-04-14

**Authors:** Chan Ning Lee, Riti Desai, Lisa Ramazzotto, Hatem Wafa, Yanzhong Wang, Catey Bunce, Kodchawan Doungsong, Victory Ezeofor, Rhiannon T. Edwards, Noemi Lois, David H. Steel, Tunde Peto, Jost Hillenkamp, Jan C. van Meurs, Barnaby C. Reeves, Timothy L. Jackson

**Affiliations:** 1 Department of Ophthalmology, King’s College Hospital, London, UK; 2 Faculty of Life Sciences and Medicine, King’s College London, London, UK; 3 Population Health Sciences, Faculty and Life Science and Medicine, King’s College London, London, UK; 4 The London School of Hygiene and Tropical Medicine, London, UK; 5 Centre for Health Economics and Medicines Evaluation, Bangor University, Bangor, Wales; 6 Wellcome-Wolfson Institute for Experimental Medicine, Queen’s University, Belfast, Northern Ireland; 7 Bioscience Institute, Newcastle University, Newcastle, UK; 8 Network of Ophthalmic Reading Centres UK, Queen’s University, Belfast, Northern Ireland; 9 Eye Clinic, University of Wurzburg, Wurzburg, Germany; 10 Rotterdam Eye Hospital and Erasmus Medical Centre, Rotterdam, the Netherlands; 11 University of Bristol, Bristol, UK

**Keywords:** Submacular haemorrhage, Vitrectomy, Surgery, Randomised controlled trial, Statistical analysis, Health economic analysis

## Abstract

**Background:**

The vitrectomy, subretinal Tissue plasminogen activator and Intravitreal Gas for submacular haemorrhage secondary to Exudative Age-Related macular degeneration (TIGER) trial is a pan-European, two-group, non-commercial, active-control, observer-masked, superiority, randomised controlled surgical clinical trial of an investigational medicinal product.

**Methods:**

The original protocol for this trial was published on 31 January 2022 (https://trialsjournal.biomedcentral.com/articles/10.1186/s13063-021-05966-3). This update reports on key changes in the study protocol in version 2.0 which was approved for trial sites from 18 January 2022, and the current version 3.0 which was approved for trial sites from 25 April 2023, and includes current versions of the statistical analysis plan and health economics analysis plan. In summary, there have been changes to three eligibility criteria: removing the word “Actilyse” from exclusion criterion 2, updating exclusion criterion 5 to state abstinence from heterosexual intercourse or the use of highly effective methods of birth control is mandatory for up to 12 weeks after last aflibercept exposure on trial, and clarifying exclusion criterion 6 relating to international normalised ratio (INR) is only applicable to participants receiving warfarin. Changes to secondary outcomes include Radner Reading speed being limited to the study eye only, and moving EQ-5D-5L from a secondary reported efficacy outcome to a component of health economic analysis reporting only. Actilyse Cathflo was added as an additional permitted investigational medicinal product as this is already used in practice in the UK and is molecularly identical to Actilyse 10 mg. Instructions were added to account for participants who had already been exposed to aflibercept or a similar anti-vascular endothelial growth factor (anti-VEGF) within 21 days (the minimum window between anti-VEGF treatments permitted on trial) prior to study enrolment, storage of tissue plasminogen activator in theatre and operating room environments, and the recording of additional, as-needed aflibercept treatments in-between study visits at the discretion of the study investigator. Finally, sections and subsections have been added to detail the imaging analysis plan, patient public involvement plan, INR testing, and recruitment and informed consent components of the trial. The primary analysis of the trial as stated in the statistical analysis plan is the difference between groups in the proportion of participants gaining ≥ 10 ETDRS letters in their study eye at the month 12 visit, whilst the primary health economic analysis of the trial is the difference in quality-adjusted life years between groups at 12 months.

**Trial registration:**

ClinicalTrials.gov identifier: NCT04663750; EudraCT: 2020–004917-10.

**Supplementary Information:**

The online version contains supplementary material available at 10.1186/s13063-025-08727-8.

## Update

### Introduction

The TIGER trial is a pan-European randomised controlled surgical trial (RCT) aiming to test the hypothesis that vitrectomy, subretinal tissue plasminogen activator (TPA), intravitreal sulfahexafluoride (SF_6_) gas tamponade, and aflibercept are superior to aflibercept monotherapy, with respect to Early Treatment of Diabetic Retinopathy Study (ETDRS) best-corrected visual acuity (BCVA) as the primary outcome, in the management of submacular haemorrhage (SMH) secondary to exudative age-related macular degeneration (AMD) at 12 months. TPA (alteplase) is used off-label and therefore, from a regulatory perspective, TIGER is a Clinical Trial of an Investigational Medicinal Product (CTIMP).

The original protocol (version 1.3) was published on 31 January 2022 [1]. During the lifetime of the trial, a number of amendments have been made to the trial protocol including amendments to three eligibility criteria, amendments to the testing and analysis of two secondary outcome measures, the inclusion of a specific brand of licensed alteplase (Actilyse Cathflo 2 mg) as an alternative to Actilyse 10 mg, 20 mg, or 50 mg vials, and amendments and additions to clarify aspects of trial methodology and bring the protocol in-line with regulatory requirements. These have been reflected in protocol versions 2.0 (approved 18 January 2022) and 3.0 (approved 25 April 2023). The key amendments have been described below and are also included in supplementary materials as protocol version 2.0 with an accompanying list of changes (Appendices 1 and 2) and protocol version 3.0 with an accompanying list of changes (Appendices 3 and 4).

In addition, a full statistical analysis plan (SAP, Appendix 5) and health economic analysis plan (HEAP, Appendix 6), expanding on the analytical approaches outlined in the relevant protocol subsections, have been finalised and included as supplementary documents.

### Changes in eligibility criteria (Protocol version 3.0, 25th April 2023)

In response to queries from sites and European regulators, clarifications and changes were made to exclusion criteria 2, 5, and 6, which can be found in their original version in the published trial protocol [1] and in their updated version in the attached protocol version 3.0 (Appendix 3, pp 23-25).

The study team has considered, in light of the ongoing global shortage of Actilyse which began in September 2022 and is projected to last until the end of 2023 [2], whether sites might only have access to non-licensed brands (i.e. non-Actilyse brands) of alteplase. Criterion 2 was therefore changed by removing the word “(Actilyse)” so as not to restrict hypersensitivity to a single brand of alteplase as an exclusion criterion. At present, however, sites have reported that supplies of Actilyse and Actilyse Cathflo are sufficient to allow ongoing use of Actilyse as the intended brand of IMP to be used for the TIGER trial, and no non-licensed brand of alteplase is intended to be used on the TIGER trial until or unless this situation changes.

Exclusion criterion 5 was amended to require “abstinence from heterosexual intercourse or to use highly effective methods of birth control for the duration up to 12 weeks after administration of IMP *or the last administration of aflibercept on the trial*.” This was done to bring the criterion in-alignment with published product characteristic information for aflibercept, which states women “have to use effective contraception during treatment and for at least 3 months after the last intravitreal injection of aflibercept” [3].

Exclusion criterion 6 was intended to only apply to participants receiving warfarin therapy wherein international normalised ratio (INR) testing would be relevant. This has been clarified with the addition of a clarification asterisk (*) stating it only applies to “*participants receiving warfarin*” and the addition of section 6.2.1 to clarify (see below).

### Addition of sections (Protocol version 2.0, 18th January 2022 and Protocol version 3.0, 25th April 2023)

The following sections were added in Protocol version 2.0 (18th January 2022):

- Section 11 “Image Analysis” was added by the trial reading centre (NetwORC UK) to clarify their trial-specific responsibilities, protocol for image submission, and reading centre outputs (Appendix 1, p44).
- Section 13 “Patient Public Involvement” was added by the trial team to describe trial-specific patient-public involvement (PPI) activities that had taken place during the trial conception stage, as well as planned further PPI activities (Appendix 1, pp46-47).

The following sections were added in Protocol version 3.0 (25th April 2023):

- Subsection 6.2.1 “INR testing” to detail which participants would need to undergo INR testing in respect to exclusion criterion 6 and in which circumstances a recent INR result would be acceptable in lieu of testing at screening—specifically, an INR result within 4 weeks of trial screening with no change in warfarin dosing in the preceding 8 weeks (Appendix 3, p25).
- Subsection 6.5 “Recruitment and Informed Consent” to detail the process of trial recruitment and consent and the responsibilities and duties of investigators in this process. This subsection was added in response to regulatory feedback from European sites to bring the protocol in line with European Union (EU) requirements (Appendix 3, pp25-26).

### Addition of other brand of medication (Protocol version 2.0, 18th January 2022)

TPA (alteplase, Actilyse) is a 70-kDA glycoprotein enzyme that activates plasminogen to plasmin, which in turn breaks down fibrin clots. It is commercially available in 10 mg, 20 mg, and 50 mg vial forms and licensed in the United Kingdom (UK) and EU to treat myocardial infarction, acute ischaemic stroke, and pulmonary embolism. TPA is additionally produced under the brand name Actilyse Cathflo, which is commercially available as a 2 mg vial and is licensed to keep intravenous access devices patent. Though subject to different licensing and storage criteria, and containing 2.2 mg of dry weight active substance, Actilyse Cathflo is molecularly identical to 10 mg alteplase TPA and, as it is similarly used off-license for the treatment of SMH and some sites in the UK reported only having access to this form of TPA for subretinal injection, section 5.1 in the protocol was amended to allow this brand of TPA to be used for the purposes of the TIGER trial (Appendix 1, p15).

### Baseline treatments for participants in control group (Protocol version 2.0, 18th January 2022)

To remain within the marketing authorisation of aflibercept, a minimum window of 21 days between trial treatments (accounting for visit windows of 28 ± 7 days for the first three visits) is required; however, the original protocol did not have specific guidance in the event of participants who develop SMH in the study eye following administration of standard-of-care anti-vascular endothelial growth factor (VEGF) within 21 days of trial screening.

Additions have been added to section 5.4.2 “Aflibercept (Eylea)” to account for this (Appendix 1, pp18-19). For participants randomised to surgery, it has been clarified that as any remaining intravitreal aflibercept will be removed with vitrectomy, aflibercept should be administered at the end of surgery. For participants randomised to aflibercept monotherapy (control arm), it has been clarified that baseline trial aflibercept treatment should be delayed by 7 days if anti-VEGF was given to the study eye 2 weeks prior to screening or omitted if anti-VEGF was given to the study eye 1 week or less prior to screening.

### Storage of TPA in theatre (Protocol version 2.0, 18th January 2022)

To accommodate site-specific practices relating to TPA storage and reconstitution (for example, storage in a refrigerator in the operating theatre, or reconstitution in a clean room), text was added to section 5.7 “Drug Accountability and Disposal” (Appendix 1, p22) and 5.8 “Storage of IMP” (Appendix 1, pp22-23) to allow for TPA storage in theatre, or reconstitution in clean areas then delivered to operating theatres, per site’s usual practice.

### Secondary outcome measure(s) (Protocol version 2.0, 18th January 2022)

The following secondary outcome measures were amended for clarity:

- Radner Reading Speed: Study Eye Only

In the original protocol, Radner reading acuity was intended to be measured in study eye only at baseline and month 6, and both eyes at month 12 [1]. This was amended in protocol version 2.0 (18th January 2022) to be measured in the study eye only at all three time points, as the addition of fellow eye reading speed at month 12 was not determined to be of additional value (Appendix 1, p29 and p55).

- Moving of EQ-5D-5L from secondary outcome measure into health economics analysis

In the original protocol, the EQ-5D-5L questionnaire was listed to be reported independently as a secondary efficacy outcome [1], but this has been moved to be part of the health economics analysis in subsequent protocol versions (Appendix 1, pp45-46). Although this questionnaire contains some vision items (as part of the 5L vision bolt-on), the largely non-ophthalmic quality-of-life nature of the questions were felt to be more relevant to the reporting of health economics than clinical efficacy, especially given that the secondary efficacy outcomes already incorporate a specific visual function questionnaire (Visual Function Questionnaire-25, VFQ-25).

### Additional injections of aflibercept (study eye) (Protocol version 3.0, 25th April 2023)

Based on feedback from sites, we recognise that participants might occasionally need injections of aflibercept in the study eye more frequently than stipulated in the trial protocol (monthly for 3 doses, then 2-monthly up to month 12) to adequately control exudative AMD activity and preserve visual function. Wording has been added to section 5.4.2 “Aflibercept (Eylea)” to address this possibility, requesting sites record any additional non-protocol mandated injections as deviations and add separate entries in the concomitant medications log on source data worksheets and the electronic data capture system (Appendix 3, p18).

### Minor wording changes

A full list of wording changes, amendments, and corrections and clarifications in protocols version 2.0 and 3.0 have been included in supplementary appendices 2 and 4 respectively.

## Current status of the TIGER trial

The TIGER trial is still actively recruiting, having now activated sites in Germany and setting up sites in the Netherlands, the Republic of Ireland, and Poland. The trial has recruited 109 participants of its target 210 with a current predicted end of recruitment of August 2025.

## Supplementary Information

Additional file 1: Appendix 1. TIGER Master Protocol version 2.0.

Additional file 2: Appendix 2. Summary of Changes in TIGER Master Protocol version 2.0.

Additional file 3: Appendix 3. TIGER Master Protocol version 3.0.

Additional file 4: Appendix 4. Summary of Changes in TIGER Master Protocol version 3.0.

Additional file 5: Appendix 5. TIGER statistical analysis plan.

Additional file 6: Appendix 6. TIGER health economic analysis plan.

Additional file 7: Appendix 7. TIGER SAP Checklist.

## Data Availability

The lead sponsor (King’s College London) and clinical co-sponsor (King’s College Hospital) own and control the trial data. It is hoped to make data open access on completion of the trial.

## Abbreviations

AMD: Age-related macular degeneration
BCVA: Best-corrected visual acuity
CTIMP: Clinical Trial of an Investigational Medicinal Product
EQ-5D-5L: EuroQol-5D questionnaire with 5-item vision bolt-on
ETDRS: Early Treatment of Diabetic Retinopathy Study
EU: European Union
EURETINA: European Society of Retinal Specialists
HEAP: Health economic analysis plan
HCRW: Health Care Research Wales
HRA: Health Research Authority
ICF: Informed consent form
ICMJE: International Committee of Medical Journal Editors
IMP: Investigational medicinal product
INR: International normalised ratio
kDa: Kilodalton
NetwORC UK: The Network of Ophthalmic Reading Centres United Kingdom
NHS: National Health Service
PPI: Patient public involvement
RCT: Randomised controlled trial
REC: Research Ethics Committee
SAP: Statistical analysis plan
SF_6_: Sulfahexafluoride gas
SMH: Submacular haemorrhage
TIGER: Vitrectomy, subretinal Tissue plasminogen activator and Intravitreal Gas for submacular haemorrhage secondary to Exudative Age-Related macular degeneration
TPA: Tissue plasminogen activator
UK: United Kingdom
VEGF: Vascular endothelial growth factor
VFQ-25: 25-Item Visual Function Questionnaire
