# Supplementary material for: Vitrectomy, subretinal Tissue plasminogen activator and Intravitreal Gas for submacular haemorrhage secondary to Exudative Age-Related macular degeneration (TIGER): update to study protocol and addition of a statistical analysis plan and health economic analysis plan for a randomised controlled surgical trial
Source: Trials. 2025 Apr 14;26:131. doi: 10.1186/s13063-025-08727-8 (PMC11995560; doi:10.1186/s13063-025-08727-8)
Supplement: Supplementary file 1 — Additional file 1: Appendix 1. TIGER Master Protocol version 2.0. [file 13063_2025_8727_MOESM1_ESM.pdf]

TIGER

KING'S HEALTH PARTNERS

An Academic Health Sciences Centre for London    Pioneering better health for all

# TIGER Study Protocol

## Vitrectomy, subretinal Tissue plasminogen activator and Intravitreal Gas for submacular haemorrhage secondary to Exudative age-Related macular degeneration (TIGER): a phase 3, pan-European, two-group, active-control, observer-masked, superiority, randomised controlled surgical trial.

**Chief Investigator**
Prof Tim Jackson
King's College London
Department of Ophthalmology
King's College Hospital NHS
Foundation Trust
Denmark Hill
London
Tel : +44 (0) 20 3299 1297
Email: t.jackson1@nhs.net

**Co-Sponsors**
King's College London &
King's College Hospital NHS
Foundation Trust
Amy Holton
King's Health Partners Clinical Trials Office
Floor 16, Tower Wing,
Guy's Hospital,
London
Tel: 07703469925
Email: amy.holton@kcl.ac.uk

**Reading Centre Lead**
Prof Tunde Peto
Network of Ophthalmic Reading Centres UK
Central Administrative Research Facility
Queen's University Belfast,
Northern Ireland.
Tel : +44 (0) 28 9097 1659
Email: T.Peto@qub.ac.uk

**Clinical Co-Investigators**
Prof David Steel
The University of Newcastle upon Tyne
Tel: +44 (0) 191 5699065
Email: david.steel@newcastle.ac.uk

Prof Noemi Louis
Queen's University Belfast
Tel: +44 (0)28 9097 6462
Email: n.lois@qub.ac.uk

**Trial Methodologist**
Prof Barnaby Reeves
University of Bristol
Tel: +44 (0) 117 34 23143
Email: Barney.Reeves@bristol.ac.uk

**Lead Statistician**
Dr Yanzhong Wang
King's College London
Tel: +44 (0) 20 7848 8223
Email: yanzhong.wang@kcl.ac.uk

**Health Economist**
Prof Rhiannon Tudor Edwards
Co- Director Centre for Health Economics
and Medicines Evaluation
Bangor University,
Bangor, Gwynedd,
Tel: +44 (0) 1248 383712
Email: r.t.edwards@bangor.ac.uk

FIGHT FOR SIGHT    EURETINA    networc uk    NHS King's College Hospital NHS Foundation Trust    KING'S College LONDON

# 1. Study Synopsis

| | |
|---|---|
| Title of clinical trial | Vitrectomy, subretinal Tissue plasminogen activator and Intravitreal Gas for submacular haemorrhage secondary to Exudative age-Related macular degeneration (TIGER): a phase 3, pan-European, two-group, observer-masked, superiority, randomised controlled surgical trial. |
| Protocol short title/acronym | Vitrectomy and subretinal TPA for submacular haemorrhage secondary to wet AMD (TIGER). |
| Sponsor | King's College London (lead sponsor) and King's College Hospital NHS Foundation Trust (clinical co-sponsor) |
| Chief Investigator | Prof Tim Jackson |
| EudraCT number | 2020-004917-10 |
| IRAS ID | 276366 |
| Research Ethics Committee number | 20/EE/0293 |
| ClinicalTrial.gov identifier | NCT04663750 |
| Medical condition or disease under investigation | Submacular haemorrhage (SMH) secondary to exudative age-related macular degeneration (AMD). |
| Purpose of clinical trial | To assess the safety and efficacy of vitrectomy, subretinal tissue plasminogen activator (TPA), gas tamponade and intravitreal anti-vascular endothelial growth factor (anti-VEGF) as a treatment for SMH secondary to exudative AMD, versus standard of care with anti-VEGF monotherapy |
| Trial design | A phase 3, pan-European, two-group, active control, observer-masked, superiority, randomised controlled surgical trial. |
| Primary endpoint | Gain ≥10 Early Treat of Diabetic Retinopathy Study (ETDRS) letters of best-corrected visual acuity (BCVA) in the study eye at the month 12 visit. |
| Secondary outcomes (at 6 and 12 months unless noted otherwise) | Gain of ≥10 ETDRS letters (month 6); mean ETDRS BCVA; Radner reading vision; National Eye Institute 25 item Visual Function Questionnaire composite score; scotoma size (Humphrey Field Analyser 10-2); presence/absence of subfoveal fibrosis and/or atrophy and area of fibrosis/atrophy using multimodal reading centre image analysis (month 12). |
| Sample size | 210 randomised 1:1 to surgery or standard of care. |
| Summary of key eligibility criteria (full list in Section 6) | **Inclusion criteria:**<br><br>***General***<br><br>1. Males or females aged at least 50 years<br><br>***Study eye***<br><br>2. SMH, comprising sub-neuroretinal haemorrhage with or without sub-RPE haemorrhage, that occurs secondary to treatment naïve, or previously treated |

| | |
|---|---|
| | exudative AMD, including choroidal neovascularisation (CNV), idiopathic polypoidal choroidal vasculopathy (IPCV) and retinal angiomatous proliferation (RAP)<br>3. SMH involving the foveal centre that measures at least 1 disc diameter in greatest linear dimension<br>4. Sub-neuroretinal haemorrhage at least 125 microns thick, measured at the foveal centre using spectral domain optical coherence tomography (SD-OCT)<br>5. BCVA between counting fingers and an Early Treatment of Diabetic Retinopathy Study (ETDRS) letter score of 70, inclusive.<br><br>**Exclusion criteria:**<br><br>***General***<br>1. Serious allergy to fluorescein or indocyanine green (ICG).<br><br>2. Hypersensitivity to alteplase, gentamicin, arginine, phosphoric acid, polysorbate 80 or aflibercept (Eylea).<br><br>3. Stroke, transient ischaemic attack or myocardial infarction within 6 months.<br><br>4. Participation in another interventional study within 12 weeks of enrolment or planned to occur during this study.<br><br>5. Women who are breast feeding, pregnant, or planning to become pregnant during the clinical trial. Any sexually active women of childbearing potential must agree continued abstinence from heterosexual intercourse or to use highly effective methods of birth control for the duration up to 12 weeks post IMP administration. Men must also agree to use a condom if their partner is of child bearing potential, even if they have had a successful vasectomy. Females of childbearing potential are females who have experienced menarche and are not surgically sterilised (e.g. hysterectomy or bilateral salpingectomy) or post-menopausal (defined as at least 1 year since last regular menstrual period). Highly effective methods of birth control are those with a failure rate of < 1% per year when employed consistently and correctly, eg. combined (oestrogen and progestogen containing) hormonal contraception associated with inhibition of ovulation via oral, intravaginal, and transdermal routes; progestogen-only hormonal contraception associated with inhibition of ovulation via oral, injectable, implantable, intrauterine device (IUD), or intrauterine hormone-releasing system ( IUS); or vasectomised partner.<br><br>6. International Normalised Ratio (INR) greater than 3.5, unless it is anticipated that the INR can be brought below this level prior to |

| | |
|---|---|
| | vitrectomy, balancing the systemic risks with those of intraocular haemorrhage.<br><br>7. Unwilling, unable, or unlikely to return for scheduled follow-up for the duration of the trial.<br><br>8. Any other condition which, in the opinion of the investigator, would prevent the participant from granting informed consent or complying with the protocol, such as dementia, mental illness, or serious systemic medical disease<br><br>***Study eye***<br><br>9. SMH that is known or estimated to have been present for longer than 15 days, as evidenced by history, pre-trial clinical documentation, or fundus appearance.<br><br>10. SMH due to eye disease other than exudative AMD.<br><br>11. Current active proliferative diabetic retinopathy.<br><br>12. Current intraocular inflammation.<br><br>13. Current ocular or periocular infection other than blepharitis.<br><br>14. Current or known former high myopia (>6 dioptres).<br><br>15. Aphakia.<br><br>16. Other current or pre-existing ocular conditions that, in the opinion of the Investigator, will preclude any improvement in BCVA following resolution of SMH, such as severe central macular atrophy or fibrosis, dense amblyopia, macular hole involving the fovea, or very poor BCVA prior to presentation with SMH (counting fingers or worse).<br><br>17. Inadequate pupillary dilation or significant media opacities, which will prevent adequate clinical evaluation of the posterior segment or fundus imaging.<br><br>18. Intraocular surgery within 12 weeks of enrolment except for uncomplicated cataract surgery, which is permitted within 8 weeks of enrolment. |
| Intervention | Pars plana vitrectomy, subretinal injection of recombinant TPA (Alteplase) up to a maximum of 25 micrograms in 0.25 mls, intravitreal 20% |

| | |
|---|---|
| | sulfahexafluoride ($SF_6$) gas tamponade, and intravitreal 2 mg aflibercept (Eylea, Bayer).<br>After surgery participants will be advised to sit up and lean forward during the day for 5 days, and sleep with the operative cheek dependent for 10 days.<br>Intravitreal 2 mg aflibercept will be injected monthly for two further doses, then 2-monthly until month 12. |
| Active comparator | Intravitreal 2 mg aflibercept monthly for three doses, then 2-monthly until month 12. |
| Version and date of protocol amendments | Version 2.0 – 6th December 2021 |

# 2. Table of Contents

**TIGER STUDY PROTOCOL** .......... **1**

**1. STUDY SYNOPSIS** .......... **2**

**2. TABLE OF CONTENTS** .......... **6**

**3. BACKGROUND & RATIONALE** .......... **9**

**4. TRIAL OBJECTIVES AND DESIGN** .......... **11**

4.1. TRIAL OBJECTIVE .......... 11
4.2 TRIAL DESIGN AND FLOW DIAGRAM .......... 12
4.3 TRIAL ORGANISATION .......... 13

**5. TRIAL MEDICATION AND SURGERY** .......... **14**

5.1 INVESTIGATIONAL MEDICINAL PRODUCT (IMP): TISSUE PLASMINOGEN ACTIVATOR (TPA, ALTEPLASE) .......... 14
5.2 TPA (ALTEPLASE) DOSE, INJECTION TECHNIQUE AND SURGERY .......... 14
*5.2.1 TPA (Alteplase) Dose* .......... *15*
*5.2.2 TPA (Alteplase) Injection and Surgical Technique, Required Surgeon Experience, and Timing of Surgery* .......... *15*
5.3 POST-OPERATIVE HEAD POSITIONING (POSTURING) .......... 17
5.4 CONCOMITANT TREATMENTS .......... 18
*5.4.1. Post-operative eye drops* .......... *18*
*5.4.2 Aflibercept (Eylea)* .......... *18*
5.5 OTHER PERMITTED TREATMENTS FOR CATARACT, AGE-RELATED MACULAR DEGENERATION AND IDIOPATHIC POLYPOIDAL CHOROIDAL VASCULOPATHY .......... 20
*5.5.1 Photodynamic Therapy (PDT) for Idiopathic Polypoidal Choroidal Vasculopathy (IPCV)* .......... *20*
*5.5.2 Reoperation for Recurrent Submacular Haemorrhage* .......... *20*
*5.5.3 Cataract surgery (Including Phakovitrectomy)* .......... *20*
5.6 IMP RISKS .......... 20
5.7 DRUG ACCOUNTABILITY AND DISPOSAL .......... 22
5.8 STORAGE OF IMP .......... 22

**6. SELECTION AND WITHDRAWAL OF SUBJECTS** .......... **23**

6.1 INCLUSION CRITERIA .......... 23
6.2 EXCLUSION CRITERIA .......... 24
6.3 SELECTION OF PARTICIPANTS .......... 25
6.4 EMERGENCY CODE BREAK .......... 25
6.5 WITHDRAWAL OF SUBJECTS .......... 25
6.6 RECRUITMENT TIMELINES AND TRIAL DURATION .......... 26

**7. SCHEDULE OF ACTIVITIES** .......... **28**

7.1 SCHEDULE OF ACTIVITIES BY VISIT TABLE .......... 28
7.2 PROCEDURES BY VISIT .......... 30
*7.2.1 Screening and Baseline Treatment* .......... *30*
*7.2.2 Day 1 and 7 (Arm A and study eye only)* .......... *31*
*7.2.3 Month 1 and 2* .......... *31*

*7.2.4 Month 4* ... *32*
*7.2.5 Month 6* ... *33*
*7.2.6 Month 8* ... *33*
*7.2.7 Month 10* ... *34*
*7.2.8 Month 12 (final visit)* ... *34*
7.3 READING CENTRE IMAGE ANALYSIS ... 35
*7.3.1 Delayed angiography* ... *35*
**8. ASSESSMENT OF EFFICACY ... 35**
8.1 PRIMARY EFFICACY OUTCOME ... 35
8.2 SECONDARY EFFICACY OUTCOMES ... 36
**9. ASSESSMENT OF SAFETY ... 36**
9.1 SAFETY PARAMETERS ... 36
9.2 DEFINITIONS ... 37
*9.2.1 Adverse Event (AE)* ... *37*
*9.2.2 Adverse Reaction (AR)* ... *37*
*9.2.3 Unexpected Adverse Reaction (UAR)* ... *37*
*9.2.4 Serious Adverse Event (SAE), Serious Adverse Reaction (SAR) or Suspected Unexpected Serious Adverse Reaction (SUSAR)* ... *37*
*9.2.5 Important Medical Events (IME) & Pregnancy* ... *37*
*9.2.6 Reporting Responsibilities and Timelines* ... *37*
*9.2.7 Adverse events that do not require reporting* ... *38*
9.3 TREATMENT STOPPING RULES AND PREMATURE TRIAL DISCONTINUATION ... 39
*9.3.1 TPA (Alteplase)* ... *39*
*9.3.2 Stopping Aflibercept* ... *39*
*9.3.3 Trial Discontinuation* ... *39*
**10. STATISTICS ... 39**
10.1 CLINICALLY MEANINGFUL DIFFERENCE, PUBLIC PATIENT INVOLVEMENT (PPI) AND SAMPLE SIZE ... 39
10.2 RANDOMISATION AND STRATIFICATION ... 40
10.3 ANALYSIS ... 40
*10.3.1 Primary Analysis* ... *40*
*10.3.2 Safety* ... *41*
*10.3.3 Missing Data* ... *41*
*10.3.4 Subgroup Analyses* ... *41*
*10.3.5 Interim Analysis* ... *42*
*10.3.6 Pharmacokinetics and pharmacodynamics* ... *43*
*10.3.7 Measures to Minimise Bias* ... *44*
**11. IMAGE ANALYSIS ... 45**
**12. HEALTH ECONOMICS ANALYSIS ... 47**
*12.1 Economic research question* ... *45*
*12.2 Health economic evaluation* ... *45*
**13. PATIENT AND PUBLIC INVOLVEMENT ... 45**
*13.1 Patient Involvement Group* ... *455*
*13.2 Study Patient Feedback Group* ... *455*

*13.3 Macular Society Involvement* ... *46*
*13.4 Patient Involvement Adviser* ... *456*
*13.5 Study Result Dissemination* ... *456*
**14. TRIAL STEERING COMMITTEE ... 46**
**15. DATA MONITORING AND ETHICS COMMITTEE ... 47**
**16. ACCESS TO SOURCE DATA AND DOCUMENTS ... 48**
**17. ETHICS & REGULATORY APPROVALS ... 48**
**18. QUALITY ASSURANCE ... 49**
**19. DATA HANDLING ... 49**
**20. DATA MANAGEMENT ... 49**
**21.PUBLICATION POLICY ... 49**
**22. INSURANCE / INDEMNITY ... 50**
**23. FINANCIAL ASPECTS ... 50**
**24. REFERENCES ... 51**
**25. SIGNATURES ... 53**
**26. APPENDIX A: TESTING OF DISTANCE VISUAL ACUITY AND RADNER READING SPEED ... 55**
26.1 CLINIC ETDRS DISTANCE VISUAL ACUITY ... 55
26.2 FULL REFRACTED ETDRS DISTANCE VISUAL ACUITY ... 56
26.3 RADNER READING CHART ... 65
**27. APPENDIX B: AREDS LENS OPACITY GRADING ... 67**
**28. APPENDIX C: VISUAL FIELD TESTING ... 70**
**29. APPENDIX D: LIST OF ABBREVIATIONS ... 71**

## 3. Background & Rationale

In high-income nations, exudative and non-exudative AMD together cause more blindness than all other eye diseases combined.[1] Exudative (wet) AMD can sometimes be associated with a large submacular haemorrhage (SMH).

Whilst exudative AMD is a very common disease, an associated large SMH is not. A population-based study in two UK centres found that SMHs larger than 1 disc diameter across occur in 24 people per million per year, with a Scottish Ophthalmic Surveillance Unit (SOSU) study reporting that SMHs larger than 2 disc diameters occur in only 5.4 people per million per year.[2,3] These data suggest a prevalence rate that would meet the European Commission's definition of a rare disease.[4,5]

Untreated, SMH typically leads to permanent and severe loss of vision, ranging from 6/30 to light perception.[5] The control group of the submacular surgery trial reported that only 11% of eyes had a final best-corrected visual acuity (BCVA) better than 6/60.[6] Preclinical studies suggest loss of vision occurs because subfoveal blood leads to rapid photoreceptor damage due to iron-catalyzed free radicals via the Fenton reaction, mechanical fibrin contraction, and reduced oxygen and nutrient flux.[7] The end result is usually a large fibrotic macular scar (38%), atrophy (25%), or retinal pigment epithelium (RPE) tear (22%), with a resulting central scotoma.[5]

There are no large, published randomised controlled trials (RCTs) evaluating treatments of SMH. The registration RCTs testing anti-vascular endothelial growth factor (VEGF) drugs for exudative AMD specifically excluded this group.

There is no widely accepted treatment approach. The SOSU study reported that 21% of patients with SMH were managed by observation, 21% by anti-VEGF therapy alone, and 58% by combined vitrectomy, tissue plasminogen activator (TPA), anti-VEGF therapy, and intravitreal gas.[2]

In 2016 we published a systematic review and quantitative synthesis of the SMH literature, containing 159 references identified using PubMed Medline, EMBASE and Cochrane Library Databases.[7] This revealed no RCTs. The literature confirmed the poor natural history of SMH and identified three main treatment strategies:

1. Intravitreal gas (to mechanically displace the haemorrhage) and tissue plasminogen activator (TPA, to dissolve the clot), with or without anti-VEGF therapy (to control the underlying disease)
2. Vitrectomy, subretinal TPA and gas, with or without anti-VEGF therapy
3. Anti-VEGF monotherapy

Using mostly patient-level data or analyses weighted for study size, we found that the best BCVA outcome was associated with treatment by intravitreal gas, TPA and anti-VEGF therapy (final mean Snellen equivalent 20/66; n=58). The next best was anti-

VEGF monotherapy (final BCVA 20/126; n=109). The greatest BCVA gain, however, occurred with vitrectomy, subretinal TPA, gas and anti-VEGF (improving from 20/1002 to 20/171; n=59). There were, however, important differences in the baseline characteristics, most notably the surgical groups had worse presenting BCVA.

Since our review there has been one published RCT. This exploratory study (N=24) compared intravitreal TPA, gas and bevacizumab versus vitrectomy, subretinal TPA, gas and aflibercept. It lacked power to detect any statistically significant difference and concluded that larger studies are needed to determine the best treatment approach.[8]

We have undertaken a multicentre, factorial, pilot RCT investigating intravitreal gas, TPA and ranibizumab (TAPAS; ClinicalTrials.gov Identifier: NCT01835067). Recruitment to TAPAS (n=55) is complete and patients are in follow up.

A French RCT (n=90) compares vitrectomy, air, TPA and anti-VEGF therapy versus intravitreal gas, TPA and anti-VEGF therapy (STAR; ClinicalTrials.gov Identifier: NCT02557451). Results of the STAR study are awaited.

Due to differences in case mix none of the different case series can be directly compared, and likewise none of the emerging RCTs are large enough to definitively determine the best management option.

An appropriately powered RCT is therefore needed to determine how best to treat SMH secondary to exudative AMD. Methodologically, we favour a study design that compares what we believe to be the best new treatment with standard care.

Based on our synthesis of the literature, vitrectomy, subretinal TPA, gas and anti-VEGF therapy provide the greatest gain in mean BCVA, and can accommodate the greatest range of presenting BCVA and SMH size. This therefore constitutes the intervention arm for TIGER.

It could be argued that standard care is observation, as patients with SMH were usually excluded from the anti-VEGF registration trials, and there is no licensed treatment for SMH *per se*. However, anti-VEGF monotherapy is licensed for exudative AMD and so withholding it might be considered unethical; more so as our synthesis of the literature suggests anti-VEGF monotherapy produces outcomes that are far better than observation. Therefore, our active control group is anti-VEGF monotherapy.

We have selected aflibercept as it is the most commonly used licensed anti-VEGF therapy, with favourable safety and efficacy as a treatment of exudative AMD.[9]

In the absence of RCT evidence comparing surgery and anti-VEGF monotherapy we remain in equipoise. Case series suggest surgery produces the greatest vision gain, but visual results following anti-VEGF monotherapy were also positive, and without the risks, inconvenience, discomfort, recovery, post-operative posturing and expense of surgery.

We aim to test the hypothesis that vitrectomy, subretinal TPA, intravitreal sulfahexafluoride ($SF_6$) gas tamponade and aflibercept are superior to aflibercept monotherapy, with respect to BCVA.

Assuming the study is sufficiently powered, the clinical impact will largely depend on the results of the study:

'Positive result' (surgery superior to anti-VEGF monotherapy at the defined target difference): A 210-participant, pan-European RCT will provide robust evidence to guide clinical practice. Patients who would otherwise receive only anti-VEGF monotherapy would be offered surgery instead, with a better visual outcome. The large number of recruiting sites and Key Opinion Leaders involved in this RCT should facilitate adoption directly, and through local and national guidance.

'Negative result' (surgery not superior to anti-VEGF monotherapy at the defined target difference): Patients can be spared the risk, discomfort and inconvenience of surgery, and healthcare providers can be spared the expense of surgery.

If our RCT demonstrates the safety and efficacy of anti-VEGF monotherapy in SMH then this may alter those guidelines that currently deny patients with SMH access to anti-VEGF therapy.

# 4. Trial Objectives and Design

## 4.1. Trial Objective

To assess the safety and efficacy of vitrectomy, subretinal TPA, gas tamponade and intravitreal anti-VEGF as a treatment for SMH secondary to exudative AMD, versus standard of care with anti-VEGF monotherapy.

## 4.2 Trial Design and Flow Diagram

TIGER is a phase 3, multicentre, pan-European, non-commercial, randomised, two-group, active control, superiority, observer-masked, surgical trial. It is summarized in the following diagram:

**TIGER Flow Diagram**

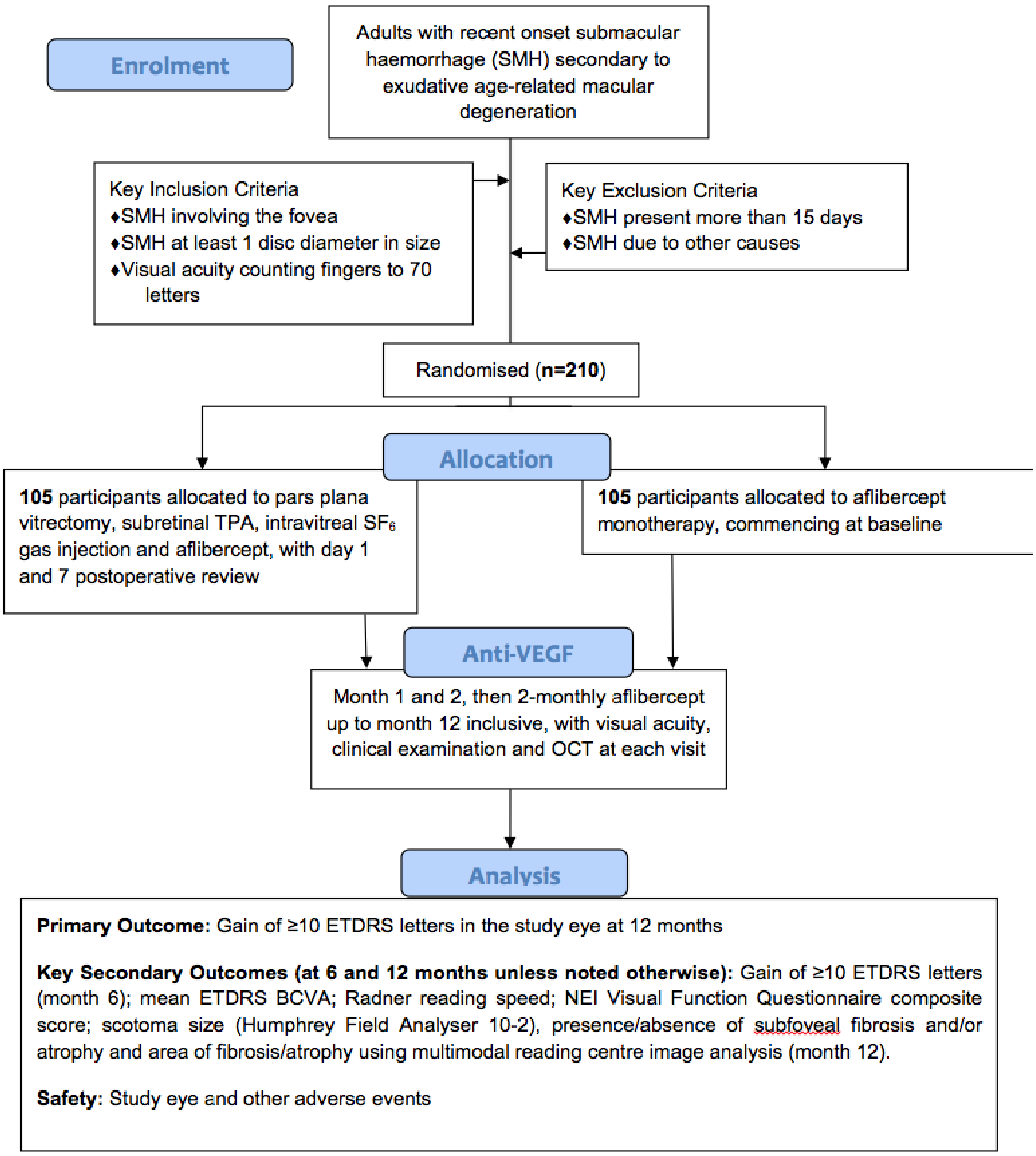

### 4.3 Trial Organisation

A simplified overview of TIGER's organisation is shown in the figure below.

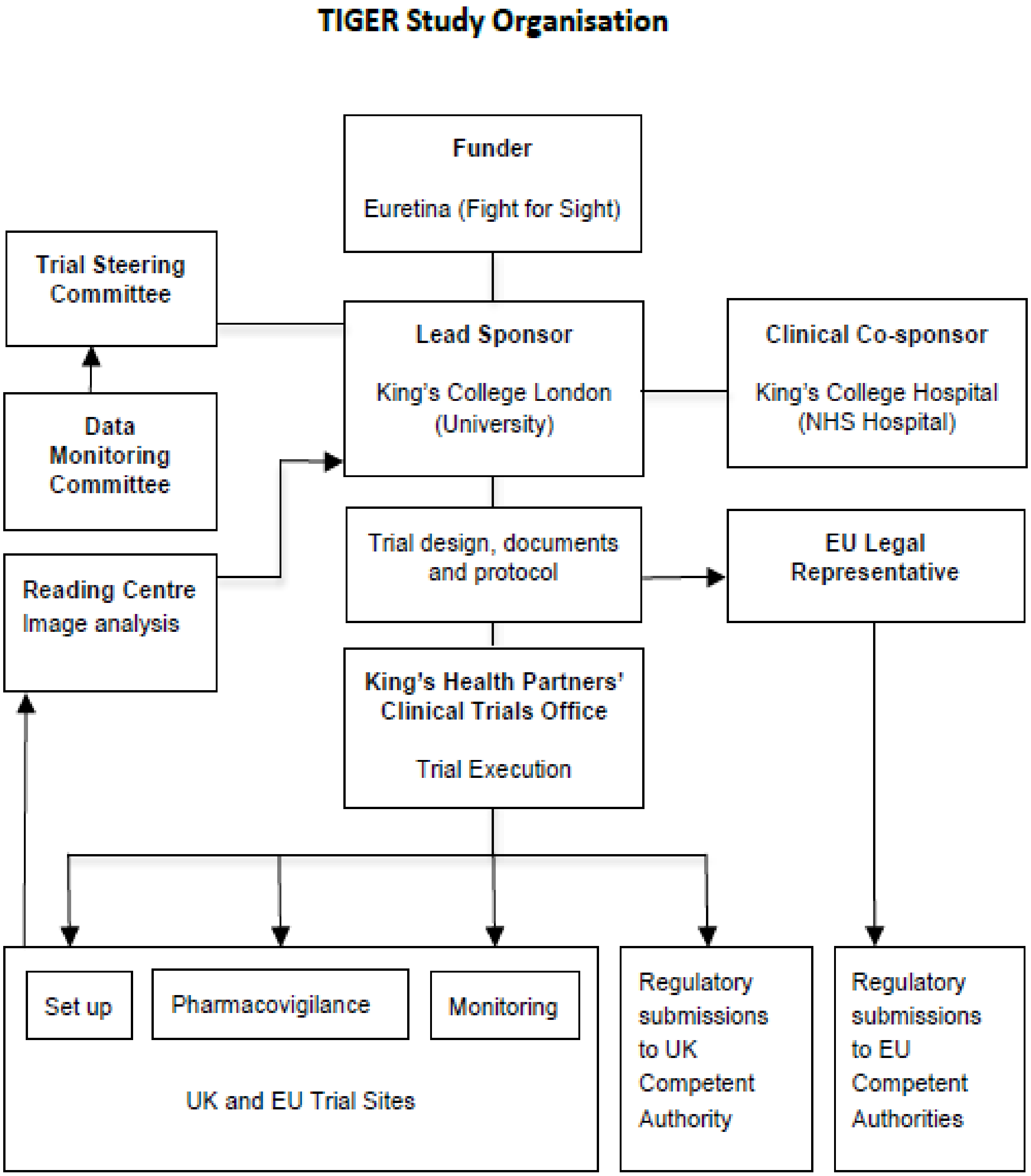

# 5. Trial Medication and Surgery

## 5.1 Investigational Medicinal Product (IMP): Tissue Plasminogen Activator (TPA, Alteplase)

Tissue plasminogen activator (TPA) is a 70k Da glycoprotein enzyme that activates plasminogen to plasmin, which in turn breaks down fibrin clots. Alteplase is a commercially produced TPA manufactured using a recombinant DNA technique and a Chinese hamster ovary cell line. Under the brand names Actilyse and Actilyse Cathflo marketed by Boehringer-Ingelheim, alteplase is licensed in the UK and EU for the treatment of myocardial infarction, acute ischaemic stroke, and pulmonary embolism (for 10 mg, 20 mg and 50 mg Actilyse) and for the thrombolytic treatment of occluded central venous access devices (for 2 mg Actilyse Cathflo) respectively. Alteplase is not licensed for the treatment of submacular clots. It is intended that TIGER will use vials containing 10 mg of Actilyse alteplase in powdered form, packaged with a diluent (10 mls of water for injection), or Actilyse Cathflo 2 mg in powdered form which contain 2.2 mg total dry weight of Alteplase active agent packaged without a diluent.

In the event that sites are unable to access Actilyse and Actilyse Cathflo, alternative forms of licensed alteplase that do not have marketing authorisation in the UK or EU may be used for TIGER, according to what sites have access to, and pending approval from the study management team. Brands of alteplase have the same excipients and are molecularly identical to Actilyse can be substituted for use in the TIGER study, for example:

- Activase alteplase 10 mg, 20 mg, 50 mg
- Activase Cathflo 2 mg
- Activacin 50 mg, 100 mg
- GRTPA 10 mg, 20 mg, 50 mg

In TIGER, alteplase TPA will be used off-label to dissolve submacular clots. We therefore expect TIGER will be a Clinical Trial of an Investigational Medicinal Project (CTIMP), although France's STAR trial (ClinicalTrials.gov Identifier: NCT02557451) was determined not to be a CTIMP (personal communication, Pr Catherine Creuzot, Chef du Service d'Ophtalmologie, CHU, Dijon). Each relevant Competent Authority will be asked to determine TIGER's status.

TIGER aims to select sites that already have access to alteplase for use within its marketing authorisation. Stock alteplase will be relabelled by the site's Trials Pharmacy according to Annex 13 of Good Manufacturing Practice. Sites will be provided with an Annex 13-compliant template label, which can be adapted in accordance with local and national requirements. Accordingly, alteplase will not need to be shipped to sites.

## 5.2 TPA (Alteplase) Dose, Injection Technique and Surgery

### 5.2.1 TPA (Alteplase) Dose

The *maximum dose* of alteplase TPA to be used in TIGER is 25 micrograms, delivered by subretinal injection.

The *concentration* of alteplase TPA to be used in TIGER is 100 micrograms in 1 ml. Using 10 mg Actilyse and 2 mg Actilyse Cathflo as examples, the methodology to make up this concentration is as follows:

*10 mg Actilyse*
For the 10mg Actilyse formulation, using sterile technique, this can be pre-prepared by injecting the 10 mls of the **water for injection** diluent that comes with 10mg Actilyse, into the 10 mg vial, then drawing up 1 ml of this solution and making up to 10 ml with **0.9% sodium chloride** for injection. This gives the desired concentration of 100 micrograms in 1 ml.

*2 mg Actilyse Cathflo*
For the 2mg Actilyse Cathflo formulation, which contains a total of 2.2 mg alteplase, this concentration can be prepared using 2.2 mls of sterile **water for injection** diluent which should be injected into the Actilyse Cathflo vial; draw up 1 ml of this solution and make up to 10 ml with **0.9% sodium chloride** for injection. This gives the desired concentration of 100 micrograms in 1 ml.

Larger dose vials are available e.g. Actilyse 50 mg, and can be used if necessary, but a 10 mg vial has far more drug than is required for subretinal injection and so larger vials are wasteful and best avoided. If Actilyse is not available, alternative, molecularly identical brands of alteplase can be substituted, subject to caveats in section 5.1 above.

Surgeons can inject up to 0.25 mls of the 100 micrograms in 1 ml solution (up to a maximum dose of 25 micrograms) under the retina as a single dose during surgery. The volume required depends on the amount needed to cover the submacular haemorrhage. Typically, most haemorrhages will require no more than 0.1 ml (10 micrograms). Record the total volume injected in the trial source data worksheets and electronic case report form (eCRF). TPA should not be injected under the RPE.

If there is a recurrent SMH then the treatment can be repeated, provided the participant still meets the eligibility criteria. Record repeat treatments in the TIGER source data worksheets and eCRF.

### 5.2.2 TPA (Alteplase) Injection and Surgical Technique, Required Surgeon Experience, and Timing of Surgery

Vitrectomy, subretinal TPA injection, and gas should be undertaken by a Consultant Vitreoretinal Surgeon who has performed the procedure before. Surgery can also be undertaken by a Senior Vitreoretinal Fellow provided he or she has performed at least 300 pars plana vitrectomies and has done the procedure before. If a Senior Vitreoretinal

Fellow or Consultant Vitreoretinal Surgeon have not done the procedure before they may treat a TIGER participant provided that their first case is directly supervised by a Consultant Vitreoretinal Surgeon who has done the procedure before.

As blood is rapidly toxic to photoreceptors excessive delay may mean surgery is less effective, and the surgical risks may start to outweigh the potential benefits. Therefore, **it is very important that both screening and surgery are expedited**. Ideally, screening is completed in 1 day and surgery scheduled within 3 days of confirmed eligibility. In cases where the SMH onset is known, it should not have been present for more than 15 days at the point in time when eligibility is confirmed. The maximum time between known SMH onset and surgery is 18 days, for example screening on days 13 and 14 after SMH onset, and surgery 4 days after that. If the onset of SMH is not known, then total time between the start of screening and surgery should be no more than 7 days (if the clinical features suggest SMH has been present >15 days patients are ineligible, even if this cannot be confirmed by history or pre-trial documentation). These allowances should not be used to delay surgery, which remains urgent in all cases.

Surgery involves the following steps:

- Anaesthesia may be local, with or without sedation, or general anaesthesia, based on patient preference and local practice.
- A full 3-port pars plana vitrectomy should be undertaken using 20- to 27-gauge ports.
- The hyaloid face should be separated from the retina, if it is not already.
- Indented vitrectomy (vitreous base shaving) may be undertaken at the surgeon's discretion.
- Phacoemulsification and intraocular lens implant may be performed based on the surgeon's and participant's preference, in accordance with local practice
- Peeling of epiretinal membrane (ERM) ± internal limiting membrane (ILM) is allowed if epiretinal membrane and macular pucker are present but should not be done prophylactically. Record that and ERM and/or ILM peel was undertaken.
- The concentration of alteplase TPA to be used in TIGER is 100 micrograms in 1 ml. This can be pre-prepared as noted above in Section 5.2.1.
- Transretinally inject up to 0.25 mls of this solution (maximum dose: 25 micrograms) as required, depending on the size of the submacular haemorrhage. Typically, most haemorrhages will require no more than 0.1 ml. Record the total volume injected in the TIGER source data worksheets and eCRF, excluding any reflux.
- Surgeons should use a subretinal injection cannula of 38-gauge or less. Several companies make these including MedOne, DORC and Synergetics, with marketing authorisation to use as required in this study. Connect this to a fine bore flexible plastic connecting tube. An example (from MedOne) is shown below.

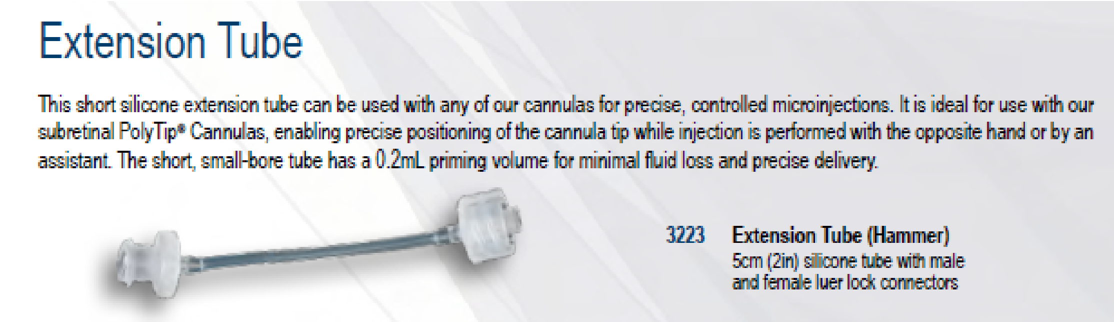


- Couple the subretinal injection cannula attached to the extension tube, and connect these to a 1 ml syringe containing alteplase. Prime the extension canula and extension tube.
- The alteplase can be manually injected by an assistant, or using an automated pneumatic injection system eg MedOne, MicroDose Injection Kit (https://medone.com/microdose-injection-device/).
- Aim to create a localised area of retinal detachment with the alteplase solution surrounding and enveloping the entire clot.
- When injecting choose an injection point(s) away from the foveal centre, but in an area of subretinal haemorrhage. The further from the fovea the lower the risk of macular hole formation. Avoid areas of pigment epithelial detachment to avoid sub-RPE injection, which increases the risk of an RPE rip.
- Multiple injection points are allowed if clinically indicated
- Check the peripheral retina carefully for retinal tears etc and if present treat with laser or cryotherapy as clinically indicated.
- Perform a full fluid/air exchange and then inject 0.05 ml of aflibercept (Eylea, Bayer).
- Perform an air/gas exchange of 20% sulfahexafluoride ($SF_6$) using at least 30 mls of gas. Surgeons can use their local supplier of $SF_6$. Since $SF_6$ is a commonly used surgical device licensed for intravitreal gas tamponade, it does not require trial labelling.
- Instruct participants how to posture after surgery (see next section).
- Prescribe post-operative eye drops as detailed on the next page.

### 5.3 Post-operative head positioning (posturing)

- Advise participants undergoing surgery to remain on their back for 15 minutes after the TPA injection, to allow clot liquefaction. This includes any time lying supine during surgery (after the TPA injection).
- After that, advise participants to sit up and lean face forward by 45 degrees during the day for 5 days, for 50 minutes out of every hour. During any break from posturing the participant's head should be upright. They should be mobile during some of their break time, to avoid deep vein thrombosis and to minimise stiffness.
- Advise participants to sleep on their side with the operated eye dependent eg if the right eye underwent surgery sleep right cheek to pillow. Night-time posture continues for 10 days after surgery. Having the head raised at night by two to three

pillows (whilst also maintaining the correct cheek to pillow) may further aid downward displacement and can be advised.

- Advise participants to avoid lying on their backs or leaning backwards for 10 days after surgery, to prevent inadvertent subfoveal displacement of the dissolved submacular clot.
- Occasionally, the posture might need to be altered due to the position of the submacular blood following TPA. For example, if the blood is predominantly nasal to the fovea the participant might be advised to sleep on the opposite cheek. Any atypical posturing should be documented.
- TIGER aims to be a pragmatic trial, so inability to completely adhere to these instructions is not an exclusion criterion, and poor compliance is not a deviation, however participants should be firmly encouraged to posture as best they can. Participants should be asked about their compliance with posturing at each visit for 10 days after surgery, and failure of comply with instructions should be noted in the trial source data worksheets and eCRF.
- Provide participants in the surgical arm with the following document:

  *Additional patient information: How to position your head after surgery*

## 5.4 Concomitant Treatments

All concomitant medications should be recorded in the source data worksheets eCRF as follows:

### 5.4.1. Post-operative eye drops

Prescribe broad-spectrum antibiotic eye drops for at least 1 week after surgery, and topical steroid eye drops for at least 4 weeks postoperatively. Mydriatics are allowed at the surgeon's discretion, for approximately 1-2 weeks. The choice of steroid, antibiotic and mydriatic is at the surgeon's preference, considering also any local policy and the particulars of each participant.

### 5.4.2 Aflibercept (Eylea)

Aflibercept (Eylea, Bayer) will be used for the treatment of the underlying exudative AMD, reflecting standard of care.

Eylea use within TIGER is considered to be a non-Investigational Medicinal Product (nIMP) as it is a background treatment for wet AMD, whereas the object of the trial is to assess surgery (with TPA) against no surgery for SMH. The Eylea is used fully within its marketing authorisation, and given to both arms of the study. Patients with wet AMD would typically be receiving an anti-VEGF therapy such as Eylea irrespective of their enrolment in TIGER.

Both arms of the study will be dosed with a 0.05 ml intravitreal injection of 2 mg Eylea monthly for three doses, then 2-monthly out to month 12 with a minimum window of 21 days between Eylea treatments.

At screening, there may be situations where a participant has already received a treatment of anti-VEGF in the preceding weeks. If a patient has been randomised to surgery they should proceed with surgery regardless of when the preceding anti-VEGF was delivered, and receive aflibercept after fluid-air exchange. This is because any remaining anti-VEGF drug within the vitreous will be removed during surgery.

For participants randomised to the control arm:

- If a participant received an anti-VEGF treatment 3 weeks ago, they should have their baseline Eylea either on the same day or within 7 days following screening and continue per protocol dosing.

- If a participant received an anti-VEGF treatment 2 weeks ago, they should have their baseline Eylea 7 days following screening and continue per protocol dosing.

- If a patient received an anti-VEGF treatment 1 week ago or less, no treatment should be given at screening/baseline and the source data worksheets updated accordingly. The first treatment for the trial should be given at month 1. From there, treatment should be continued as indicated for the trial.

Bayer will provide Eylea vials free of charge or sites may elect to use their own supplies. Storage, contraindications, precautions, undesirable effects, pharmacological properties, and instructions for use etc are detailed at https://www.medicines.org.uk/emc/product/2879/smpc. For participants in the surgical arm, the first injection of Eylea (2mg) occurs towards the end of surgery, after fluid-air exchange. Sites may elect to label the Eylea for trial use, or use standard hospital prescriptions and dispensing, in accordance with local preference/policy and the terms and conditions of Bayer's provision of free Eylea to each site (if the site has elected to use Bayer's free supply).

The manufacturer provides a guide to Eylea for patients with wet AMD, available at: https://www.medicines.org.uk/emc/rmm/617/Document. Sites are encouraged to provide this or their own information on Eylea to participants, but it should be explained that the co-existence of SMH will adversely affect prognosis.

Patients currently receiving another anti-VEGF drug will need to swap to Eylea for the duration of the study, and this should be discussed with them. Likewise, arrangements for continuation of Eylea at the end of the trial, or change to another anti-VEGF agent, should be discussed with the patient, so that they are aware of the locally available anti-VEGF agents.

## 5.5 Other permitted treatments for cataract, age-related macular degeneration and idiopathic polypoidal choroidal vasculopathy

Treatment for age-related macular degeneration, idiopathic polypoidal choroidal vasculopathy and cataract should be limited to the following, but treatment for fellow eye or systemic diseases should be as clinically indicated:

### 5.5.1 Photodynamic Therapy (PDT) for Idiopathic Polypoidal Choroidal Vasculopathy (IPCV)

Polyps can be treated with PDT, if that is the site's usual practice. Aflibercept therapy should continue.

The reading centre's determination of whether or not polyps are present is not to be communicated to sites. This is so that a given site's ability to detect and treat polyps with PDT will reflect real-world decisions, making the results of TIGER more generalisable. Therefore, the site should make their own determination as to whether or not polyps are present, and if polyps are found, they should likewise make their own decision whether or not to offer their participant PDT.

### 5.5.2 Reoperation for Recurrent Submacular Haemorrhage

A Scottish Ophthalmic Surveillance Unit (SOSU) study estimated that 20% of cases have a rebleed within 6 months, at a mean time of 96 days.[2] For those who do repeat surgery (vitrectomy, subretinal TPA, $SF_6$ and anti-VEGF) is permitted provided the TIGER eligibility criteria are still met, and they were originally randomised to the surgical arm. More than one repeat surgery is permitted if eligible SMH recurs more than once. Participants originally allocated to the control arm should continue on aflibercept monotherapy.

### 5.5.3 Cataract surgery (Including Phakovitrectomy)

At the first TIGER study group meeting some surgeons advocated phakovitrectomy to avoid the cost and inconvenience of two operations; others preferred sequential cataract surgery as biometry is more reliable after the SMH resolves. Either are allowed, to reflect local standard of care. Regular lens grading will be undertaken during follow-up visits and should be recorded in the trial source data worksheets and eCRF, for both arms of the study. Investigators are encouraged to promptly treat emerging cataract in either arm, at least 8 weeks prior to the month 12 visit.

It is recognised that removal of pre-existing subclinical lens opacity, and conversely failure to remove developing post-vitrectomy cataract, may both alter BCVA. However it is not desirable to exclude phakic patients or cataract surgery, as that would reduce recruitment and trial generalisability respectively. It is understood that any vision gain in the surgical group may come partly from cataract surgery, but the trial considers the surgical pathway under investigation to include cataract surgery as needed. A subgroup

analysis in pseudophakes will be undertaken, and aims to help understand the mechanism of any vision gain.

### 5.6 IMP Risks

The known systemic risks of Actilyse are detailed in the Actilyse powder and solvent solution for injection Summary of Medical Product Characteristics (SmPC). Section 4.8 of this SmPC as approved will be used as Reference Safety Information for both formulations. However, these risks relate to the treatment of stroke, myocardial infarction (MI), and pulmonary embolus (PE).

Actilyse is not licensed for use in the eye. The dose used for the treatment of SMH is much lower. For example, the dose used to treat PE is 100 mg, whereas the maximum dose delivered within TIGER is 25 micrograms (1/4000$^{th}$ dose). Further the drug is delivered subretinally, within the blood-ocular-barrier, rather than directly into the systemic circulation. Furthermore, Actilyse has a very short half-life (4-5 minutes in plasma). Hence systemic drug levels are thought very unlikely to pose a risk outside of the eye. Nonetheless, we retain the undesirable effects described in the SmPC as 'expected'. Additional 'expected' risks based on the anticipated, accepted or reported risks particular to the action and/or delivery of Actilyse in the eye include:

- Ocular haemorrhage including, but not limited to, pre-retinal, retinal, subretinal, sub-retinal pigment epithelium (RPE), choroidal, suprachoroidal, vitreous, iris, drainage angle-haemorrhage, anterior chamber (including hyphaema), and subconjunctival haemorrhage.
- Ocular or systemic hypersensitivity reaction.
- Ocular inflammation, including, but not limited to choroiditis, retinitis, retinal vasculitis, vitritis, and uveitis including anterior, intermediate, posterior, and pan-uveitis, as well as iritis and pars planitis.
- Sterile or culture-positive endophthalmitis are thought possible, but likely to be rare events.
- Raised intraocular pressure (IOP) and glaucoma
- Lens opacity and cataract.
- Toxicity is possible, and could manifest as ocular inflammation, retinal or macular ischaemia, retinal or macular oedema, cotton wool spots (retinal nerve fibre layer infarcts), and retinal vessel occlusion (including central or branch retinal vein occlusion and central or branch retinal artery occlusion).
- There are risks related to the injection of a subretinal fluid volume, including macular hole, retinal tear, RPE tear, and raised IOP.

- The injection procedure, in distinction to a drug effect, could cause cataract and retinal trauma including a tear, hole or haemorrhage of the retina, macula, RPE or choroid.

- These conditions, or others, could lead to loss of vision and eye pain, periocular pain or headache.

Because the systemic drug levels of Actilyse will be low following intraocular use the systemic contraindications to Actilyse use listed in the SmPC do not apply, except for known allergies to Actilyse, gentamicin (a trace residue from the manufacturing process) and excipients (see exclusion criteria).

In the event an alternative, non-Actilyse brand of alteplase is used, it is anticipated that if the manufacturing process is identical to Actilyse, and the drug is molecularly identical to Actilyse with the same excipients, the IMP risks will be same as laid out in the SmPC. Reference safety information for non-Actilyse alteplase is available in the respective monographs for each brand of alteplase. Use of other alteplase brands for TIGER will be subject to caveats as laid out in section 5.1.

### 5.7 Drug Accountability and Disposal

Actilyse is supplied as a single 10 mg vial, which contains more drug than is needed for the single subretinal injection administered during surgery. The drug will be labelled by the Trials Pharmacy and then supplied directly to the operating team, most likely on the day of surgery. Alternatively, storage of Actilyse can be delegated by the local Trials Pharmacy according to the Site's usual practice, for example, storage in the operating theatre. Temperature must be monitored and full accountability and temperature records made available to the Sponsor clinical research associate (CRA), when requested, for verification purposes.

In the event an alternative, non-Actilyse brand of alteplase is approved for use at the site subject to caveats as laid out in section 5.1, these instructions for Drug Accountability and Disposal apply equally.

The drug should be reconstituted in theatre shortly before it is administered. If a site's local policy is for TPA to be reconstituted in a specified clean area or room, this would be permissible on a site-by-site basis. The reconstituted TPA should then be delivered to the operating theatre shortly before it is administered. The operating surgeon should record that TPA was administered (or the reason it was not), the volume injected and the batch number in the trial source data worksheets (but not the eCRF). There are no special requirements in terms of drug disposal, and the remaining drug can be disposed of in accordance with local practice.

### 5.8 Storage of IMP

According to the Summary of Medical Product Characteristics (SmPC) for both Actilyse and Actilyse Cathflo:

“Unopened, Actilyse has a shelf life of 3 years. The reconstituted solution has been demonstrated to be stable for 24 hours at 2°C – 8°C and for 8 hours at 25°C.

From a microbiological point of view, the product should be used immediately after reconstitution.

If not used immediately, in-use storage times and conditions prior to use are the responsibility of the user and would normally not be longer than 24 hours at 2 to 8°C. “Do not store above 25°C”.

For the purposes of TIGER, Actilyse should be reconstituted in theatre under sterile conditions immediately prior to, or during surgery, having first confirmed the drug’s expiry date has not passed. The vial should be inspected prior to injection to ensure there is no particulate matter. The solution should be clear or pale yellow. As stated in Section 5.7, if a site’s usual practice is for TPA to be reconstituted in a specified clean area or room and delivered to the operating theatre, this should happen shortly before administration and the operating surgeon should confirm expiration date and inspect the solution prior to injection.

In the event an alternative, non-Actilyse brand of alteplase is approved for use at the site subject to caveats as laid out in section 5.1, storage instructions should defer to the respective drug monographs as they may differ to the Actilyse SmPC.

# 6. Selection and Withdrawal of Subjects

## 6.1 Inclusion Criteria

***General***

1. Males or females aged at least 50 years

***Study eye***

2. SMH, comprising sub-neuroretinal haemorrhage with or without sub-RPE haemorrhage, that occurs secondary to treatment naïve, or previously treated exudative AMD, including choroidal neovascularisation (CNV), idiopathic polypoidal choroidal vasculopathy (IPCV) and retinal angiomatous proliferation (RAP).

3. SMH involving the foveal centre that measures at least 1 disc diameter in greatest linear dimension.

4. Sub-neuroretinal haemorrhage at least 125 microns thick, measured at the foveal centre using spectral-domain optical coherence tomography (SD-OCT).

5. BCVA between counting fingers and an Early Treatment of Diabetic Retinopathy Study (ETDRS) letter score of 70, inclusive.

## 6.2 Exclusion Criteria

***General***

1. Serious allergy to fluorescein or indocyanine green (ICG).

2. Hypersensitivity to alteplase, gentamicin, arginine, phosphoric acid, polysorbate 80 or aflibercept (Eylea).

3. Stroke, transient ischaemic attack or myocardial infarction within 6 months.

4. Participation in another interventional study within 12 weeks of enrolment or planned to occur during this study.

5. Women who are breast feeding, pregnant, or planning to become pregnant during the clinical trial. Any sexually active women of childbearing potential must agree continued abstinence from heterosexual intercourse or to use highly effective methods of birth control for the duration up to 12 weeks post IMP administration. Men must also agree to use a condom if their partner is of child bearing potential, even if they have had a successful vasectomy. Females of childbearing potential are females who have experienced menarche and are not surgically sterilised (e.g. hysterectomy or bilateral salpingectomy) or post-menopausal (defined as at least 1 year since last regular menstrual period). Highly effective methods of birth control are those with a failure rate of < 1% per year when employed consistently and correctly, eg. combined (oestrogen and progestogen containing) hormonal contraception associated with inhibition of ovulation via oral, intravaginal, and transdermal routes; progestogen-only hormonal contraception associated with inhibition of ovulation via oral, injectable, implantable, intrauterine device (IUD), or intrauterine hormone-releasing system ( IUS); or vasectomised partner.

6. International Normalised Ratio (INR) greater than 3.5, unless it is anticipated that the INR can be brought below this level prior to vitrectomy, balancing the systemic risks with those of intraocular haemorrhage.

7. Unwilling, unable, or unlikely to return for scheduled follow-up for the duration of the trial.

8. Any other condition which, in the opinion of the investigator, would prevent the participant from granting informed consent or complying with the protocol, such as dementia, mental illness, or serious systemic medical disease.

***Study eye***

9. SMH that is known or estimated to have been present for longer than 15 days, as evidenced by history, pre-trial clinical documentation, or fundus appearance.
10. SMH due to eye disease other than exudative AMD.
11. Current active proliferative diabetic retinopathy.
12. Current intraocular inflammation.
13. Current ocular or periocular infection other than blepharitis.
14. Current or known former high myopia (>6 dioptres).
15. Aphakia.
16. Other current or pre-existing ocular conditions that, in the opinion of the Investigator, will preclude any improvement in BCVA following resolution of SMH, such as severe central macular atrophy or fibrosis, dense amblyopia, macular hole involving the fovea, or very poor BCVA prior to presentation with SMH (counting fingers or worse).
17. Inadequate pupillary dilation or significant media opacities, which will prevent adequate clinical evaluation of the posterior segment or fundus imaging.
18. Intraocular surgery within 12 weeks of enrolment except for uncomplicated cataract surgery, which is permitted within 8 weeks of enrolment.

## 6.3 Selection of Participants

It is anticipated that most participants will be identified from the clinics of the Investigators, or by referral to the Investigator for the treatment of acute SMH. Referrals may arise from family physicians, optometrists, and ophthalmologists. Research networks may also identify suitable patients. Patients diagnosed with SMH are welcome to contact any participating site directly, as speed of referral is important, but ideally they will arrive with a referral letter detailing the presenting complaint and past ocular/systemic/drug history.

## 6.4 Emergency Code Break

Emergency code break is not required, as most participants in the surgical arm will receive only a single dose of TPA, and both arms of the study are unmasked (it is not possible to undertake a sham vitrectomy, TPA and gas).

## 6.5 Withdrawal of Subjects

Participants have the right to withdraw from the study at any time for any reason. The investigator also has the right to withdraw patients from the study drug in the event of inter-current illness, adverse events (AEs), serious adverse events (SAEs), suspected, unexpected, serious adverse reactions (SUSARs), lack of mental capacity, protocol violations, cure, burden of care, administrative reasons, or other reasons.
Retention of participants, however, is essential for the success of the trial so unnecessary withdrawals should be discouraged. Should a participant decide to withdraw from TIGER, all efforts will be made to report the reason for withdrawal as thoroughly as possible. Participants must not be coerced to remain in the study against their will, but it should be explained that safety monitoring is likely to be more vigilant within the study than outside it.

If participants wish to withdraw from the study they should be invited to complete an Exit Visit, which collects the data that is usually acquired at Month 12, but they should not be pressured to accept this invite and can withdraw at any stage without further testing or data collection.

### 6.6 Recruitment Timelines and Trial Duration

The estimated annual incidence of large SMH meets the criteria of a rare disease[4] and recruitment estimates need to be realistic. At an initial TIGER study group meeting sites' recruitment estimate averaged 0.83 participants/month. However, sites often fail to deliver as predicted. Our TAPAS pilot averaged 0.28 participants/site/month, but with 65 sites TIGER cannot choose sites as selectively, so we estimate only 0.14 participants/site/month. This matches the recruitment numbers of the STAR study (ClinicalTrials.gov Identifier: NCT02557451; personal communication, Pr Catherine Creuzot, Chef du Service d'Ophtalmologie, CHU, Dijon). Estimated participant and site recruitment are shown below:

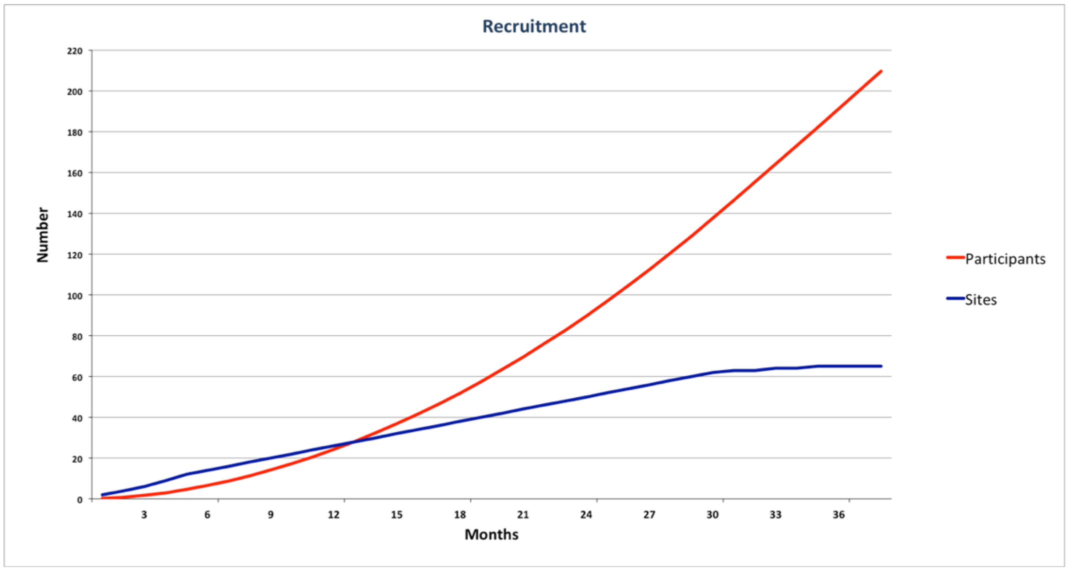

Participants are followed-up for 12 months on-study. Following the last participant's last visit, TIGER estimates 2.5 months for data cleaning and data lock, and then 2.5 months to analyse the main dataset.

Trial end is defined as data lock. This is estimated to occur in April 2025. Write-up and submission of the study results to a peer reviewed publication occurs subsequently.

In total, the research grant (as distinct to the trial duration) is expected to run for 5 years, starting September 2020, to include set-up, recruitment, follow-up, data lock, analysis and write-up.

## 7. Schedule of Activities

| Activity | Screening** | Baseline** | D1 | W1 | M1 | M2 | M4 | M6 | M8 | M10 | M12 |
|---|---|---|---|---|---|---|---|---|---|---|---|
| Visit window (±days) | Day -7 to 0 | - | 0 | ±3 | ±7 | ±7 | ±7 | ±7 | ±7 | ±7 | ±7 |
| Consent | X | | | | | | | | | | |
| Medical and ophthalmic history | X | | | | | | | | | | |
| Randomisation | X | | | | | | | | | | |
| Vitrectomy, TPA and gas (Arm A only) | | Arm A[§] | | | | | | | | | |
| Intravitreal aflibercept | | X[§] | | | X | X | X | X | X | X | X |
| Full refracted ETDRS BCVA[$] | X | | | | | | | X | | | X |
| Clinic ETDRS VA[†] | | | Arm A | Arm A | X | X | X | | X | X | |
| Radner reading vision[‡] | X | | | | | | | X | | | X |
| Visual field (HFA 10-2)(sent to reading centre)[¢] | X | | | | | | | X | | | X |
| Slit-lamp examination and intraocular pressure (IOP)[Ø] | X | | Arm A | Arm A | X | X | X | X | X | X | X |
| Lens grading[#] | X | | | | | | X | X | | X | X |
| VFQ-25, EQ-5D-5L and SWEMWBS questionnaires[¥] | X | | | | | | | X | | | X |
| Service user questionnaire | | | | | X | X | X | X | X | X | X |
| Fluorescein angiography (sent to reading centre) [completed once only-see footnote]* | Can be delayed until SMH clears sufficiently* | | | | * | * | * | * | * | * | * |
| ICG angiography (sent to reading centre) [completed once only-see footnote]* | Can be delayed until SMH clears sufficiently* | | | | * | * | * | * | * | * | * |
| OCT (sent to reading centre)[!] | X | | | | | | | | | | X |
| Clinic OCT (not sent to reading centre)[!] | | | | | X | X | X | X | X | X | |
| Stereo fundus photography (sent to reading centre)* | X* | | | | | | | | | | X |
| Autofluorescence (sent to reading centre)* | X* | | | | | | | | | | X |
| Adverse events (safety) | X | X | Arm A | Arm A | X | X | X | X | X | X | X |
| Concomitant medications | X | X | Arm A | Arm A | X | X | X | X | X | X | X |

## 7.1 Schedule of Activities by Visit Table

Items in **red** occur primarily due to participation in TIGER, others are likely to occur regardless of trial participation but data will nonetheless be collected on the trial source data worksheets and electronic case report forms (eCRFs). Surgery (Arm A) may or may not be standard of care depending on the eye unit. D1=day 1; W1=week 1; M1= calendar month 1.

Participants withdrawing early should be invited, but not pressured, to attend an optional Exit Visit mirroring month 12 data collection.

**As blood is rapidly toxic to photoreceptors excessive delay may mean surgery is less effective, and the surgical risks may start to outweigh the potential benefits. Therefore, **it is very important that both screening and surgery are expedited**. Ideally, screening is completed in 1 day and surgery scheduled within 3 days of confirmed eligibility. Screening and surgery can occur on the same day, to avoid delay. In cases where the SMH onset is known, it should not have been present for more than 15 days at the point eligibility is confirmed. The maximum time between known SMH onset and surgery is 18 days, for example screening on days 13 and 14 after SMH onset, and surgery 4 days after that. If SMH onset is unknown, then the total time between the start of screening and surgery should be no more than 7 days (if the clinical features suggest SMH has been present >15 days patients are ineligible, even if this cannot be confirmed by history or pre-trial documentation). These allowances should not be used to delay surgery, which remains urgent in all cases.

**$** Full refracted ETDRS VA is undertaken in both eyes separately at baseline and month 12 and study eye only at month 6. Details in Appendix A.

† Clinic ETDRS VA should be undertaken in the study eye only using an ETDRS chart with correction of any refractive error, with and without pinhole. Details in Appendix A.

‡ Radner reading vision should be measured in the study eye only at screening, month 6, and month 12. Details in Appendix A.

**¢** Visual field tests should be completed in study eye only, scanned, and sent to the Reading Centre, as detailed in Appendix C.

**Ø** Slit lamp examination and IOP should be undertaken in the both eyes at screening, month 6 and month 12, and study eye only at other visits.

# Lens grading is in both eyes at screening and month 12, but study eye only at months 4, 6 and 10. Details in Appendix B.

¥ The VFQ-25 questionnaire includes the ‘optional’ questions listed at the end in the appendix. The EuroQol questionnaire includes the 5-item vision bolt-on (EQ-5D-5L). SWEMWBS=Short Warwick-Edinburgh Mental Well-being Scale.

§ The initial intravitreal aflibercept injection can be administered on the day of screening, after eligibility is confirmed, in those randomised to the non-surgical group. In those randomised to surgery (Arm A), aflibercept is injected towards the end of surgery, straight after fluid-air exchange.

**!** At screening and month 12 ‘per protocol’ OCT images should be obtained in both eyes using certified staff and equipment, as per the Reading Centre’s instructions. These images are sent to the Reading Centre. At other visits a ‘Clinic OCT’ should be acquired in the study eye only, using standard staff and methodology. ‘Clinic OCT’ images are not sent to the reading centre. The attending clinical investigator should review all OCTs to monitor progress, watch for any emergent adverse events, and measure subretinal haemorrhage height. The same OCT machine should be used on a given participant throughout the study.

*Fluorescein and ICG angiography should be acquired in both eyes, once only. Either or both should be delayed if needed, to allow the SMH to clear sufficiently to enable visualisation of choroidal neovascularisation and/or polyps. The screening stereo fundus photography and fundus

autofluorescence (FAF), undertaken in both eyes, should be repeated with delayed angiography, to help interpretation by the Reading Centre. Clinical investigators should review imaging to detect any emergent adverse events

## 7.2 Procedures by Visit

For visit windows and timings see the table on the previous page. The week 1 visit (Arm A only) can be ±3 days (day 4-10) and the monthly visits ± 7 days. The month label represents calendar months. For example, a participant seen 21st September would return 21st October for their next monthly appointment.

This section details the procedures required for this study, but other procedures should be performed if and as clinically indicated.

### 7.2.1 Screening and Baseline Treatment

Patients have as long as they need to consider participation, within the eligibility timelines, but same day enrolment is permitted as SMH is rapidly toxic.

Screening activity comprises written informed consent (before any other activity occurs), collection of medical, ophthalmic and drug history, full objective and subjective refraction (Appendix A) in both eyes, full refracted ETDRS BCVA starting at 4m in both eyes (Appendix A), Radner reading speed in the study eye (Appendix A), Humphrey 10-2 visual field (HFA 10-2 VF; Appendix C) in the study eye (sent to the independent reading centre, NetwORC UK), National Eye Institute 25 item visual function questionnaire (NEI VFQ-25 including additional 'optional' questions in appendix), and EQ-5D-5L and Short Warwick-Edinburgh Mental Well-being Scale (SWEMWBS) questionnaires, slit-lamp examination including dilated fundoscopy and intraocular pressure (IOP) measurement in both eyes, lens grading in both eyes (Appendix B) and recording of any adverse events occurring after consent.

Fluorescein and indocyanine green (ICG) angiography in both eyes (either or both can be delayed as long as necessary to allow the SMH to clear sufficiently to enable visualisation the underlying structures), SD-OCT in both eyes, stereo fundus photography in both eyes and fundus autofluorescence (FAF) in both eyes; all these images will be sent to the independent reading centre (NetwORC UK) for masked assessment, but the attending clinical investigator should review them to look for clinical issues.

The attending clinical investigator should record the study eye centre point subretinal haemorrhage thickness using the calliper function of the OCT, measuring from the outer boundary of the ellipsoid layer to inner boundary of the RPE, and the central 1 mm subfield thickness (CST) after correction of any segmentation errors.

Eligible participants will be randomised 1:1 to:

1. Arm A: Surgery with aflibercept at the end of surgery, with post-operative review day 1 and week 1 (day 7) or,
2. Arm B: Aflibercept monotherapy commencing at baseline.

As noted in the footnote to Table 7.1, blood is rapidly toxic to photoreceptors excessive delay may mean surgery is less effective, and the surgical risks may start to outweigh the potential benefits. Therefore, **it is very important that both screening and surgery are expedited**. Ideally, screening is completed in 1 day and surgery scheduled within 3 days of confirmed eligibility. Screening and surgery are permitted on the same day, to reduce delay. In cases where the SMH onset is known, it should not have been present for more than 15 days at the point in time when eligibility is confirmed. The maximum time between known SMH onset and surgery is 18 days, for example screening on days 13 and 14 after SMH onset, and surgery 4 days after that. If the onset of SMH is not known, then total time between the start of screening and surgery should be no more than 7 days (if the clinical features suggest SMH has been present >15 days patients are ineligible, even if this cannot be confirmed by history or pre-trial documentation). These allowances should not be used to delay surgery, which remains urgent in all cases. Baseline is defined as the date of surgery for Arm A, and the date of aflibercept administration for Arm B. Baseline can occur on the same day as successful screening.

### 7.2.2 Day 1 and 7 (Arm A and study eye only)

Day 1 and week 1 (day 7) postoperative review, for those in the surgical arm, comprises Clinic ETDRS VA in the study eye (details in Appendix A), slit-lamp examination and IOP of the study eye, and recording of concomitant medications (ConMeds) and adverse events (AEs) including any new medical diagnoses.

### 7.2.3 Month 1 and 2

Both groups return Month 1 and 2 for Clinic ETDRS VA in the study eye (details in Appendix A), slit-lamp examination and IOP in the study eye, clinic OCT in the study eye (not sent to the reading centre), service user questionnaire, intravitreal injection of aflibercept in the study eye, ConMeds and AEs.

The attending clinical investigator should record the study eye centre point subretinal haemorrhage thickness using the calliper function of the OCT, measuring from the outer boundary of the ellipsoid layer to inner boundary of the RPE. If no haemorrhage is present record zero.

Delayed aniography: If fluorescein and ICG angiography were not obtained at screening/baseline, then they should be completed in both eyes when the SMH has cleared sufficiently to image the underlying structures, along with repeat stereo fundus photography in both eyes and autofluorescence in both eyes to facilitate their interpretation by the reading centre. Fluorescein and ICG angiography should be acquired only once, to characterise the underlying disease; both should be sent to the independent reading centre.

### 7.2.4 Month 4

Clinic ETDRS VA in study eye (Appendix A), slit-lamp examination and IOP in study eye, lens grading in study eye (Appendix B), clinic OCT (not sent to the reading centre)

in study eye, service user questionnaire, intravitreal injection of aflibercept in study eye, ConMeds and AEs.

The attending clinical investigator should record the centre point subretinal haemorrhage thickness using the calliper function of the OCT, measuring from the outer boundary of the ellipsoid layer to inner boundary of the RPE. If no haemorrhage is present record zero.

Delayed aniography: If fluorescein and ICG angiography were not obtained at a previous visit, then they should be completed in both eyes when the SMH has cleared sufficiently to image the underlying structures, along with repeat stereo fundus photography in both eyes and autofluorescence in both eyes to facilitate their interpretation by the reading centre. Fluorescein and ICG angiography should be acquired only once, to characterise the underlying disease; both should be sent to the independent reading centre.

**7.2.5 Month 6**

Full refracted ETDRS BCVA in study eye (Appendix A), Radner reading speed in study eye (Appendix A), Humphrey 10-2 visual field in the study eye (sent to reading centre; Appendix C), VFQ-25, EQ-5D-5L, SWEMWBS and service user questionnaires, slit-lamp examination and IOP in both eyes, lens grading in study eye (Appendix B), clinic OCT in study eye (not sent to the reading centre), intravitreal injection of aflibercept in study eye, ConMeds and AEs.

The attending clinical investigator should record the study eye centre point subretinal haemorrhage thickness using the calliper function of the OCT, measuring from the outer boundary of the ellipsoid layer to inner boundary of the RPE. If no haemorrhage is present record zero.

Delayed aniography: If fluorescein and ICG angiography were not obtained at a previous visit, then they should be completed in both eyes when the SMH has cleared sufficiently to image the underlying structures, along with repeat stereo fundus photography in both eyes and autofluorescence in both eyes to facilitate their interpretation by the reading centre. Fluorescein and ICG angiography should be acquired only once, to characterise the underlying disease; both should be sent to the independent reading centre.

**7.2.6 Month 8**

Clinic ETDRS VA in study eye (Appendix A), slit-lamp examination and IOP in study eye, clinic OCT in study eye (not sent to the reading centre), service user questionnaire, intravitreal injection of aflibercept in study eye, ConMeds and AEs.

The attending clinical investigator should record the study eye centre point subretinal haemorrhage thickness using the calliper function of the OCT, measuring from the outer boundary of the ellipsoid layer to inner boundary of the RPE. If no haemorrhage is present record zero.

Delayed aniography: If fluorescein and ICG angiography were not obtained at a previous visit, then they should be completed in both eyes when the SMH has cleared sufficiently to image the underlying structures, along with repeat stereo fundus photography in both eyes and autofluorescence in both eyes to facilitate their interpretation by the reading centre. Fluorescein and ICG angiography should be acquired only once, to characterise the underlying disease; both should be sent to the independent reading centre.

**7.2.7 Month 10**

Clinic ETDRS VA in study eye (Appendix A), slit-lamp examination and IOP in study eye, lens grading in study eye (Appedix B), clinic OCT in study eye (not sent to the reading centre), service user questionnaire, intravitreal injection of aflibercept in study eye, ConMeds and AEs.

The attending clinical investigator should record the study eye centre point subretinal haemorrhage thickness using the calliper function of the OCT, measuring from the outer boundary of the ellipsoid layer to inner boundary of the RPE. If no haemorrhage is present record zero.

Delayed aniography: If fluorescein and ICG angiography were not obtained at a previous visit, then they should be completed in both eyes when the SMH has cleared sufficiently to image the underlying structures, along with repeat stereo fundus photography in both eyes and autofluorescence in both eyes to facilitate their interpretation by the reading centre. Fluorescein and ICG angiography should be acquired only once, to characterise the underlying disease; both should be sent to the independent reading centre.

**7.2.8 Month 12 (final visit)**

Full refracted ETDRS BCVA in both eyes (Appendix A), Radner reading speed in study eye (Appendix A), Humphrey 10-2 visual field in study eye (sent to reading centre; Appendix C), NEI VFQ-25, EQ-5D-5L, SWEMWBS and service user questionnaires, slit-lamp examination and IOP in both eyes, lens grading in both eyes (Appendix B), intravitreal injection of aflibercept in study eye, ConMeds and AEs. OCT, stereo fundus photography and fundus autofluorescence (FAF) will all be acquired in both eyes and sent to the reading centre.

The attending clinical investigator should record the study eye centre point subretinal haemorrhage thickness using the calliper function of the OCT, measuring from the outer boundary of the ellipsoid layer to inner boundary of the RPE. If no haemorrhage is present record zero. Also record the central 1 mm subfield thickness (CST) after correction of any segmentation errors.

Delayed angiography: If either of fluorescein and ICG angiography were not obtained at a previous visit then they should be completed in both eyes at this visit. Both fluorescein and ICG angiography are required, but each is required only once; both should be sent to the independent reading centre.

### 7.3 Reading Centre Image Analysis

Ocular images and the Humphrey 10-2 visual fields tests acquired at the baseline and month 12 visits are sent to the NetwORC UK Reading Centres for analysis. Humphrey 10-2 visual field tests from month 6 are also sent to the reading centre This will enable independent, masked assessment of baseline characteristics and provide the key structural outcomes. The clinical investigators should also review the ocular images, as they may reveal important clinical features. For example, the baseline ICG angiogram (which, like the fluorescein angiogram, can be delayed as long as necessary to enable SMH to clear and improve visualisation of underlying structures), may show idiopathic polypoid choroidal vasculopathy and this may warrant a discussion with the participant about the option of photodynamic therapy (see section 5.6, Other permitted treatments).

Standardized imaging procedures are defined in the certification and examination manuals of the NetwORC UK Reading Centre. Image evaluation will be defined in the Reading Protocols of the NetwORC UK Reading Centre established for TIGER.

The address and contact details for the NetwORC UK Reading Centre are available at: https://www.networcuk.com/

OCT images captured at times other than baseline, and month 12 can be completed using standard clinic methodology, staff and equipment, as they do not form part of the primary or secondary outcome analyses, and nor do they define any anti-VEGF retreatment decisions. They do however form an important part of each participant's clinical and safety evaluation and each should be reviewed carefully by the attending clinical investigator.

#### 7.3.1 Delayed angiography

Fluorescein and ICG angiography (either or both) should be delayed if needed, to allow the SMH to clear sufficiently to enable visualisation of underlying choroidal neovascularisation and/or polyps. The screening/baseline stereo fundus photography and fundus autofluorescence (FAF) should be repeated with the delayed angiography, to help interpretation by the reading centre. The delayed angiography, with accompanying photographs and FAF, should be sent to the reading centre (contact details via link given above). Images should be captured in both eyes.

## 8. Assessment of Efficacy

### 8.1 Primary Efficacy Outcome

The primary outcome is the proportion of participants with a BCVA gain ≥10 ETDRS letters in the study eye at the 12 month visit.

This outcome enables future patients, post-trial, to weigh the likelihood of a meaningful vision gain versus the downsides of surgery, as advocated by our patient

focus group. The European Medicines Agency reports that 10 letters is the minimum clinically relevant amount perceived by patients,[10] and is associated with a clinically significant gain in the National Eye Institute (NEI) Visual Function Questionnaire (VFQ-25) composite score in patients with macular disease.[11] It is greater than the 6.5 letter test-retest variability in eyes with AMD.[12]

A 12 month endpoint allows time for SMH to resolve, and emerging cataract to be treated.

### 8.2 Secondary Efficacy Outcomes

The following secondary outcomes will be reported, with respect to the study eye, in addition to the National Eye Institute 25-item visual function questionnaire (NEI VFQ-25).

- Vision gain ≥10 ETDRS letters (at the 6 month visit)
- Mean ETDRS BCVA (6 and 12 month)
- Radner maximum reading speed (6 and 12 months)
- Area of scotoma size using Humphrey Field Analyser 10-2 or equivalent (6 and 12 months)
- NEI VFQ-25 composite score (6 and 12 months)
- Presence or absence of subfoveal fibrosis and/or atrophy and area of fovea-involving fibrosis/atrophy assessed using multimodal imaging by an independent reading centre, combining spectral domain optical coherence tomography (SD-OCT), fundus autofluorescence (FAF) and stereo fundus photographs (month 12).

## 9. Assessment of Safety

### 9.1 Safety Parameters

Safety assessments include regular measurement of BCVA, clinical examination, IOP, and OCT imaging. The final visit includes multimodal imaging read in a masked fashion by an independent reading centre, to look for and quantify any structural damage. Participants will be reviewed at 1-2 month intervals throughout most of the study, with shorter intervals following surgery, and investigators will ask specifically if participants have experienced any new symptoms or AEs at each visit.

All AEs, Serious Adverse Events (SAEs) and Important Medical Events (IMEs) should be recorded on the source data worksheets) and uploaded onto the electronic case report forms (eCRFs). Additionally, SAEs and IMEs should be reported to the Sponsor as detailed below.

Reference safety information for Actilyse is available in section 4.8 of the Actilyse Summary of Product Characteristics dated May 2019 as approved. Reference safety

information for non-Actilyse alteplase is available in the respective monographs for each brand of alteplase. Causality should be determined by the site's attending clinical investigator, in discussion with the Principal and/or Chief Investigator if necessary.

## 9.2 Definitions

The Medicines for Human Use (Clinical Trials) Regulations 2004 and Amended Regulations 2006 gives the following definitions:

### 9.2.1 Adverse Event (AE)

Any untoward medical occurrence in a subject to whom a medicinal product has been administered including occurrences which are not necessarily caused by or related to that product.

### 9.2.2 Adverse Reaction (AR)

Any untoward and unintended response in a subject to an IMP which is related to any dose administered to that subject.

### 9.2.3 Unexpected Adverse Reaction (UAR)

An adverse reaction, the nature and severity of which is not consistent with the information about Actilyse set out in the relevant Summary of Product Characteristics (SmPC), or in the respective monograph for the specific brand of alteplase, in the event a non-Actilyse brand of alteplase is used.

### 9.2.4 Serious Adverse Event (SAE), Serious Adverse Reaction (SAR) or Suspected Unexpected Serious Adverse Reaction (SUSAR)

Any adverse event, adverse reaction or unexpected adverse reaction, respectively, that

- Results in death;
- Is life-threatening;
- Required hospitalisation or prolongation of existing hospitalisation;
- Results in persistent or significant disability or incapacity;
- Consists of a congenital anomaly or birth defect.

### 9.2.5 Important Medical Events (IME) & Pregnancy

Events that may not be immediately life-threatening or result in death or hospitalisation but may jeopardise the patient or may require intervention to prevent one of the other outcomes listed in the definition above should also be considered serious.
Although not a serious adverse event, any unplanned pregnancy will also be reported via the SAE reporting system.

### 9.2.6 Reporting Responsibilities and Timelines

The Co-Sponsors' responsibility for Pharmacovigilance (as defined in Regulation 5 of the Medicines for Human Use [Clinical Trials] Regulations 2004) is delegated to the King's Health Partners Clinical Trials Office (KHP-CTO).

Serious adverse events (SAEs) will be reported from the time of informed consent until the end of the study.

All SAEs, SARs and SUSARs (excepting those specified in this protocol as not requiring reporting) will be reported immediately (and certainly no later than 24hrs) by the Chief Investigator to the KHP-CTO for review in accordance with the current Pharmacovigilance Policy.
The SAE form is to be completed as soon as possible after the clinical trial staff have been made aware of the event and sent immediately to the KHPCTO
by email: jcto.pharmacovigilance@kcl.ac.uk

The KHP-CTO will report SUSARs to the regulatory authorities (the competent authorities of European Economic Area states) in which the trial is taking place.
The Chief Investigator or delegated Investigator will report to the relevant ethics committee. Reporting timelines are as follows:

- SUSARs which are fatal or life-threatening must be reported not later than 7 days after the Sponsor is first aware of the reaction. Any additional relevant information must be reported within a further 8 days.
- SUSARs that are not fatal or life-threatening must be reported within 15 days of the Sponsor first becoming aware of the reaction.
- The Chief Investigator and KHP-CTO (on behalf of the co-sponsors), will submit a Development Safety Update Report (DSUR) relating to this trial IMP, to the MHRA and REC annually.

**9.2.7 Adverse events that do not require reporting**

Loss of vision as a result of disease progression and other events that are primary or secondary outcome measures are not considered to be SAEs and should be reported in the normal way, on source data worksheets and corresponding eCRFs.

Adverse events occurring after successful enrolment should be reported, except for loss of vision, as that will be captured in the efficacy analysis. Events that are commensurate with, and typical of, exudative AMD (CNV, IPCV or RAP) need not be reported, such as macular fluid leakage, exudates, pigment epithelial detachment, macular hypo- or hyperpigmentation, geographic atrophy, small amounts of retinal haemorrhage, and RPE rips. However, if these changes are not thought to be explained by the underling disease, then they should be reported. Breakthrough vitreous haemorrhage is well described with SMH, but since it could also potentially be aggravated by TPA it should be reported as an AE or SAE, as appropriate.

### 9.3 Treatment Stopping Rules and Premature Trial Discontinuation

#### 9.3.1 TPA (Alteplase)

Since TPA is given as a single dose during surgery there are no treatment stopping rules. Repeat surgery and TPA are allowed for recurrent SMH, but only in the surgical arm and only if the eligibility criteria are still met.

#### 9.3.2 Stopping Aflibercept

Aflibercept (Eylea) treatment is not a trial intervention *per se*, but rather a standard of care administered to both arms of the study. Eylea treatment should be discontinued as described in the current SmPC, available at https://www.medicines.org.uk/emc/product/2879. Presently, the SmPC states:

"If visual and anatomic outcomes indicate that the patient is not benefiting from continued treatment, Eylea should be discontinued."

It is important to note that SMH may make it difficult to determine if the participant is responding to Eylea. In this setting Investigators should give participants the 'benefit of the doubt', and continue treatment unless they are absolutely certain that treatment is not helping. The reason is that apparently unresponsive vision may yet improve when the SMH resolves, and meanwhile it is important to control the underlying disease to maximise the final visual recovery. Eylea may also promote SMH clearance and reduce the risk of additional haemorrhage. Hence, we **strongly advise that all participants receive all scheduled doses of Eylea**, namely monthly for 3 doses then 2-monthy until month 12 inclusive.

#### 9.3.3 Trial Discontinuation

The trial may be prematurely discontinued by the Sponsor, Chief Investigator or Regulatory Authority on the basis of new safety information, or on the guidance/direction of the relevant Data Monitoring Committee, Research Ethics Committee, Trial Steering Committee, or Competent Authority.

If the trial is prematurely discontinued, active participants will be informed and no further participant data will be collected. The Competent Authority and Research Ethics Committee will be informed within 15 days of the early termination of the trial.

## 10. Statistics

### 10.1 Clinically Meaningful Difference, Public Patient Involvement (PPI) and Sample Size

To calculate our sample size we undertook a bracketing exercise with our patient focus group. We asked them what improvement in 'treatment success', defined as

meaningful gain in vision, they would require to undergo eye surgery, with the attendant downsides such as discomfort, head positioning, complications, recovery, and possible cataract surgery, and assuming a 1 in 4 chance of success with anti-VEGF injections alone.[7]

The findings ranged from one patient who would not want surgery ‘at my age’, regardless of outcome, to another who would have surgery even if it improved his success from only 25% to 26%. The most common response was that patients wanted at least a 50% chance of success to consider vitrectomy.

What also emerged in this elderly group was a consensus that they wanted the doctor to make the best decision on their behalf. Paradoxically then, our public patient involvement (PPI) exercise led us to ask 10 Ophthalmologists, from junior to senior, across a range of subspecialities, what they would do, ‘if it was their eye’. We asked them to assume they were older adults with ≈25% success with anti-VEGF therapy. The median ‘success rate’ (defined as a 10 letter gain) needed to consider surgery differed if it was their potentially better or worse seeing eye (47.5% vs 55% respectively), but overall the average/median/mode were 49%, 50% and 50% respectively. Thus we used 50% as the minimum success rate needed to justify surgery.

Our synthesis of the literature[7] found that 27% of patients receiving anti-VEGF monotherapy for AMD-related SMH gained 2 Snellen lines (≈10 letters).

A two group $\chi^2$ test with a 5% two-sided significance level has 90.62% power to detect a difference between a Group 1 proportion, $\pi1$, of 0.27 and a Group 2 proportion, $\pi2$ of 0.5 (odds ratio 2.704) when the sample size in each group is 94 (NQuery Advanced software v 8.2.1). With ≈12% attrition the sample size inflates to 210 participants.

## 10.2 Randomisation and stratification

A web based bespoke randomisation system will be created in collaboration with the trial analyst/s and the CI and maintained by the King’s Clinical Trials Unit (KCTU) for the duration of the project. It will be hosted on a dedicated server within KCL.

The KCTU will provide the Trial Managers with a Data Management Plan for the web based KCTU Randomisation System, once the system is made live and ready for use. That document will be filed in the Trial Master File.

The CI or delegate will request usernames and passwords from the KCTU. System access will be strictly restricted through user-specific passwords to the authorised research team members. It is a legal requirement that passwords to the randomisation system are not shared, and that only those authorised to access the system are allowed to do so. If new staff members join the study, a user-specific username and password must be requested via the CI or delegate (e.g Trial Manager) from the KCTU team and a request for access to be revoked must be requested when staff members leave the project. Study site staff experiencing issues with system access or functionality should contact the CI or delegate (e.g Trial Manager) in the first instance.

Randomisation will be 1:1 at the patient level using the method of minimisation, stratified by the following factors:

1. Study site
2. Lens status: phakic or pseudophakic
3. SMH size: fully within the retinal vascular arcades, or not
4. BCVA: ≥35 letters or not, equivalent to 6/60 and near to the median presenting BCVA in the TAPAS study (ClinicalTrials.gov identifier: NCT01835067)

## 10.3 Analysis

### 10.3.1 Primary Analysis

The primary analysis will be conducted by the trial statistician, following the intent-to-treat principle where all randomised participants are analysed in their allocated group, whether or not they receive their randomised treatment. Baseline characteristics will be summarised for the two treatment groups. Continuous data will be summarised using means and standard deviations for data that follow a normal distribution or medians and interquartile ranges. Binary data will be reported as frequencies and percentages.

The primary outcome is whether or not the participant gains at least 10 ETDRS letters in their study eye at the month 12 visit. This will be compared between treatment groups using logistic regression, which will provide an effect estimate (odds ratio) and compare the proportions gaining at least 10 letters after adjustment for randomisation stratifiers. We will also report the difference in proportions of participants with an improvement in BCVA score of 10 letters or more with a two-sided confidence interval.

Secondary continuous outcomes measured at randomisation and on more than one occasion during follow-up will be analysed using a linear mixed effect model. The value at baseline, treatment group, follow-up time, and the stratifying variables will be included as fixed effects. Model assumptions will be assessed, and a logarithmic transformation used if this improves normality of residuals. Secondary dichotomous outcomes will be examined using the same techniques as the primary analysis.

All statistical tests will use a 2-sided P value of 0.05 unless otherwise specified. All confidence intervals will be two sided and 95%. A detailed Statistical Analysis Plan will be finalised prior to data lock. This will be circulated for comment to the Trial Steering Committee (TSC) and Data Monitoring and Ethics Committee (DMEC) for comment.

### 10.3.2 Safety

AEs and SAEs will be described using Medical Dictionary for Drug Regulatory Affairs (MedDRA) Preferred Terms.[13] The percentage of participants and study eyes in which AEs are observed will be reported within each group with 95 % confidence intervals.

Pre-specified Treatment Emergent AEs of special interest comprise cataract, retinal detachment, and arteriothrombotic events. The number of intraocular eye operations will be recorded for both groups.

### 10.3.3 Missing Data

Every effort will be made to avoid missing data, however we acknowledge that some is inevitable. How it is dealt with will depend on what is missing. We will report missingness wherever present. Reasons for missingness may be important and these will be investigated using logistic regression of covariates on an indicator of missingness.

Sensitivity analysis will investigate the validity of the missing data completely at random assumption and will explore imputation for missing data. In relation to the primary outcome variable we will conduct an available case analysis, but will then conduct a worst-case best-case analysis to examine the impact of missing data. The sensitivity analysis will consider participants in the surgery group with missing outcomes having a meaningful change in BCVA and participants in the anti-VEGF monotherapy control group with missing data not having a meaningful change in BCVA, and then the opposite. Our missing data analysis is complete if the results show that they are consistent with the available case analysis. If not, a range of more plausible assumptions will be explored following principles laid out in Carpenter & Kenwood.[14]

We will examine patterns of missingness and the reasons which caused the data to be missing. This will be achieved by examining the observed data and reasons for withdrawal in discussion with the clinical investigators. We will use this information to derive a series of missing data models. We will use these models to impute values in order to undertake a sensitivity analysis of the treatment effect estimate. If data are thought to be missing at random (MAR), conditional on additional variables not included in the primary analysis model, then the treatment effect will be estimated conditioning on the identified variable for example: conditioned on BCVA or size of haemorrhage at baseline. The scenario of missing not at random scenarios (MNAR) will be explored using a range of plausible assumptions and viewed graphically using a mean score approach via the rctmiss procedure in stata [http://www.mrc-bsu.cam.ac.uk/software/stata-software]. The impact of missing data will be mitigated against by incorporating information from earlier timepoints using the mixed model approach.

### 10.3.4 Subgroup Analyses

A prespecified subgroup analyses will assess the possible interactions between treatment and each of the following parameters:

- *Lens status (phakic vs pseudophakic):* Removal of pre-existing cataract may improve vision, whereas post-vitrectomy lens opacity that does not trigger cataract surgery in the 12 months' follow-up period may reduce vision. This may impact

on our analysis of the effect of surgery. It is much less problematic if we aim for a pragmatic trial design, wherein routine cataract management forms part of the "real world" patient pathway. However, we realise some clinicians and reviewers will want to isolate the effects of vitrectomy from lens events, and for them an analysis of pseudophakic eyes will provide a useful mechanistic insight.

- *SMH size (fully within the retinal vascular arcades, or not):* Many clinicians believe that large SMHs do better with surgery, as vitrectomy, TPA and gas is expected to provide more complete and rapid removal of blood from the fovea. We have chosen this size as it is a pragmatic and clinically useful size differentiator. Outcome has previously been shown to be closely related to the size of the haemorrhage. A SMH of less than 30 $mm^2$ (approximately just up to the arcades) has been shown to be predictive of an outcome of 6/60 or better in cases treated with vitrectomy and subretinal TPA.[15]
- *Lesion type (choroidal neovascularisation vs idiopathic polypoidal choroidal vasculopathy):* Some clinicians consider IPCV as part of the AMD spectrum, others consider it to be a distinct entity. Therefore, we cannot assume the effects of surgery will be the same in those with/without IPCV. IPCV is an important cause of SMH and is over-represented in case series of AMD with SMH. It is more common in Black and Asian patients. Retrospective studies report that SMH due to CNV has a worse outcome than SMH due to IPCV.[16] IPCV is often associated with blood below the RPE, and this space is not accessed via a sub-neuroretinal injection. Therefore, it is possible that surgery may be less effective for IPCV than CNV.
- *Duration of SMH (≤7 days vs >7 days):* It is possible that the benefits of surgery may diminish with a longer duration SMH, if the sustained toxic effects of blood reduce the impact of blood removal, yet the surgical risks remain constant. We chose 7 days as numerous studies have suggested that SMH duration of less than 7 days is associated with an improved visual outcome in surgically treated cases. These studies have typically been retrospective and the time point chosen arbitrarily, based on previous publications and animal studies. Regardless, it is an easy timepoint for clinicians to use and remember if it is found to be important.

These factors will be explored by adding interaction terms to the regression model for the primary outcome.

#### 10.3.5 Interim Analysis

No formal interim analysis or prespecified trial stopping rules are planned, but reports concerning participant safety and key outcomes will be reviewed at least yearly by the DMEC. If safety concerns are identified the DMEC can meet more frequently, and on an *ad hoc* urgent basis. If necessary for urgent safety reasons the Sponsor may stop or pause the trial immediately, without DMEC review.

#### 10.3.6 Pharmacokinetics and pharmacodynamics

Neither are planned, as TPA is extremely short-lived (systemic half-life 5 minutes) and intraocular sampling is not clinically justified. The pharmacokinetic and

pharmacodymanic profile of Eylea is well characterised and Eylea is anyway not considered an IMP within TIGER.

### 10.3.7 Measures to Minimise Bias

Concealment of randomized allocations until participants are enrolled and assigned to interventions will prevent bias from inadequate randomization. It is not possible to mask participants to their allocation because there is no sham for vitrectomy, and intravitreal gas is easily visible to participants. However, the BCVA primary outcome (BCVA at 12 months) will be assessed by masked observers using an established protocol, encouraging participants to "try their hardest", to minimise any differences in decision criterion and measurement bias. Secondary BCVA assessments will also be undertaken by masked observers. Scotoma size will be measured by an automated algorithm and morphological outcomes by masked graders. Patient-reported outcomes (NEI-VFQ and EQ-5D-5L) will potentially be the outcome most at risk of bias due to the participant's knowledge of treatment allocation, but this will be minimised by trial information promoting equipoise between the two interventions. The aflibercept dosing regimen is mandated, rather than based on physician discretion. Bias due to missing outcome data will be minimised by regular contact with participants throughout the trial to reduce loss to follow up (TAPAS had only 4% loss to follow up). Reporting bias will be minimised by pre-specifying and publishing all planned outcomes in a peer reviewed journal, and having a pre-specified statistical analysis plan.

## 11. Image Analysis

The Network of Ophthalmic Reading Centres UK (NetwORC UK), a network of three Ophthalmic Image Reading Centres in the United Kingdom (Belfast, Moorfields Eye Hospital in London, and Liverpool), will be responsible for masked image analysis (https://www.networcuk.com/). NetwORC UK will provide all training materials for image acquisition and will support sites throughout the trial. Image submission will be via a safe online submission system. All graders involved in TIGER are trained and certified for grading AMD at clinical trials level and will have passed their study specific certification for TIGER before grading commences.

For TIGER, there will be multimodal grading performed using all imaging modalities to enable the grader to provide a grade for relevant AMD-related abnormalities such as haemorrhage, atrophy and fibrosis in the study eye, and an overall AMD-phenotype decision in the study and fellow eye. All data will be entered into the TIGER database by certified NetwORC UK personnel. Quality assurance and quality control will be conducted according to NetwORC UK protocols and will be reported on to the study team and in the final report.

## 12. Health Economics Analysis

### 12.1 Economic research question

How cost effective is vitrectomy, subretinal TPA and intravitreal gas for SMH secondary to exudative AMD as compared with usual care (intravitreal 2 mg aflibercept monthly for three doses, then 2-monthly until month 12, given to both the surgical and control arms).

### 12. 2 Health economic evaluation

Untreated, SMH typically has a very severe impact on vision, but it is anticipated that surgery plus aflibercept will improve the outcome. However, it is not known if surgery plus aflibercept will improve vision more than aflibercept alone, and if they do, if the added costs and complications of surgery will offset that added benefit.

The primary outcome of this trial is a gain of at least 10 ETDRS letters of best-corrected visual acuity (BCVA). Visual acuity is a clinically accepted outcome measure that influences quality of life, and 10 letters exceeds the minimum clinically important difference. For these reasons the primary economic analysis is the incremental cost of surgery plus aflibercept achieving a 10 letter gain in BCVA, as compared with aflibercept monotherapy.

The initial analysis will take a UK perspective, but data will be made available to enable similar analyses in other European countries, subject to funding and relevant approvals. Firstly, from an NHS perspective we will compare treatment costs and service use with BCVA at 12 months, accounting for the need of further intervention. We will calculate QALYs at 12 months. We will use STATA to undertake a cost utility analysis. We will undertake a complete case analysis for the in-trial period of 12 months. In addition, we will use multiple imputation using chain equation methods for missing health utility data in a secondary analysis.

A cost-utility analysis using EQ-5D-5L (with the vision bolt on) questionnaire will generate mean cost per QALY estimates, using the area under the curve method, and bootstrapped confidence intervals (5000 replications). We will produce cost effectiveness planes and cost effectiveness acceptability curves. We will undertake sensitivity analyses to explore how sensitive results are to any assumptions in our analysis.

*Measuring intervention costs:* We will use national unit costs 2023 and make use of any relevant costing analysis in the literature to cost treatment paths of the two trial groups. Service use information will be collected as part of the eCRF at each data collection point. We will make use of the DIRUM database in the design of this instrument.

*EQ-5D-5L with vision bolt on question:* To calculate QALYs participants will complete the EQ-5D-5L with vision bolt on question at baseline, month 6 and month 12. We chose this version with 5 levels anticipating that it may be more sensitive than the 5L version in this population, potentially avoiding ceiling effects. EQ-5D-5L is a validated generic, health-related, preference-based measure comprising five domains: mobility; self-care; usual activities; pain and discomfort; anxiety and depression. Each domain has five levels. The questions are complemented by a visual analogue scale, with 0 representing the worst imaginable health and 100 representing the best imaginable health.

We have also included the Warwick-Edinburgh Mental Wellbeing scale short form 7-items questionnaire to assess the well-being of patients.

We will adhere to CHEERS standards for the reporting of economic evaluation studies.

*Economic budget impact model:* An economic budget impact model will be developed to determine the budget implications and any expected cost savings. Guided by good practice recommendations (ISPOR 2012), usually used with the introduction of new pharmaceutical products, these guidelines are equally useful for the development of a budget impact model for the use of surgery to treat SMH. We will model the budget impact. We will undertake sensitivity analysis, varying relevant assumptions i.e. alternative scenarios. We will validate the model in terms of face validity with ophthalmic surgeons. We will populate the model with data from the TIGER Trial and the literature. We will use a budget impact cost calculator approach (ISPOR 2012).

## 13. Patient and Public Involvement

### 13.1 Patient Involvement Group

A patient involvement group was formed on the 9th of October 2018 comprised of 3 patients who had been enrolled in the preceding pilot study (TAPAS; ClinicalTrials.gov Identifier: 01835067) and 2 patients who had the same treatment but outside of a study. This group was consulted to inform the study sample size as described in section 10.1. This involvement group will also provide feedback on final English versions of patient-facing documents, specifically the Patient Information Sheet (PIS), Informed Consent Form (ICF) and TIGER study website. We will also enquire for and incorporate qualitative feedback on how to improve recruitment and retention rates.

### 13.2 Study Patient Feedback Group

An additional 2-3 patients enrolled on the TIGER study will be invited to provide prospective feedback on their experiences of being in the study itself. There will be 2 primary activities asked of them during the study:

1. To provide feedback on study enrolment and activities using pre-defined questionnaires at the following time points:
   - Month 1 visit
   - Month 6 visit
   - Month 12 visit
2. To provide feedback on the study website (www.tigerstudy.org.uk) using a pre-defined questionnaire

### 13.3 Macular Society Involvement

The Macular Society was involved in reviewing the original study bid and will be asked to review the final English version of patient-facing documents. In addition, the Macular Society will raise awareness among potential study recruits (including potentially listing the TIGER study on their website), and will be involved in final discussions regarding study result interpretation and dissemination. A member of the Macular Society will sit on the TSC.

### 13.4 Patient Involvement Adviser and INVOLVE Input

A patient involvement adviser has been involved in the development of the bid and patient-involvement aspects of the study protocol, and will continue to advise and support in the delivery and conduct of patient-involvement activities during the study and final study results dissemination.

### 13.5 Study Results Dissemination

Following preliminary analysis, we will invite our patient involvement group, patient involvement adviser, Macular Society partners and at least 1 patient recruited on the TIGER study to review the primary results analysis. From this we will develop plans to produce a lay report and methods of disseminating study results.

## 14. Trial Steering Committee

A Trial Steering Committee (TSC) will be formed to provide general oversight of the trial.

### Membership of TSC

TSC membership will include members of the trial team, and independent TSC members. Independent TSC members may not participate in the TIGER trial other than as members of the TSC. Independent members should comprise a voting majority. The Chair will be independent. The TSC membership will be designed to provide a breadth of relevant skills, such as vitreoretinal clinicians, statistician, lay representative, and Principal Investigators' (PIs) representative.

## 15. Data Monitoring and Ethics Committee

A Data Monitoring and Ethics Committee (DMEC) will be formed to oversee the safety of participants on the TIGER study.

**Roles and Responsibilities**

The DMEC will periodically evaluate safety data, and make consequent recommendations to the Trial Steering Committee (TSC). The TSC may accept, reject, or modify DMEC recommendations.

The Trial Statistician will ensure that all Adverse Events (AEs) and Serious Adverse Events (SAEs) are reported to the DMEC as part of a DMEC report. An appropriate proportion of data should have been monitored by the Sponsor's Clinical Research Associate, in accordance with the Monitoring Plan (https://khpcto.co.uk/SOPs/03_MonitoringSOP.php). The primary emphasis of the DMEC member's review of these events will be on safety, so as to inform the TSC of any specific safety concerns in a timely manner.

The recommendations of the DMEC to the TSC may include:

- Discontinuation of the study if it is concluded by majority vote that the study participants are exposed to an unacceptable risk.
- Permanently or temporarily halt enrollment into the study.
- Modification of the study protocol.
- Continue the study according to the protocol and any related amendments.

## 16. Access to Source Data and Documents

The Principal Investigators (PIs) at each participating site, and their team, shall facilitate trial-related monitoring, audits, REC review, and regulatory inspections (where appropriate) by providing the Sponsor or their representative (eg King's Health Partner's Clinical Trials Office), Regulators and REC direct access to source data and other relevant documents e.g. source data worksheets, clinic and surgical notes, OCT and angiography images etc. Inspection and monitoring may take place remotely or at the recruiting site. For site visits the Sponsor's representative must have easy access to the relevant documents and desk-space to review them.

## 17. Ethics & Regulatory Approvals

The trial will be conducted in accordance with the principles of the Declaration of Helsinki (1996), Good Clinical Practice (GCP), and all applicable national regulatory requirements, for example, in the UK this would include, but not be limited to, the

Research Governance Framework and the Medicines for Human Use (Clinical Trial) Regulations 2004, as amended in 2006 and any subsequent amendments.
This protocol and related documents will be submitted for review to Cambridge East Research Ethics Committee (REC), and to the relevant Competent Authorities of participating countries.

The Chief Investigator will submit a final report at conclusion of the trial to the KHP-CTO (on behalf of the Sponsor), the REC and the Competent Authorities within the timelines defined in the relevant national regulations.

## 18. Quality Assurance

Monitoring of this trial will be to ensure compliance with Good Clinical Practice and scientific integrity will be managed and oversight retained by the KHP-CTO Quality Team.

## 19. Data Handling

The Chief Investigator will act as custodian for the trial data. Patient data will be pseudo-anonymised. All pseudo-anonymised data will be stored on a password protected computer. All trial data will be stored in line with the Medicines for Human Use (Clinical Trials) Amended Regulations 2006 and the Data Protection Act and archived in line with the Medicines for Human Use (Clinical Trials) Amended Regulations 2006, as defined in the King's Health Partners Clinical Trials Office Archiving Standard Operating Procedures.

## 20. Data Management

Sites will be provided with paper source data worksheets, for use alongside clinical visits. There will be a corresponding, online, secure, electronic case report form (eCRF). Sites will transfer data from the trial source data worksheets to the eCRF. The data will reside on an online, secure, trial database; Elsevier MACRO electronic data capture system (http://www.ctu.co.uk/).

A web based electronic data capture (EDC) system will be designed, using the InferMed MACRO 4 system. The EDC will be created in collaboration with the trial analyst/s and the CI and maintained by the King's Clinical Trials Unit (KCTU) for the duration of the project. It will be hosted on a dedicated server within KCL. In addition to the main trial database, a secondary MACRO database will be created to host the data from the Independent Reading Centre (IRC). The IRC staff will have access to this secondary database to enter their reading data, but only a limited number of unmasked IRC staff will have access to the main database, to help track participant withdrawals and image collection.

At the database design stage, validations will be programmed into the system to minimise data entry errors by querying the data entered in real time with sites.

The CI or delegate will request usernames and passwords from the KCTU. Database access will be strictly restricted through user-specific passwords to the authorised research team members. It is a legal requirement that passwords to the EDC are not shared, and that only those authorised to access the system are allowed to do so. If new staff members join the study, a user-specific username and password must be requested via the CI or delegate (e.g Trial Manager) from the KCTU team and a request for access to be revoked must be requested when staff members leave the project. Study site staff experiencing issues with system access or functionality should contact the CI or delegate (e.g Trial Manager) in the first instance.

At the end of the trial, the site PI will review all the data for each participant and provide electronic sign-off to verify that all the data are complete and correct.

Upon request, KCTU will provide a copy of the final exported dataset to the CI in .csv format. A copy of the full raw dataset is to be stored in the TMF.

## 21. Publication Policy

It is intended that the protocol for this study is published in a peer reviewed journal, to reduce the risk or perception of publication bias.

The main outcomes will be reported and disseminated at international conferences and submitted for publication in peer-reviewed scientific journals. Authorship will be determined by the Chief Investigator, to reflect the relative contribution of staff to the design, execution, analysis and write-up of the results. It is anticipated that Principal Investigators who recruited successfully to the trial will form part a TIGER study group, and that publication will be on behalf of the TIGER study group, but this will depend in part on the publication policy of the target journal. Principal Investigators who have made an especially large contribution to recruitment may be invited to take on a more prominent role in terms of authorship.

## 22. Insurance / Indemnity

The lead Sponsor, King's College London, will take primary responsibility for ensuring that the design of the study meets appropriate standards and that arrangements are in place to ensure appropriate conduct and reporting. King's College London also provides cover under it's No Fault Compensation Insurance, which provides for payment of damages or compensation in respect of any claim made by a research subject for bodily injury arising out of participation in a clinical trial or healthy volunteer study (with certain restrictions). The co-Sponsor, King's College Hospital, takes ultimate responsibility for arranging the initiation and management of this research, and will take responsibility for ensuring that appropriate standards, conduct and reporting are adhered to regarding its facilities and staff

involved with the project. King’s College Hospital will also undertake the governance review for the project and provide cover for clinical negligence by any of its staff in undertaking the research, under the CNST scheme managed by the NHS LA. King’s College London has acquired Clinical Trials Insurance from the Newline Syndicate 1218 at Lloyds, for sites outside the UK.

## 23. Financial Aspects

European Society of Retina Specialists (EURETINA) wished to support a clinical trial comparing vitrectomy TPA and gas with anti-VEGF monotherapy, for the treatment of SMH secondary to exudative AMD. They commissioned Fight for Sight to run a pan-European competition calling for bids to run the trial. Fight for Sight was tasked to run the competition, undertake the peer review and selection processes, and administer the grant.

The TIGER group won the competition and with it a grant to cover the research costs of the trial, including site payments for each patient recruited.

It is anticipated that many of the treatment costs will be commissioned through the usual clinical channels, as they form part of standard care. The distinction between research and standard clinical activity is shown in Section 7.1 (Schedule of Procedures by Visit Table), with research costs shown in red.

Although part of standard care, Bayer will provide Eylea free of charge to sites that require it. This allows TIGER to standardise anti-VEGF therapy across arms, sites, and countries.

TIGER will apply to be part of the UK’s National Institute for Health Research (NIHR) Clinical Research Network, and if admitted to their trial portfolio this will provide extra support for UK sites.

EURETINA also provided a small award directly to King’s College London, to establish the feasibility of running TIGER following Brexit.

The Sponsor (King’s College London with King’s College Hospital as NHS Co-Sponsor) will fund the NetwORC UK Reading Centre to provide an independent analysis of the ocular imaging.

## 24. References


1. Quartilho A, Simkiss P, Zekite A, Xing W, Wormald R, Bunce C. Leading causes of certifiable visual loss in England and Wales during the year ending 31 March 2013. *Eye.* 2016;30(4):602-607.

2. Al-Hity A, Steel DH, Yorston D, et al. Incidence of submacular haemorrhage (SMH) in Scotland: a Scottish Ophthalmic Surveillance Unit Study (SOSU) study. *Eye.* 2018; 33(3):486-491.
3. McGowan G, Steel DH, Yorston D. AMD with submacular hemorrhage: new insights from a population-based study. *Investigative ophthalmology & visual science.* 2014;55(13).
4. CommissionE. Rare diseaseshttp://ec.europa.eu/research/health/index.cfm?pg=area&areaname=rare. Accessed 13.10.2018.
5. Scupola A, Coscas G, Soubrane G, Balestrazzi E. Natural history of macular subretinal hemorrhage in age-related macular degeneration. *Ophthalmologica Journal international d'ophtalmologie International journal of ophthalmology Zeitschrift fur Augenheilkunde.* 1999;213(2):97-102.
6. Bressler NM, Bressler SB, Childs AL, et al. Surgery for hemorrhagic choroidal neovascular lesions of age-related macular degeneration: ophthalmic findings: SST report no. 13. *Ophthalmology.* 2004;111(11):1993-2006.
7. Stanescu-Segall D, Balta F, Jackson TL. Submacular hemorrhage in neovascular age-related macular degeneration: A synthesis of the literature. *Survey of ophthalmology.* 2016;61(1):18-32.
8. de Jong JH, van Zeeburg EJ, Cereda MG, et al. Intravitreal versus subretinal administration of recombinant tissue plasminogen activator combined with gas for acute submacular hemorrhages due to age-related macular degeneration: An Exploratory Prospective Study. *Retina.* 2016;36(5):914-925.
9. Heier JS, Brown DM, Chong V, et al. Intravitreal Aflibercept (VEGF Trap-Eye) in Wet Age-related Macular Degeneration. *Ophthalmology.* 2012;119(12):2537-2548.
10. Mosely JEMA. Visual Function Endpoints: The Regulatory Perspective. European Medicines Agency. https://www.ema.europa.eu/documents/presentation/presentation-day-1-visual-function-endpoints-regulatory-perspective_en.pdf. Published 2011. Accessed 7.10.18.

11. Chang TS, Bressler NM, Fine JT, et al. Improved vision-related function after ranibizumab treatment of neovascular age-related macular degeneration: results of a randomized clinical trial. *Archives of ophthalmology.* 2007;125(11):1460-1469.
12. Bokinni Y, Shah N, Maguire O, Laidlaw DA. Performance of a computerised visual acuity measurement device in subjects with age-related macular degeneration: comparison with gold standard ETDRS chart measurements. *Eye.* 2015;29(8):1085-1091.
13. Brown EG, Wood L, Wood S. The medical dictionary for regulatory activities (MedDRA). *Drug safety.* 1999;20(2):109-117.
14. Carpenter J, Kenward M. Missing data in randomised controlled trials: a practical guide. In. Birmingham: London School of Hygiene and Tropical Medicine; 2007:199.
15. Gonzalez-Lopez JJ, McGowan G, Chapman E, Yorston D. Vitrectomy with subretinal tissue plasminogen activator and ranibizumab for submacular haemorrhages secondary to age-related macular degeneration: retrospective case series of 45 consecutive cases. *Eye.* 2016;30(7):929-935.
16. Kunavisarut P, Thithuan T, Patikulsila D, et al. Submacular Hemorrhage: Visual Outcomes and Prognostic Factors. *Asia Pac J Ophthalmol (Phila).* 2018;7(2):109-113.
17. Stifter E, Konig F, Lang T, et al. Reliability of a standardized reading chart system: variance component analysis, test-retest and inter-chart reliability. *Graefe's archive for clinical and experimental ophthalmology = Albrecht von Graefes Archiv fur klinische und experimentelle Ophthalmologie.* 2004;242(1):31-39.
18. Bailey IL, Lovie JE. New design principles for visual acuity letter charts. *Am J Optom Physiol Opt.* 1976;53(11):740-745.
19. Chew EY, Kim J, Sperduto RD, et al. Evaluation of the age-related eye disease study clinical lens grading system AREDS report No. 31. *Ophthalmology.* 2010;117(11):2112-2119 e2113.

## 25.Signatures

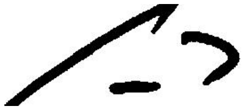

**06/12/2021**

Chief Investigator
*Tim Jackson*

Date

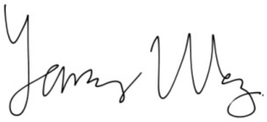

**06/12/2021**

Statistician
*Yanzhong Wang*

Date

# 26. Appendix A: Testing of Distance Visual Acuity and Radner Reading Speed

Study eye distance best-corrected visual acuity (BCVA) at baseline, month 6 and month 12 form the basis of the primary outcome and key secondary outcomes. Reading speed is an another important, patient-centred secondary outcome.

There are two types of ETDRS distance VA testing, Full Refracted BCVA and Clinic ETDRS VA.

Participants require a fully refracted BCVA at baseline, month 6 and 12. Radner reading speed is also completed at baseline, month 6 and 12. For the other visits a Clinic ETDRS VA is measured.

The schedule of BCVA and reading vision assessments is shown in the table below. Measurements are only required from the study eye, except for the fully refracted BCVA at baseline and month 12, and reading vision at month 12, when they should be measured in both eyes.

| **Visit** | Screening/ Baseline | D1* | W1* | M1* | M2 | M4 | M6 | M8 | M10 | M12 |
|---|---|---|---|---|---|---|---|---|---|---|
| Clinic ETDRS VA in study eye | | Arm A | Arm A | X | X | X | | X | X | |
| Full refracted ETDRS BCVA | Both eyes | | | | | | Study eye only | | | Both eyes |
| Radner reading vision | Study eye only | | | | | | Study eye only | | | Study eye only |

D1=day 1; W1=week 1; M2=month 2; *Masked assessors should not test VA for Arm A patients at D1, W1 and M1 visits.

Visual acuity testing and reading vision will be undertaken by **masked assessors**, who are unaware of the participant's treatment arm. They should not have access to prior VA measurements but can review prior refractions. The masked assessors should not test VA at the day 1, week 1 and month 1 visits, as this will alert them to participants having undergone surgery. Staff involved in TIGER should instruct participants not to reveal to VA assessors which arm of the trial they are in. Vision testing should occur prior to any intravitreal injections or surgery that day.

## 26.1 Clinic ETDRS Distance Visual Acuity

Clinic ETDRS VA should be undertaken in the study eye only, using an ETDRS chart with correction of any refractive error, with and without pinhole. Ideally, testing of Clinic ETDRS uses the 4 m chart, but a 2 m chart can be used if required. The lead VA examiner for the site should determine if refractive correction is undertaken with the participant's

most recent trial refraction using a trial frame, updated if needed, or the participant's regular distance spectacles, based on the particulars of their participant's visual function, spectacles and refractive error, and staff availability and skill mix. The site's lead VA examiner can delegate Clinic ETDRS VA testing to suitably trained clinic staff who, unlike those testing the Full Refracted ETDRS BCVA, need not be certified, but these staff members must appear on the delegation log and require at least GCP 'light' training.

### 26.2 Full Refracted ETDRS Best-corrected Distance Visual Acuity

Trial BCVA measurements (Baseline, Month 6 and Month 12) will be performed by trial certified vision examiners, in trial certified rooms, and with trial certified equipment. The **name** of the vision examiner should be documented in the participant's source data worksheets at each visit (not required on the eCRF). BCVA examiners are "masked" to trial assignment and previous BCVA testing results. Therefore, BCVA examiners should not have access to the participant's chart or previous BCVA testing results. Only the previous refraction should be made available. Refraction should be rechecked, and updated if necessary, prior to VA testing at Baseline, Month 6 and Month 12.

For BCVA measurement at intervening visits, see notes above.

**Equipment**
Refraction equipment required includes:

1. Retroilluminated Light box and ETDRS 4 original series Chart 1, Chart 2 and Chart R meter distance acuity chart set
2. Trial lens frames
3. Trial lens set with plus or minus cylinder lenses
4. Jackson cross-cylinders of 0.25, 0.50, and 1.00 diopters
5. Pinhole occluder
6. Tissues or eye pads and tape
7. A 1 meter rigid measuring stick
8. Standard Light Meter

***Visual Acuity Charts***

Chart 1 is used for testing the VA of the RIGHT eye; Chart 2 for testing the LEFT eye; and Chart R (or 3) for refraction only. Patients should not be allowed to see any of the charts before the examination.

***Visual Acuity Lane and Visual Acuity Box***

Cheap illumination light meters are readily available online. We recommend avoiding downloadable smartphone software app light meters as these have greater variability in readings. A distance of **4 metres** is required between the patient's eyes and the VA chart. With the box light off, not more than **15 foot-candles of light** (161.4 Lux) should fall on the centre of the chart. To measure the amount of light, the room is set up for VA testing,

but with the box light off. The light metre is placed at the fourth line from the top of the chart, with its back against the chart and the reading is taken. If more than one lane is available for testing VA, the VA of an individual patient should be measured in the same lane at each visit, if possible. If different lanes are used to test VA, they must each meet the same standards.

Retro illuminated ETDRS charts are used in this trial. The illuminator box will be either wall-mounted or mounted on a stand. The light box should be mounted at a height such that top of third row letter is 49 ± 2 inches from floor.

The VA light box is equipped with two General Electric 20-watt fluorescent tubes (or equivalent lightbox housing 24 Watt fluorescent tubes) and ballast. Each tube is partly covered by a fenestrated sleeve as supplied by the manufacturer, which is centered on the tube and open in the back. This serves as a "baffle" to produce even illumination over the testing chart. Because the illumination of fluorescent tubes diminishes by 5 percent during the first 100 hours and by another 5 percent during the next 2000 hours, new tubes should be kept on for a total time period of 4 days (96 hours) before use in the study, and should be replaced once a year. A minimum standard luminance of 85 cd/$m^2$ (80-160 cd/$m^2$) should be produced by the light box, which can be confirmed by checking with original manufacturer specifications. To confirm, we recommend the following minimum baseline illuminance (Lux) readings measured with a standard light meter and the chart switched on and room lights off: 250 Lux when positioned directly on the ETDRS screen, 90 Lux at 50 centimeters away, and 40 Lux at 1 meter.

A **light box log sheet** should be placed on the back of the light box, indicating the date on which the present tubes were installed. A spare set of burned in bulbs should be available on site and the study research coordinator should ensure annual replacement of the bulbs.

***Beginning Approximate Refraction***

At the Baseline visit, the patient's beginning refraction is determined by one of the following ways:

If the patient's VA is 6/30 (20/100) or better and the patient does not require glasses for distance vision, then the beginning approximate refraction should be no lens correction or plano.

If the patient's VA is 6/30 (20/100) or better and the patient requires glasses for distance viewing, the glasses should be measured using a focimeter, and these measurements are used for the beginning refraction
If the patient's VA is less than 6/30 (20/100) with or without correction, then retinoscopy or autorefraction should be performed to determine the beginning approximate refraction.

If the patient wears contact lenses for refraction, a notation should be made that the refraction was over contact lenses. It is suggested that the patient wear the contact lenses

for future examinations. If the patient is not a regular contact lens wearer and wore the lenses by mistake, they should be removed and you should wait at least 30 minutes before beginning the refraction. The patient should be reminded not to wear contact lenses at subsequent visits.

Refractions are performed with either plus or minus cylinder power. Whichever cylinder type is used at baseline (minus or plus) must be used for all subsequent visits. Best correction results should be recorded on the sponsor provided worksheet which will be included in the source data worksheets. **At each follow-up visit, the results of the protocol refraction from the previous visit are used as the beginning approximate refraction**. If the previous refraction is not available for some reason, the procedure described immediately above should be used. Whilst previous refraction results are made available at subsequent visits, **previous VA results should not be visible to the examiners at subsequent testing, so that *assessment of VA is masked to prior visual function (and to treatment arm)***.

The charts used for measuring distance VA must NOT be used for refraction. Refraction for each eye should be performed at 4 metres unless the patient's VA measured at **4 metres** on the refraction chart (Chart R or Chart 3) **is worse than 6/48 (20/160). If VA is worse than 6/48 (20/160) the eye is refracted at 1.0 metre.** If during the refraction process at one metre, the patient is reading letters on the eighth line or lower line of the chart, the refraction should continue at 4 metres. Whenever a patient cannot read any letters on the top line of Chart R or Chart 3 at 1.0 metre the vision should be checked with a pinhole to see whether reduced vision is due, at least in part, to a larger refractive error, however, be aware that submacular haemorrhage may produce a central scotoma that negates the refractive benefit of a pinhole.

***Patient Refraction***

Patient refraction allows one to determine the best correction for a patient to perform the VA tests. The "push plus" approach is used. Add minus dioptre spherical corrections only when the patient is able to read at least one more letter on a line or a letter on a smaller line.

Procedure

1. Measure and record the distance vision of the eye being tested using Chart R while occluding the fellow eye. The fellow eye should be lightly patched with an eye pad or tissue and tape. Patients should be reminded to blink and encouraged to use eccentric fixation, or their side vision, when necessary.

2. All refraction and vision testing must be done at 4 metres or 1 metre. Distance for 4 metres is 13 feet and 1.5 inches or 157.5 inches. The 1 metre distance is 39 and 3/8 inches.

3. All patients should be seated for testing. A rigid measuring device should be used to measure the distance from the patient to the chart if testing is done at 1 metre. The distance is measured from the outer canthus to the center of the second letter (left

eye) or fourth letter (right eye) of the third line of the chart. For 4 metre testing, clear and permanent floor markings should be used to mark the distance for consistency.

4. Place and adjust the trial frame on the patient's face so that the lens cells are parallel to the anterior plane of the orbits and centered in front of the pupils. Adjust the lens cells for the proper distance from the cornea. Be sure the trial frame is comfortable on the patient's face.

5. Occlude the left eye by lightly patching with an eye pad or tissue and tape. Place the spherical lens correction in the compartment closest to the eye. The cylindrical lens correction, if present, is placed in the compartment in front of the spherical correction. Adjust the axis.

6. Spherical Correction: To determine the highest plus or least minus sphere, refract the right eye. The following refraction steps (6 – 10) are recommended for VAs of 6/3 (20/10) to 6/24 (20/80) with the beginning approximate refraction. For VAs less than 6/24 (20/80), refer to the refraction table for the appropriate sphere and cylinder powers and testing distance (see summary below) and follow a similar procedure. Note: Whenever VA is improved to a higher range, refraction should be performed with the smaller sphere and cylinder powers given for the better VA level (See table at end of appendix).
   a) Hold a +0.50 sphere in front of the patient's right eye. The patient should be looking at the smallest legible line on the VA chart. In these exact words, ask the patient, "Is this better, worse, or no change?"
   b) If the patient responds that the vision is worse or blurred, remove the +0.50 sphere from in front of the trial frame and go to Step 6d.
   c) If the patient responds better or no change, remove the +0.50 sphere from in front of the trial frame and replace the spherical lens in the trial frame with a spherical lens that is one-half dioptre more positive. Continue this procedure by returning to Step 6a and repeating this process until a +0.50 makes the vision worse or blurred and then go to Step 6d.
   d) Hold a -0.50 sphere in front of the patient's right eye. In these exact words, ask the patient, "Is this better, worse or no change?" If the patient replies "worse" or "no change", go to Step 6f. If they reply "better" go to step 6e.
   e) Hold the -0.50 sphere in front of the eye. If the patient responds that the vision is better, ask the patient to read the VA chart. Only when the VA is improved, by at least one letter, may you increase the minus by 0.50 (or decrease the plus) and repeat Step 6d. Whenever VA is not improved, go to Step 6f.
   f) Remove the -0.50 sphere from in front of the eye and hold a +0.50 sphere in front of the right eye. In these exact words, ask the patient, "Is this better, worse, or no change?" If the patient responds that vision is better or unchanged, then return to Step 6c. Otherwise, go to Step 7. Spherical testing should always end with a plus lens.

7. Cylinder Axis: To determine and refine the cylinder axis for PLUS cylinder, proceed as follows; (If minus cylinders are used, the appropriate technique using minus cylinders must be employed and minus cylinder must be used throughout the trial.)

a) Have the patient look at a line which is either one or two lines larger than the smallest line the patient is able to read. Ask the patient to focus on a rounded letter such as "C", "D", or "O". The patient should focus on this same letter throughout this procedure.
b) If a cylinder is present in the beginning approximate refraction, then go to Step 7c. Otherwise, follow the option listed below to determine if cylinder may be needed.

   Testing for cylinder when there is none in the beginning approximate refraction:
   Place a +0.50 dioptre cylinder with the positive axis first at 90°, then compare this to no cylinder; repeat this procedure for 180°, then 45°, and 135° always comparing to no cylinder after each axis position. If the patient says that vision is improved at any one of the four axis positions, place a +0.50 cylindrical lens in the trial frame at the preferred axis and go to step 7c. If the patient prefers no cylinder at all four axis positions, then go to Step 9.
c) Place the +0.25 dioptre hand held cross-cylinder (for VA 6/3 – 6/24; 20/10-20/80) first with the positive axis 45° to the right of the preferred cylinder axis (as determined above), and second with the positive axis 45° to the left of the preferred cylinder axis. Ask the patient, "Which do you like better, position one or position two?" Also, tell the patient that both positions may blur their vision. The patient must choose the least blurred position, either one or two. "Neither" is allowed only if both positions are equally blurred or equally good.
d) If "neither" position is better and this was the first test of axis position, move the axis of the cylinder in the trial frame 15° to the right or left and return to Step 7c. Otherwise, proceed to Step 7e.
e) When one position is preferred over another, move the cylinder to the preferred positive axis position in the step sizes noted below and return to Step 7c. If no single position is better than another than go to Step 8.

**Cylinder Refinement: *suggested* axis step sizes**

| Cylinder Power | Axis Step Sizes |
|---|---|
| <1.00D | 15° |
| 1.00 to <2.00D | 10 ° |
| 2.00 to <3.00D | 5 ° |
| 3.00 to<5.00D | 3 ° |
| 5.00 to<8.00D | 2 ° |

8. **Cylinder Power**: Cylinder power is refined by following the steps:
   a) Ask the patient to look at the **smallest line** that can be read on the VA chart.
   b) Test the cylinder power by placing the 0.25 dioptre cross-cylinder (for vision of 6/3 - 6/24; 20/10-20/80) first with the positive axis and second with the negative axis coincident with the cylinder axis. Ask the patient, "Which is better, position one or position two?" Do not give the patient the choice of neither.
   c) If the patient prefers the minus axis coincident with the cylinder axis, the total power of the correcting plus cylinder is reduced by 0.25 dioptre. Repeat the process until the patient cannot choose one of the cross cylinder positions over the other. If the patient indicates a change that would introduce negative cylinder power, remove all cylinder power and continue testing for positive cylinder power at an axis 90°away from the previous axis. Otherwise go to Step 8d.
   d) If the patient prefers the plus axis coincident with the cylinder axis, increase the power of the cylinder by 0.25 dioptres and return to Step 8b. Otherwise proceed to Step 8e.
   e) When the patient feels that both positions are equally bad or good, and the cylinder power in the trial frame has changed by more than 0.50 dioptre, return to Step 7c and re-check the axis if necessary. Otherwise, proceed to Step 9.
   *Note: If the cylinder is changed by more than 0.50 dioptre, the **spherical equivalent** should be maintained. (For each 0.50 **plus** CX increase, add –0.25 to the sphere, for each 0.50 **minus** CX increase, add +0.25 to the sphere).*

9. **Spherical Correction *Refinement***: Recheck, or ***"refine"*** the power of the sphere by adding **+0.25 and -0.25** spheres and changing the spherical power by 0.25 dioptre increments of the appropriate sign until the patient cannot detect any improvement in vision. As a reminder, **minus sphere should only be added if the patient can read additional letters** and spherical testing should always begin and end with a plus lens.

10. Record the lens corrections obtained by patient refraction for the right eye on the examination form in the section for VA measurements as the corrections obtained by protocol refraction for the right eye.

11. Repeat the entire process (Steps 1-10) for the left eye and record the refraction result on the VAE worksheet.

12. As noted above, bilateral fully refracted BCVA is only required at baseline and month 12. At month 6 (and other visits) only the study eye needs to be tested. Refraction should be updated at baseline, and months 6 and 12. For intervening ETDRS BCVA measurements use the previously determined refraction.

***Best-Corrected Visual Acuity Measures***

As a reminder, Charts 1, 2, and R (or 3) are used for testing the right eye, left eye, and refraction, respectively. Patients should not see the charts until the test begins. The lens

correction from the patient refraction should be in the trial frame worn by the patient. All eyes must be tested at 4 metres first, even if the refraction was performed at 1 metre.

The patient should be seated comfortably directly in front of the chart so that the eyes remain at the 4 metre distance. For bilateral testing, always begins with the right eye. The fellow should be occluded with a folded tissue or eye pad lightly taped over the eye behind the trial frame serves as an effective occluder that allows eccentric fixation without inadvertent use of the covered eye. After testing the right eye, occlusion of the right eye should be done BEFORE Chart 2 is put up for testing the left eye.

The patient is asked to read the letters slowly, approximately one letter per second. The patient should be told that only one chance is given to read each letter, but they may change their mind before moving to the next letter. If the patient is unsure about the identity of the letter, then the patient should be encouraged to guess.

The patient should begin by reading the top line of the chart and continue reading every letter on each smaller line, from left to right on each line. The patient should be encouraged to continue reading even if making mistakes. Each letter read is counted. The examiner circles every correct letter read and totals each line and the whole column (0 if no letters are correct) on the data collection form. An X is put through letters read incorrectly. Letters, for which no guess was attempted, are not marked. When a patient reaches a level where he/she cannot guess, the examiner may stop the test provided that the patient has made errors on previous guesses, which is a clear indication that the best VA has been obtained.

When a patient cannot read at least 20 letters on the chart at 4.0 metres, the patient is tested at 1.0 metre. The distance from the patient to the chart should be measured again using the rigid 1 metre stick. The distance is measured from the outer canthus to the center of the fourth letter (right eye) or the second letter (left eye) of the third line of the chart. The spherical correction in the trial frame should be changed by adding +0.75 to correct for the closer test distance. The patient may fixate eccentrically or turn or shake his/her head to improve VA. Particular care should be taken to make sure the patient does not move forward when testing at 1 metre. The patient should be reminded to blink.

The examiner should not tell the patient if a letter was identified correctly. The patient may be encouraged by neutral comments, such as “good”, “next”, and “OK”. The examiner should not stand close to the chart during testing. Attention should be focused on the patient and the data collection form. If the patient has difficulty locating the next line to read, the examiner may go up to the chart and point briefly to the next line to be read, but then must move away from the chart.

When 20 or more letters are read at 4 metres the VA score for that eye is recorded as the number of letters correct plus 30 (refer to the VA worksheet) The patient gets credit for the 30 1M letters even though they did not have to read them. Otherwise, the VA score is the number of letters read correctly at 1.0 metre plus the number, if any, read at 4M. If no letters are read correctly at either 4.0 metres or 1 metre, then the VA score is recorded as 0.

### *Testing for Count Fingers Vision, Hand Motion Vision and Light Perception/No Light Perception (NLP) Vision*

If the patient's VA is so poor that he/she cannot read any chart letters when tested at 1 metre then the patient's ability to count fingers, detect hand motion, or have light perception should be evaluated.

### *Testing for Count Fingers Vision*

In testing for count fingers vision, the examiner's hand holding 1, 2, or 5 fingers is held steady at a distance of two feet directly in front of the eye being examined. The fellow eye is completely occluded with a patch on the face. A light should be shown directly on the hand from behind the patient. The examiner's fingers should be presented in random order and repeated 5 times. Eccentric fixation, if present, should be encouraged. If the patient correctly identifies three of the five presentations, then count fingers vision is noted. If not, then the patient must be tested for hand motion vision.

### *Testing for Hand Motion Vision*

The examiner's hand with all fingers spread out should be extended two feet directly in front of the eye being examined. The fellow eye should be occluded with a patch on the patient's face. A light should be shone directly on the examiner's hand from behind the patient. The examiner's hand should be moved in an up-and-down direction (vertically) or in a side-to-side direction (horizontally) at a constant speed of approximately one back and forth presentation per second. The patient is instructed that the examiner's hand will be presented and they will have to respond to the question: "What am I doing with my hand?" This should be repeated five times. Three out of five correct responses indicate that hand motion vision is present. If the patient does not correctly identify three of five presentations, then you must test for light perception.

### *Testing for Light Perception/No Light Perception Vision*

Light perception should be tested with an indirect ophthalmoscope in a darkened room. The fellow eye should be completely patched and also covered by the patient's hand. The indirect ophthalmoscope light should be in focus at 1 metre with the rheostat set at maximum voltage. From that distance the beam should be directed in and out of the patient's eye at least four times, and the patient should be asked to respond when he or she sees the light. If the examiner is convinced that the patient perceives the light, vision should be recorded as "light perception", if not, vision should be recorded as "no light perception".

| 4M Refraction Protocol Summary | | | | | | | | |
|---|---|---|---|---|---|---|---|---|
| **Refraction Distance** | **Check Sphere First** | | **Check Cylinder Axis then Power** | | | **Sphere “Refinement”** | | |
| **If VA on “R” chart is between:** | **Power (a)** | **Increment** | **Axis (b)** | **Power (c)** | **Increment** | **Power (d)** | **Incremen t** |
| **6/3 -6/24<br>20/10 - 20/80<br>(4 m)** | **+.50<br>-.50** | **+.50<br>-.50** | **.25<br>JCC** | **.25<br>JCC** | **+.25<br>-.25** | **+.25<br>-.25** | **+.25<br>-.25** |
| **6/30 – 6/48<br>20/100 - 20/160<br>(4 m)** | **+1.00<br>-1.00** | **+1.00<br>-1.00** | **.50J CC** | **.50<br>JCC** | **+.50<br>-.50** | **+.50<br>-.50** | **+.50<br>-.50** |
| **6/60 -6/120<br>20/200 - 20/400<br>(1 m)** | **+2.00<br>-2.00** | **+2.00<br>-2.00** | **1.00<br>JCC** | **1.00<br>JCC** | **+1.00<br>-1.00** | **+1.00<br>-1.00** | **+1.00<br>-1.00** |
| **<6/120<br><20/400<br>(1.0 m)**<br>sequence refraction a-d | **+2.00<br>-2.00** | **+2.00<br>-2.00** | **No cylinder test required** | | | **No refinement required** | |

## 26.3 Radner Reading Chart

The "Radner Reading Charts" have been developed on the basis of the concept of "sentence optotypes" for the examination reading acuity and speed.[17] Print sizes are geometrically (logarithmically) scaled. Reading acuity is given in logRAD (logReading-Acuity-Determination) to permit statistical analysis, and the results obtained can be compared to other logarithmically scaled vision systems (e.g. logMAR).[18] Reading speed is analyzed in words per minute (wpm).

To guarantee accurate, reproducible and standardized measurements of reading speed and reading acuity, "sentence optotypes" have been created to minimize the variations between the test items. Through interdisciplinary cooperation, a series of test sentences were developed that are highly comparable in terms of the number of words (14 words), as well as the word length, number of syllables, position of words, lexical difficulty and syntactical complexity. The most similar sentences were statistically selected for the Radner Reading Charts. The Radner Reading Charts have then been statistically evaluated in terms of test-retest reliability, inter-chart reliability and a variance component analysis.[17]

### Aim

In TIGER, the Radner maximum reading speed is an important secondary outcome, but two additional measurements will be acquired alongside this:

- Reading acuity (unit, logRAD, in 0.1 log steps): Equivalent to logMAR distance acuity. Corresponds to the smallest sentence optotype read that does not meet one of the stop criteria (see Technique below).
- Maximum reading speed (words per minute): Fastest reading speed achievable at any sentence optotype that does not meet one of the stop criteria (see Technique below).
- Mean reading speed (words per minute): Average of the fastest 3 sentences optotypes read that do not meet one of the stop criteria, inclusive of all optotypes. If only 2 sentence optotypes were read correctly, document the average of these.

### Instructions

Testing should be undertaken in the study eye only, at baseline, Month 6 and Month 12. Test reading vision after the measurement of ETDRS distance BCVA and before visual field testing. Record timings with a digital stopwatch to 2 decimal places (i.e. 0.00 seconds)

### Near correction, test distance, and illumination

The refraction used for testing ETDRS vision at 4 m should be altered for reading vision testing, which is performed at 25 cm, by providing a 4.0 Dioptre ADD. Verify the distance regularly throughout testing, with a ruler.

At baseline record the level of illumination measured immediately in front of the middle of the reading chart (acceptable range: 100-110 cd/$m^2$ or 480-520 Lux), and ensure the same level of illumination and testing conditions are used for testing at Month 6 and 12. This level of illumination can be achieved with a standard desk lamp.

**Technique**

a) The reading chart is held by the patient or can be placed on a stand at 25 cm distance; the sentences are covered with a piece of paper.

b) Instruct the patient to uncover the chart sentence by sentence and to read only one sentence per measurement. Instruct the patient to uncover the next sentence only when asked to do so - the examiner gives the command to uncover the next sentence.

c) "Please read the sentences aloud as quickly and accurately as possible. Read each sentence to the end, and do not correct reading errors."

d) "Please uncover the first sentence and start reading." Start the measurement with the stopwatch when the patient starts reading, and measure the reading time until the end of the sentence. If possible, aim to improve accuracy by starting the stopwatch with the initial pre-movements of the lips at the vocal onset (pre-phonetic strain).

e) Write the reading time on the scoring sheet next to the sentence read, and record any reading errors by marking them on the sheet (sum up the syllables of words read wrong). If patient's accents alter the pronunciation of a word, but the meaning is correct (example: different => diff´rent), credit the patient with the full word, and the syllables are counted as given in the word books.

f) Stop criterion: reading time longer than 30 seconds for a sentence, inability to complete a sentence, or severe errors or omissions that distort the meaning and/or structure of a sentence as to render it unintelligible.

**Calculating Reading Speed**

Reading speed in words per minute (wpm) can be calculated on the basis of the number of words in a sentence (=14) and the time (t = seconds) needed to read the sentence, or using the values in Table 1.

Reading speed (wpm): 14/time in seconds x60 = 840/time in seconds

**Table 1:**
**Reading Speed:**

| sec. | w/min | sec. | w/min | sec. | w/min | sec. | w/min | sec. | w/min | sec. | w/min |
|---|---|---|---|---|---|---|---|---|---|---|---|
| 3.0 | 280 | 4.0 | 210 | 6.0 | 140 | 8.0 | 105 | 10.0 | 84 | 18.0 | 47 |
| 3.1 | 271 | 4.2 | 200 | 6.2 | 135 | 8.2 | 102 | 10.5 | 80 | 19.0 | 44 |
| 3.2 | 263 | 4.4 | 191 | 6.4 | 131 | 8.4 | 100 | 11.0 | 76 | 20.0 | 42 |
| 3.3 | 255 | 4.6 | 183 | 6.6 | 127 | 8.6 | 98 | 11.5 | 73 | 21.0 | 40 |
| 3.4 | 247 | 4.8 | 175 | 6.8 | 124 | 8.8 | 95 | 12.0 | 70 | 22.0 | 38 |
| 3.5 | 240 | 5.0 | 168 | 7.0 | 120 | 9.0 | 93 | 13.0 | 65 | 23.0 | 37 |
| 3.6 | 233 | 5.2 | 162 | 7.2 | 117 | 9.2 | 91 | 14.0 | 60 | 25.0 | 34 |
| 3.7 | 227 | 5.4 | 156 | 7.4 | 114 | 9.4 | 89 | 15.0 | 56 | 27.0 | 31 |
| 3.8 | 221 | 5.6 | 150 | 7.6 | 111 | 9.6 | 88 | 16.0 | 53 | 29.0 | 29 |
| 3.9 | 215 | 5.8 | 145 | 7.8 | 108 | 9.8 | 86 | 17.0 | 49 | 30.0 | 28 |

# 27.Appendix B: AREDS Lens Opacity Grading

Vitrectomy is known to cause cataract, and this may influence how we interpret visual acuity gains or losses in TIGER. For this reason, the trial mandates regular assessment of lens opacity using the widely adopted and validated Age-Related Eye Disease Study (AREDS) Clinical Lens Grading System.[19] This grades lens opacities against standard photographs, which are provided on the next page. Grading the lens may also serve as a prompt to remove developing cataract. Grade the lens opacity in phakic study eyes only, (left and right), as follows:

- Dilate pupils to at least 5 mm diameter
- Use slit lamp with ~10X magnification
- Use brightest beam intensity
- Nuclear opacity
- Orient beam at 45° to viewing axis
- Adjust slit beam to standard parameters: 8 mm height and 0.3 mm width
- Compare opalescence of nucleus with that in standard photos
- Cortical and PSC opacities
- Select wide slit beam setting optimum for retro-illumination of lens
- Visualize lens opacities against red fundus reflex background
- Count only opacities definitely visible against red reflex
- Mentally combine all cortical opacities into one contiguous area

- Compare total opacity area with that in standard photos
- Classify each opacity with scale defined by 3 standard photos
- Select nearest half-step which is:
    - Similar to standard or between two standards
    - Obviously less than mildest standard or greater than most severe

# AREDS 2008 Clinical Lens Opacity Standard Photographs

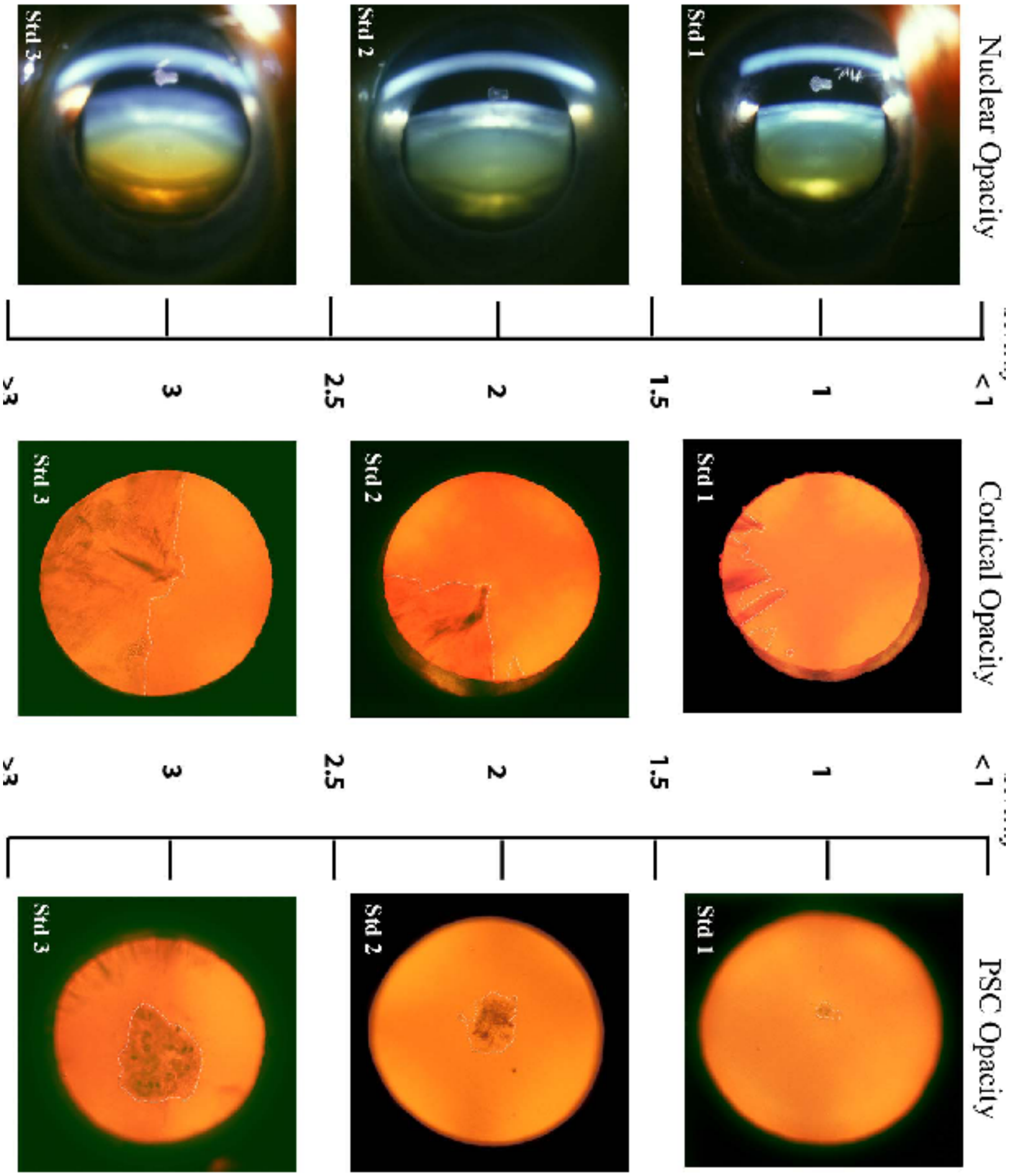

## 28.Appendix C: Visual Field Testing

Submacular haemorrhage (SMH) may lead to central macular scarring, and consequently a central scotoma in the affected eye. Hence, this study incorporates an assessment of the central visual field at screening, month 6 and month 12.

Central visual field testing should be completed in the study eye only, by experienced operators, using the Swedish Interactive Threshold Algorithm (SITA) standard 10-2 program, test stimulus size III, and the Zeiss Humphrey Field Analyser 3 (HFA3, Carl Zeiss Meditec, Dublin, USA). If this device is not available please use an equivalent programme from another device, but contact the study team for advice on its suitability beforehand. The same machine should be used for a given participant for each of their visual field tests. Visual field testing should occur before any intravitreal injections or surgery that day.

Visual field testing should occur after refraction and ETDRS visual acuity testing, to minimise the impact of any testing fatigue on the primary outcome (visual acuity). This will also provide a same-day distance refractive measurement, which can be used to determine the correct trial lens for visual field testing, as per the Humphrey Field Analyser manual (see sections B-1, B-2, 4-3 and 4-5), available here, or at:

https://www.zeiss.fr/content/dam/Meditec/international/ifu/documents/hfa3/current/2660021166131_a_artwork.pdf

Reliable visual field testing (<15% false positives and <20% fixation losses) may not be possible in some participants due to poor visual acuity and the operator should use the eye tracker to carefully monitor eye movements and prompt the participant to maintain central fixation as required, throughout the test.

Visual field tests should be scanned and saved as .pdfs. The file name should include the participant's study ID and visit (eg month 6), but not the participant's name.

The visual field pdfs should be transferred to the reading centre, as per the colour fundus photographs and angiography images. The address and contact details for the NetwORC UK Reading Centre are available here, or at:

https://www.networcuk.com/

# 29. Appendix D: List of abbreviations

| | |
|---|---|
| AE | Adverse Event |
| AR | Adverse Reaction |
| AREDS | Age-Related Eye Disease Study |
| AMD | Age-Related Macular Degeneration |
| VEGF | Anti-Vascular Endothelial Growth Factor |
| BCVA | Best-Corrected Visual Acuity |
| CI | Chief Investigator |
| CNV | Choroidal Neovascularisation |
| CTIMP | Clinical Trial of an Investigational Medicinal Project |
| ConMeds | Concomitant Medications |
| DMEC | Data Monitoring and Ethics Committee |
| ETDRS | Early Treat Diabetic Retinopathy Study |
| eCRF | Electronic Case Report Form |
| EDC | Electronic Data Capture |
| ERM | Epiretinal Membrane |
| EURETINA | European Society of Retina SpecialistS |
| FAF | Fundus Autofluorescence |
| GCP | Good Clinical Practice |
| HFA 10-2 VF | Humphrey 10-2 Visual Field |
| IPCV | Idiopathic Polypoidal Choroidal Vasculopathy |
| IME | Important Medical Events |
| ICG | Indocyanine Green (ICG) Angiography |
| ILM | Internal Limiting Membrane |
| INR | International Normalised Ratio |
| IOP | Intraocular Pressure |
| IUD | Intrauterine Device |
| KCTU | King's Clinical Trials Unit |
| KHPCTO | King's Health Partners Clinical Trials Office |
| LogRAD | Log Reading-Acuity-Determination |
| LogMAR | Logarithm of the Minimum Angle of Resolution |
| MedDRA | Medical Dictionary for Drug Regulatory Affairs |
| MAR | Missing At Random |
| MNAR | Missing Not At Random Scenarios |
| MI | Myocardial Infarction |
| NEI | National Eye Institute |
| VFQ-25 | Visual Function Questionnaire |
| NIMP | Non-Investigational Medicinal Product |
| PDT | Photodynamic Therapy |

| | |
|---|---|
| PI | Principal Investigator |
| PPI | Public Patient Involvement |
| PE | Pulmonary Embolus |
| RCT | Randomised Controlled Trial |
| REC | Research Ethics Committee |
| RAP | Retinal Angiomatous Proliferation |
| RPE | Retinal Pigment Epithelium |
| SOSU | Scottish Ophthalmic Surveillance Unit |
| SAE | Serious Adverse Event |
| SAR | Serious Adverse Reaction |
| SD-OCT | Spectral Domain Optical Coherence Tomography |
| EQ-5D-5L | EuroQol-5D Standardized Instrument for Measuring Generic Health Status with 5-item (5L) vision bolt-on |
| SMH | Submacular Haemorrhage |
| $SF_6$ | Sulfahexafluoride |
| SmPC | Summary of Medical Product Characteristics |
| SWEMWBS | Short Warwick-Edinburgh Mental Well-being Scale questionnaire |
| NetwORC | The Network of Ophthalmic Reading Centres UK |
| TPA | Tissue Plasminogen Activator |
| TSC | Trial Steering Committee |
| UAR | Unexpected Adverse Reaction |
| UK | United Kingdom |
| USAR | Unexpected Serious Adverse Reaction |
| WPM | Words per Minute |
