# Supplementary material for: Vitrectomy, subretinal Tissue plasminogen activator and Intravitreal Gas for submacular haemorrhage secondary to Exudative Age-Related macular degeneration (TIGER): update to study protocol and addition of a statistical analysis plan and health economic analysis plan for a randomised controlled surgical trial
Source: Trials. 2025 Apr 14;26:131. doi: 10.1186/s13063-025-08727-8 (PMC11995560; doi:10.1186/s13063-025-08727-8)
Supplement: Supplementary file 2 — Additional file 2: Appendix 2. Summary of Changes in TIGER Master Protocol version 2.0. [file 13063_2025_8727_MOESM2_ESM.docx]

**TIGER Master Protocol v2.0 List of Tracked Changes**

| **No** | **Section** | **Reason for Change** (additions/amendments in **BOLD + UNDERLINED**) | **Person enacting change** | **Date of Change** |
| --- | --- | --- | --- | --- |
| 1 | Section 12 “Health Economics Analysis” | Typo – “A” missing from “TPA”. Corrected. | Chan Ning Lee (CRF) | 18.05.2022 |
| 2 | Section 5.1 “Investigational Medicinal Product (IMP): Tissue Plasminogen Activator (TPA, Alteplase, Actilyse)” | - Addition of trademark symbols for Actilyse (®) - Amendments and additions to the text to clarify difference between use of licensed Actilyse^®^ brand alteplase and other unlicensed forms of alteplase. Reason for this is due to shortages of Actilyse^®^ across UK and EU causing sites to purchase unlicensed alteplase to keep stock available. The unlicensed forms of alteplase are identical in terms of excipients, indications and molecular structure, but are marketed under different brand names as part of commercial agreements between the respective owner companies (examples: Activase^®^, Genentech; Actilyse^®^, Beohringer-Ingelheim; Activacin^®^, Kyowa Hakko Kogyo; and GRTPA^®^, Mitsubishi pharmaceuticals). Additions/amendments in bold and underlined:   5.1 Investigational Medicinal Product (IMP): Tissue Plasminogen Activator (TPA, Alteplase**~~, Actilyse~~)**  Tissue plasminogen activator (TPA) is a 70k Da glycoprotein enzyme that activates plasminogen to plasmin, which in turn breaks down fibrin clots. Alteplase is a commercially produced TPA manufactured using a recombinant DNA technique and a Chinese hamster ovary cell line. **Under the brand names Actilyse^®^ and Actilyse^®^ Cathflo^®^ marketed by Boehringer-Ingelheim**, alteplase is licensed in the UK and EU for the treatment of myocardial infarction, acute ischaemic stroke, and pulmonary embolism **(for 10 mg, 20 mg and 50 mg Actilyse^®^)** **and** for the thrombolytic treatment of occluded central venous access devices **(for 2 mg Actilyse^®^ Cathflo^®^) respectively**. Alteplase is not licensed for the treatment of submacular clots. **It is intended** that TIGER will use vials containing 10 mg of Actilyse**^®^** alteplase in powdered form, packaged with a diluent (10 mls of water for injection), or Actilyse**^®^** Cathflo**^®^** 2 mg in powdered form which contain 2.2 mg total dry weight of Alteplase active agent packaged without a diluent.  **In the event that sites are unable to access Actilyse^®^ and Actilyse^®^ Cathflo^®^, alternative forms of unlicensed alteplase that do not have marketing authorisation in the UK or EU may be used for TIGER, according to what sites have access to, and pending approval from the study management team. The following brands of unlicensed alteplase have the same excipients and are molecularly identical to Actilyse^®^, and will be suitable for use in the TIGER study:**   - **Activase^®^ alteplase 10 mg, 20 mg, 50 mg** - **Activase^®^ Cathflo^®^ 2 mg** - **Activacin^®^ 50 mg, 100 mg** - **GRTPA^®^ 10 mg, 20 mg, 50 mg** | Chan Ning Lee (CRF) | 12.09.2022 |
| 3 | Section 1 “Study Synopsis” | - Removed “Actilyse” from the following sentence to reflect use of non-Actilyse brand alteplase, commensurate to changes above (no. 2). Changes in **bold** and underlined: - Exclusion criterion 2: Hypersensitivity to alteplase **~~(Actilyse)~~**, gentamicin, arginine, phosphoric acid, polysorbate 80 or aflibercept (Eylea). - Intervention: Pars plana vitrectomy, subretinal injection of recombinant TPA (Alteplase, **~~Actilyse, Boehringer Ingelheim~~**~~)~~ up to a maximum of 25 micrograms | Chan Ning Lee (CRF) | 12.09.2022 |
| 4 | Section 2 “Table of Contents” | - Removed “Actilyse” from the following heading to reflect use of non-Actilyse brand alteplase, commensurate to changes above (no. 2). Changes in **bold** and underlined: - Section 5.1 “Investigational Medicine Product (IMP): Tissue Plasminogen Activator (TPA, Alteplase, **~~Actilyse~~**)” | Chan Ning Lee (CRF) | 12.09.2022 |
| 5 | Section 5.2.1 “TPA (Alteplase) dose” | - Removed “(Actilyse)” from the following sentences to reflect use of non-Actilyse brand alteplase, commensurate to changes above (no. 2). Also amended instructions on establishing concentration to be used in study for the purposes of clarity. Changes in **bold** and underlined: - The *maximum* *dose* of alteplase TPA **~~(Actilyse)~~** to be used in TIGER is 25 micrograms, delivered by subretinal injection. - The *concentration* of alteplase TPA **~~(Actilyse)~~** to be used in TIGER is 100 micrograms in 1 ml. **Using 10 mg Actilyse**^®^ **and 2 mg Actilyse**^®^ **Cathflo**^®^ **as examples, the methodology to make up this concentration is as follows:** - **If Actilyse is not available, alternative, molecularly identical brands of alteplase can be substituted, subject to caveats in section 5.1 above.** | Chan Ning Lee (CRF) | 12.09.2022 |
| 6 | Section 5.2.2 “TPA (Alteplase) Injection and Surgical Technique, Required Surgeon Experience, and Timing of Surgery” | - Removed “Actilyse” from the instructions to reflect use of non-Actilyse brand alteplase, commensurate to changes above (no. 2). Changes in **bold** and underlined: - The concentration of alteplase TPA **~~(Actilyse)~~** to be used in TIGER is 100 micrograms in 1 ml. This can be pre-prepared as noted above in Section 5.2.1 | Chan Ning Lee (CRF) | 12.09.2022 |
| 7 | Section 5.6 “IMP risks” | - Added wording to reflect use of non-Actilyse brand alteplase, commensurate to changes above (no. 2). Changes in **bold** and underlined: - **In the event an alternative, non-Actilyse brand of alteplase is used, it is anticipated that if the manufacturing process is identical to Actilyse, and the drug is molecularly identical to Actilyse with the same excipients, the IMP risks will be same as laid out in the SmPC. Reference safety information for non-Actilyse alteplase is available in the respective monographs for each brand of alteplase. Use of other alteplase brands for TIGER will be subject to caveats as laid out in section 5.1.** | Chan Ning Lee (CRF) | 12.09.2022 |
| 8 | Section 5.6 “Drug Accountability and Disposal” | - Added wording to reflect use of non-Actilyse brand alteplase, commensurate to changes above (no. 2). Changes in **bold** and underlined: - **In the event an alternative, non-Actilyse brand of alteplase is approved for use at the site subject to caveats as laid out in section 5.1, these instructions for Drug Accountability and Disposal apply equally.** | Chan Ning Lee (CRF) | 12.09.2022 |
| 9 | Section 5.8 “Storage of IMP” | - Added wording to reflect use of non-Actilyse brand alteplase, commensurate to changes above (no. 2). Changes in **bold** and underlined: - **In the event an alternative, non-Actilyse brand of alteplase is approved for use at the site subject to caveats as laid out in section 5.1, storage instructions should defer to the respective drug monographs as they may differ to the Actilyse SmPC.** | Chan Ning Lee (CRF) | 12.09.2022 |
| 10 | Section 6.2 “Exclusion Criteria” | - Removed “Actilyse” from the following sentence to reflect use of non-Actilyse brand alteplase, commensurate to changes above (no. 2). Changes in **bold** and underlined: - 2. Hypersensitivity to alteplase **~~(Actilyse)~~**, gentamicin, arginine, phosphoric acid, polysorbate 80 or aflibercept (Eylea). | Chan Ning Lee (CRF) | 12.09.2022 |
| 11 | Section 9.1 “Safety Parameters” | - Amended wording to reflect use of non-Actilyse brand alteplase, commensurate to changes above (no. 2). Changes in **bold** and underlined: - Reference safety information **for Actilyse** is available in section 4.8 of the Actilyse Summary of Product Characteristics dated May 2019 as approved. **Reference safety information for non-Actilyse alteplase is available in the respective monographs for each brand of alteplase**. Causality should be determined by the site’s attending clinical investigator, in discussion with the Principal and/or Chief Investigator if necessary. | Chan Ning Lee (CRF) | 12.09.2022 |
| 12 | Section 9.2.3 “Unexpected Adverse Reaction (UAR)” | - Amended wording to reflect use of non-Actilyse brand alteplase, commensurate to changes above (no. 2). Changes in **bold** and underlined: - An adverse reaction, the nature and severity of which is not consistent with the information about Actilyse set out in the relevant Summary of Product Characteristics (SmPC), **or in the respective monograph for the specific brand of alteplase, in the event a non-Actilyse brand of alteplase is used**. | Chan Ning Lee (CRF) | 12.09.2022 |
