# Supplementary material for: Vitrectomy, subretinal Tissue plasminogen activator and Intravitreal Gas for submacular haemorrhage secondary to Exudative Age-Related macular degeneration (TIGER): update to study protocol and addition of a statistical analysis plan and health economic analysis plan for a randomised controlled surgical trial
Source: Trials. 2025 Apr 14;26:131. doi: 10.1186/s13063-025-08727-8 (PMC11995560; doi:10.1186/s13063-025-08727-8)
Supplement: Supplementary file 4 — Additional file 4: Appendix 4. Summary of Changes in TIGER Master Protocol version 3.0. [file 13063_2025_8727_MOESM4_ESM.docx]

**TIGER Master Protocol v3.0 List of Tracked Changes**

| **No** | **Section** | **Reason for Change** (additions/amendments in **BOLD + UNDERLINED**) | **Person enacting change** | **Date of Change** |
| --- | --- | --- | --- | --- |
| 1 | Section 12 “Health Economics Analysis” | Typo – “A” missing from “TPA”. Corrected. | Chan Ning Lee (CRF) | 18.05.2022 |
| 2 | Section 5.1 “Investigational Medicinal Product (IMP): Tissue Plasminogen Activator (TPA, Alteplase, Actilyse)” | Additions/amendments in bold and underlined:   - 5.1 Investigational Medicinal Product (IMP): Tissue Plasminogen Activator (TPA, Alteplase**~~, Actilyse~~)** - Tissue plasminogen activator (TPA) is a 70k Da glycoprotein enzyme that activates plasminogen to plasmin, which in turn breaks down fibrin clots. Alteplase is a commercially produced TPA manufactured using a recombinant DNA technique and a Chinese hamster ovary cell line. **Under the brand names Actilyse and Actilyse Cathflo marketed by Boehringer-Ingelheim**, alteplase is licensed in the UK and EU for the treatment of myocardial infarction, acute ischaemic stroke, and pulmonary embolism **(for 10 mg, 20 mg and 50 mg Actilyse)** **and** for the thrombolytic treatment of occluded central venous access devices **(for 2 mg Actilyse Cathflo) respectively**. Alteplase is not licensed for the treatment of submacular clots. **It is intended** that TIGER will use vials containing 10 mg of Actilyse alteplase in powdered form, packaged with a diluent (10 mls of water for injection), or Actilyse Cathflo 2 mg in powdered form which contain 2.2 mg total dry weight of alteplase active agent packaged without a diluent. | Chan Ning Lee (CRF) | 28.03.2023 |
| 3 | Section 1 “Study Synopsis” | - Removed “Actilyse” from the following sentence. Changes in **bold** and underlined: - Exclusion criterion 2: Hypersensitivity to alteplase **~~(Actilyse)~~**, gentamicin, arginine, phosphoric acid, polysorbate 80 or aflibercept (Eylea). - Intervention: Pars plana vitrectomy, subretinal injection of recombinant TPA (alteplase, **~~Actilyse, Boehringer Ingelheim~~**~~)~~ up to a maximum of 25 micrograms | Chan Ning Lee (CRF) | 12.09.2022 |
| 4 | Section 2 “Table of Contents” | - Removed “Actilyse” from the following heading. Changes in **bold** and underlined: - Section 5.1 “Investigational Medicine Product (IMP): Tissue Plasminogen Activator (TPA, alteplase, **~~Actilyse~~**)” | Chan Ning Lee (CRF) | 12.09.2022 |
| 5 | Section 5.2.1 “TPA (Alteplase) dose” | - Removed “(Actilyse)” from the following sentences. Also amended instructions on establishing concentration to be used in study for the purposes of clarity. Changes in **bold** and underlined: - The *maximum* *dose* of alteplase TPA **~~(Actilyse)~~** to be used in TIGER is 25 micrograms, delivered by subretinal injection. - The *concentration* of alteplase TPA **~~(Actilyse)~~** to be used in TIGER is 100 micrograms in 1 ml. **Using 10 mg Actilyse and 2 mg Actilyse Cathflo as examples, the methodology to make up this concentration is as follows:** | Chan Ning Lee (CRF) | 12.09.2022 |
| 6 | Section 5.2.2 “TPA (Alteplase) Injection and Surgical Technique, Required Surgeon Experience, and Timing of Surgery” | - Removed “Actilyse” from the instructions. Changes in **bold** and underlined: - The concentration of alteplase TPA **~~(Actilyse)~~** to be used in TIGER is 100 micrograms in 1 ml. This can be pre-prepared as noted above in Section 5.2.1 | Chan Ning Lee (CRF) | 12.09.2022 |
| 7 | Section 6.2 “Exclusion Criteria” | - Removed “Actilyse” from the following sentence. Changes in **bold** and underlined: - 2. Hypersensitivity to alteplase **~~(Actilyse)~~**, gentamicin, arginine, phosphoric acid, polysorbate 80 or aflibercept (Eylea). | Chan Ning Lee (CRF) | 12.09.2022 |
| 8 | Section 9.1 “Safety Parameters” | - Amended wording. Changes in **bold** and underlined: - Reference safety information **for Actilyse** is available in section 4.8 of the Actilyse Summary of Product Characteristics dated May 2019 as approved… | Chan Ning Lee (CRF) | 12.09.2022 |
| 9 | Section 1 “Study Synopsis” | - Added wording at bottom of exclusion list to clarify exclusion criterion (6). Changes in **bold** and underlined: - International Normalised Ratio (INR) greater than 3.5, unless it is anticipated that the INR can be brought below this level prior to vitrectomy, balancing the systemic risks with those of intraocular haemorrhage.***** - *** Applies only to participants receiving warfarin. See section 6.2.1 below** | Chan Ning Lee (CRF) | 04.10.2022 |
| 10 | Section 6 “Selection and Withdrawal of Subjects” | - Added wording at bottom of exclusion list to clarify exclusion criterion (6). Changes in **bold** and underlined: - International Normalised Ratio (INR) greater than 3.5, unless it is anticipated that the INR can be brought below this level prior to vitrectomy, balancing the systemic risks with those of intraocular haemorrhage.***** - *** Applies only to participants receiving warfarin. See section 6.2.1 below.** | Chan Ning Lee (CRF) | 04.10.2022 |
| 11 | Section 6.2.1 “INR testing” | - Added subjection to specify which situations INR should be checked. Additions in **bold** and underlined:   **Exclusion criterion 6 notes: *(6) International Normalised Ratio (INR) greater than 3.5, unless it is anticipated that the INR can be brought below this level prior to vitrectomy, balancing the systemic risks with those of intraocular haemorrhage*.**  **This only relates to patients on warfarin, and the international normalised ratio (INR) need not be considered or tested otherwise. For patients receiving warfarin, sites should establish their INR at screening either by testing or review of recent INR results. If the most recent INR was within 4 weeks, and there have been no changes to warfarin dosing within 8 weeks, then no further testing is required and the historic INR can be used as baseline.**  **Additional pre-operative testing of INR can be undertaken as per local standard practice.**  **INR can be tested using the site’s usual method, including either venous blood sampling or finger-prick.** | Chan Ning Lee (CRF)  +  Prof Tim Jackson (CI) | 04.10.2022 |
| 12 | Section 7.2.1 Screening and Baseline Treatment | - Added additional text to clarify that autofluorescence and colour fundus photographs must be repeated at the time of delayed angiography. Additions in **bold** and underlined:   Fluorescein and indocyanine green (ICG) angiography in both eyes (either or both can be delayed as long as necessary to allow the SMH to clear sufficiently to enable visualisation the underlying structures)… **If angiography is delayed, fundus autofluorescence and stereo fundus photography should be repeated alongside the repeat angiogram and sent to the Reading Centre, to facilitate multimodal analysis.** | Chan Ning Lee (CRF) | 09.11.2022 |
| 13 | Section 6.2 “Exclusion Criteria” | - Changed criterion 5 per regulatory authority feedback to bring in-line with aflibercept SmPC. Additions in **bold** and underlined:   Any sexually active women of childbearing potential must agree to continued abstinence from heterosexual intercourse or to use highly effective methods of birth control for the duration up to 12 weeks **after administration of IMP or the last administration of aflibercept on the trial**… | Chan Ning Lee (CRF) | 05.01.2023 |
| 14 | Section 6.5 “Recruitment and Informed Consent” | - Added subsection (Section 6.5) to describe recruitment and consent procedures to bring protocol in-line with European regulatory authority requirements. Additions in **bold** and underlined:   **Each site’s principle investigator (PI) or delegated sub-investigators (SI) will be responsible for approaching and recruiting potential trial participants and obtaining informed consent prior to undertaking any trial-related activities. All informed consent procedures will be undertaken by a qualified, registered, good clinical practice (GCP) certified clinician who is on the site delegation log.**  **Potential participants will mostly be identified from referrals from clinicians to trial vitreoretinal centres, or otherwise from emergent diseases within the PI’s own clinic population. As potential participants will have been referred to, or already be under the care of the study team as part of standard care, any identifiable information used to identify them will be protected as part of local information governance policies. All potential participants will be contacted in advance of their clinic appointment and sent a study patient information sheet (PIS), or on the day of appointment and given a copy of the PIS. All potential participants will have the purpose, risks and benefits of the trial explained by the PI or SI in person, who will also confirm capacity, before signing a trial-specific informed consent form (ICF) and undertaking screening activities.**  **Where possible, all efforts will be given to allow potential participants at least 24 hours to decide whether to take part in the trial, however as blood is toxic to photoreceptors, and delays may mean surgery is less effective leading to risks of surgery outweighing the potential benefits, it is important to expedite screening and surgery as much as possible.**  **In the event a potential participant does not speak the national language, local site-specific language translation services will be utilised as part of the informed consent process and throughout the trial.** | Chan Ning Lee (CRF) | 09.01.2023 |
| 15 | Section 13.1 “Patient Involvement Group” | - Changed “Informed Consent Form” to “**ICF**” in-line with earlier acronymization. | Chan Ning Lee (CRF) | 09.01.2023 |
| 16 | Section 14 “Trial Steering Committee” | - Changed “Principal Investigator” to “**PI**” in-line with earlier acronymization. | Chan Ning Lee (CRF) | 09.01.2023 |
| 17 | Section 16 “Access to Source Data and Documents” | - Changed “Principal Investigator” to “**PI**” in-line with earlier acronymization. | Chan Ning Lee (CRF) | 09.01.2023 |
| 18 | Section 29 “Appendix D: List of abbreviations” | - Added “Sub-Investigator” | Chan Ning Lee (CRF) | 09.01.2023 |
| 19 | Section 9.3.3 “Trial Discontinuation" | - Added specific trial discontinuation wording to bring protocol in-line with European regulatory authority requirements. Additions in **bold** and underlined:   The trial may be prematurely discontinued by the Sponsor, Chief Investigator or Regulatory Authority on the basis of new safety information **emerging from published clinical trials or toxicological studies which negatively influence the risk/benefit assessment of the IMP or procedure, or safety data emerging from the trial itself such as the occurrence of ocular or systemic adverse events whose character, severity or frequency is unexpected in relation to the known safety profile of the IMP or procedure, for example, an unexpectedly high incidence of post-operative ocular haemorrhage. The trial may also be prematurely discontinued** on the guidance/direction of the relevant Data Monitoring Committee, Research Ethics Committee, Trial Steering Committee, or Competent Authority. | Chan Ning Lee (CRF) | 10.01.2023 |
| 20 | Section 5.6 “IMP Risks” | - Changed expectedness of ocular and systemic adverse events related to Actilyse in keeping with systemic and local exposure. Additions in **bold** and underlined. - …Hence systemic drug levels are thought very unlikely to pose a risk outside of the eye**, and the** undesirable effects described in the SmPC **will be considered** ‘**un**expected’ **if they occur.** “**E**xpected” risks based on the anticipated, accepted or reported risks particular to the action and/or delivery of Actilyse in the eye include:… | Chan Ning Lee (CRF) | 10.01.2023 |
| 21 | Section 1 “Study Synopsis” | - Changed criterion 5 per regulatory authority feedback to bring in-line with aflibercept SmPC. Additions in **bold** and underlined:   Any sexually active women of childbearing potential must agree to continued abstinence from heterosexual intercourse or to use highly effective methods of birth control for the duration up to 12 weeks **after administration of IMP or the last administration of aflibercept on the trial**… | Chan Ning Lee (CRF) | 13.01.2023 |
| 22 | Section 7.1 “Schedule of Activitis by Visit Table” | - Added instructions to clarify whether masked or unmasked assessors should undertake Clinic or Full refracted ETDRS VA at which time points. Additions in **bold** and underlined:   $ Full refracted ETDRS VA is undertaken in both eyes separately at baseline and month 12 and study eye only at month 6. **All full refracted ETDRS VA assessors should be masked to trial arm**. Details in Appendix A.  † Clinic ETDRS VA should be undertaken in the study eye only using an ETDRS chart with correction of any refractive error, with and without pinhole. **This should be undertaken by unmasked assessors at D1 and W1 in arm A participants, and M1 in both arms. Thereafter from M2, only masked assessors should undertake Clinic ETDRS VA.** Detail in Appendix A. | Chan Ning Lee (CRF) | 20.01.2023 |
| 23 | Secion 7.2.2 “Day 1 and 7 (Arm A and study eye only)” | - Added wording to clarify this should be undertaken by unmasked assessors. Additions in **bold** and underlined:   Day 1 and week 1 (day 7) postoperative review, for those in the surgical arm, comprises Clinic ETDRS VA in the study eye **by unmasked assessors** (details in Appendix A)… | Chan Ning Lee (CRF) | 20.01.2023 |
| 24 | Section 7.2.3 “Month 1 and 2” | - Added wording to clarify that M1 Clinic ETDRS VA should be undertaken by unmasked assessors whilst from M2 onwards, all assessment should be undertaken by masked assessors. Additions in **bold** and underlined:   **Month 1 Clinic ETDRS VA should be undertaken by an unmasked assessor, but from Month 2 onwards, all Clinic ETDRS VA assessments should be undertaken by masked assessors.** | Chan Ning Lee (CRF) | 20.01.2023 |
| 25 | Section 7.2.4 “Month 4”, Section 7.2.6 “Month 8” and section 7.2.7 “Month 10” | - Added wording to clarify that M4, 8, 10 Clinic ETDRS VA should be undertaken by masked assessors. Additions in **bold** and underlined:   Clinic ETDRS VA in study eye **by masked assessors** (Appendix A)… | Chan Ning Lee (CRF) | 20.01.2023 |
| 26 | Section 7.2.5 “Month 6”, Section 7.2.8 “Month 12” | - Added wording to clarify that M6 and 12 Full refracted ETDRS BCVA should be undertaken by masked assessors. Additions in **bold** and underlined:   Full refracted ETDRS BCVA **by masked assessors**.. | Chan Ning Lee (CRF) | 20.01.2023 |
| 27 | Section 5.1 “Investigational Medicinal Product (IMP): Tissue Plasminogen Activator (TPA, Alteplase)” | - Replaced wording referencing Trial Pharmacies as some sites may not have dedicated Trials Pharmacies. Additions in **bold** and underlined:   TIGER aims to select sites that already have access to alteplase for use within its marketing authorisation. Stock alteplase will be relabelled by ~~the~~ site**s**~~’s Trials Pharmacy~~ according to Annex 13 of Good Manufacturing Practice… | Chan Ning Lee (CRF) | 28.02.2023 |
| 28 | Section 5.7 “Drug Accountability and Disposal” | - Replaced wording referencing Trial Pharmacies as some sites may not have dedicated Trials Pharmacies. Additions in **bold** and underlined:   The drug will be labelled by ~~the~~ **sites** ~~Trials Pharmacy~~ and then supplied directly to the operating team, most likely on the day of surgery…  Alternatively, storage of Actilyse can be delegated ~~by the local Trials Pharmacy~~ according to the Site’s usual practice… | Chan Ning Lee (CRF) | 28.02.2023 |
| 29 | Section 5.4.2 “Aflibercept (Eylea)” | - Additional instructions to clarify what sites should do if participants require additional Eylea to study-mandated treatment intervals. Additions in **bold** and underlined:   **It is possible, though unlikely, that participants require additional Eylea injections in their study eye due to poorly controlled exudative AMD in between protocol-mandated dosing intervals up to Month 12. If investigators decide it is essential to administer additional Eylea injections, these should be recorded as a deviation and each injection entered as a separate entry in the concomitant medications log on the source data worksheets and eCRF.** | Chan Ning Lee (CRF) | 28.03.2023 |
