# Supplementary material for: Vitrectomy, subretinal Tissue plasminogen activator and Intravitreal Gas for submacular haemorrhage secondary to Exudative Age-Related macular degeneration (TIGER): update to study protocol and addition of a statistical analysis plan and health economic analysis plan for a randomised controlled surgical trial
Source: Trials. 2025 Apr 14;26:131. doi: 10.1186/s13063-025-08727-8 (PMC11995560; doi:10.1186/s13063-025-08727-8)
Supplement: Supplementary file 5 — Additional file 5: Appendix 5. TIGER statistical analysis plan. [file 13063_2025_8727_MOESM5_ESM.pdf]

# TIGER Trial

Vitrectomy, subretinal Tissue plasminogen activator and Intravitreal Gas for submacular haemorrhage secondary to Exudative age-Related macular degeneration (TIGER): a phase 3, pan-European, two-group, active-control, observer-masked, superiority, randomised controlled surgical trial.

Statistical Analysis Plan
Version 1.0 (finalised 30/03/2022)
*Using protocol version 2.0*

ClinicalTrial.gov identifier: NCT04663750

---


**Prepared by:**

Hatem Wafa
Statistician
School of Life Course & Population Health Sciences
King's College London
Addison House, Guy's Campus
London, SE1 1UL
Tel: 020 7848 6623
Email: hatem.a.wafa@kcl.ac.uk

**Approved by:**

Yanzhong Wang
Reader in Medical Statistics
School of Life Course & Population Health Sciences
King's College London
Addison House, Guy's Campus
London, SE1 1UL
Tel: 020 7848 8223
Email: yanzhong.wang@kcl.ac.uk


Signature:

Date:

Signature:

Date: 30th March 2023

## Investigators

**Chief Investigator**
Prof Tim Jackson
King's College London
Department of Ophthalmology
King's College Hospital NHS Foundation Trust
Denmark Hill
London
Tel: +44 (0) 20 3299 1297
Email: t.jackson1@nhs.net

**Clinical Co-Investigators**
Prof David Steel
The University of Newcastle upon Tyne
Tel: +44 (0) 191 5699065
Email: David.steel@newcastle.ac.uk

Prof Noemi Lois
Queen's University Belfast
Tel: +44 (0)28 9097 6462
Email: n.lois@qub.ac.uk

**Co-Sponsors**
King's College London &
King's College Hospital NHS Foundation Trust
Amy Holton
King's Health Partners Clinical Trials Office
Floor 16, Tower Wing,
Guy's Hospital,
London
Tel: 07703469925
Email: Amy.holton@kcl.ac.uk

**Trial Methodologist**
Prof Barnaby Reeves
University of Bristol
Tel: +44 (0) 117 34 23143
Email: Barney.Reeves@bristol.ac.uk

**Lead Statistician**
Dr Yanzhong Wang
King's College London
Tel: +44 (0) 20 7848 8223
Email: yanzhong.wang@kcl.ac.uk

**Reading Centre Lead**
Prof Tunde Peto
Network of Ophthalmic Reading Centres UK
Central Administrative Research Facility
Queen's University Belfast,
Northern Ireland.
Tel: +44 (0) 28 9097 1659
Email: T.Peto@qub.ac.uk

**Health Economist**
Prof Rhiannon Tudor Edwards
Co-Director Centre for Health Economics and Medicines Evaluation
Bangor University,
Bangor, Gwynedd,
Tel: +44 (0) 1248 383712
Email: r.t.edwards@bangor.ac.uk

## CONTENTS

This document contains up to date statistical analysis plans (with version numbers and dates).

A) Quantitative Analysis Plan
B) Schedule of Assessments and Measures

Note: Health Economics Analysis Plan (HEAP) is detailed in a separate document.


**A) QUANTITATIVE ANALYSIS PLAN ... 4**
**1 Description of the trial ... 4**
1.1 Principal research objectives to be addressed ... 4
1.1.1 Primary objectives ... 4
1.1.2 Secondary objectives ... 4
1.2 Trial design and flowchart ... 4
1.3 Method of allocation into groups ... 6
1.4 Study duration and frequency of follow up ... 6
1.5 Data collection ... 6
1.5.1 Eligibility screening ... 6
1.5.2 Efficacy Measures ... 8
1.5.3 Safety Outcome Measures ... 9
1.6 Sample size estimation (including clinical significance) ... 9
1.7 Brief description of proposed analyses ... 9
**2 Data analysis plan – Data description ... 10**
2.1 Recruitment and representativeness of recruited patients ... 10
2.2 Baseline comparability of randomised groups ... 10
2.3 Loss to follow-up on outcome data ... 10
2.4 Adverse event reporting ... 10
2.5 Descriptive statistics for outcome measures ... 10
**3 Data analysis plan – Inferential analysis ... 11**
3.1 Main analysis of treatment differences ... 11
3.1.1 Analysis of primary outcomes ... 11
3.1.2 Analysis of secondary outcomes ... 11
3.1.3 Planned subgroup analyses ... 12
3.1.4 Image Analysis ... 12
3.2 Statistical considerations ... 13
3.2.1 Missing outcome data ... 13
3.2.2 Method for handling non-compliance ... 13
3.2.3 Method for handling non-conformity in randomisation ... 14
3.3 Exploratory analyses ... 14
3.4 Interim analysis ... 14
3.5 Software ... 14
**Reference List ... 18**
**Appendix A: Database variables ... 19**
**Appendix B: Compliance rules ... 21**

# A) QUANTITATIVE ANALYSIS PLAN

## 1 Description of the trial

The key objective of the TIGER study is to assess the safety and efficacy of vitrectomy, subretinal tissue plasminogen activator (TPA), and gas tamponade as an adjunct treatment to the standard of care for submacular haemorrhage (SMH) secondary to exudative age-related macular degeneration (AMD), versus the standard of care with anti-vascular endothelial growth factor (anti-VEGF).

This study aims to enrol 210 participants in a phase 3, multicentre, pan-European, non-commercial, randomised, two-group, active control, superiority, observer-masked, surgical trial.

### 1.1 Principal research objectives to be addressed

Hypothesis: For patients with SMH secondary to wet AMD, subretinal TPA, together with intravitreal gas tamponade and anti-VEGF therapy, results in superior visual acuity outcome, compared with anti-VEGF monotherapy.

Aim: To determine the safety and efficacy of vitrectomy, subretinal TPA, and gas tamponade.

#### *1.1.1 Primary objectives*

To estimate:

- If there is a statistically significant difference between the treatment groups in terms of the proportion of participants gaining ≥10 Early Treatment of Diabetic Retinopathy Study (ETDRS) letters of best-corrected visual acuity (BCVA) in the study eye at the month 12 visit.

#### *1.1.2 Secondary objectives*

To estimate:

- If there is a statistically significant difference between the treatment groups in the proportion of participants gaining ≥10 ETDRS letters of BCVA in the study eye at the month 6 visit.
- If subretinal TPA and gas tamponade are safe.
- If subretinal TPA and gas tamponade are cost-effective.

### 1.2 Trial design and flowchart

Phase 3, multicentre, pan-European, non-commercial, randomised, two-group, active control, superiority, observer-masked, surgical trial.

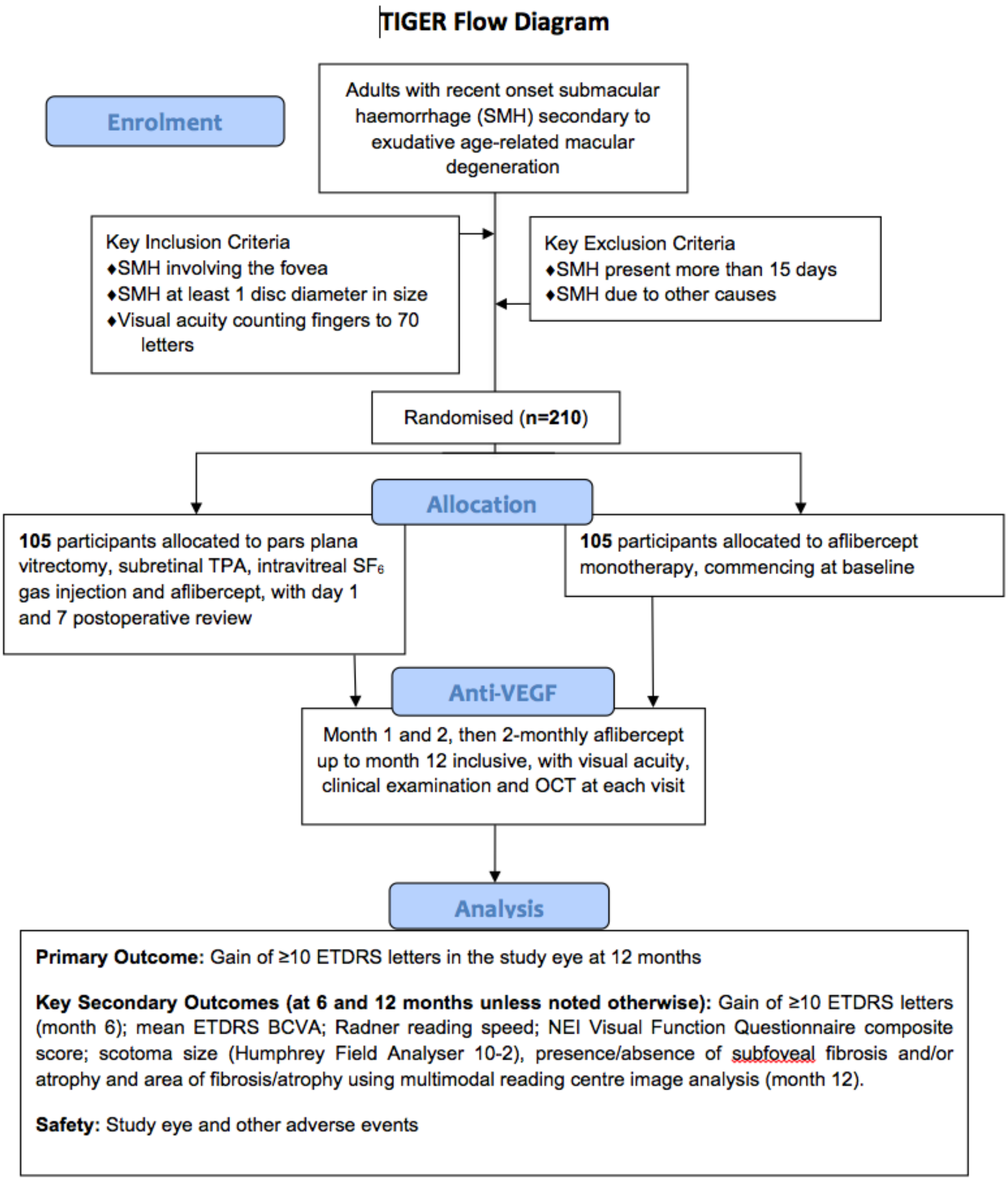


**Figure 1:** Trial design flow diagram

## 1.3 Method of allocation into groups

Once baseline assessments are complete, participants will be randomized in a 1:1 ratio using the method of minimisation. Randomisation is at the patient level and is performed using a web-based bespoke randomisation system set up by the King's Clinical Trials Unit (KCTU) at King's College London. Randomisation is minimised by the following factors:

- Study site
- Lens status: phakic or pseudophakic
- SMH size: fully within the retinal vascular arcades, or not
- BCVA: ≥35 letters or not, equivalent to 6/60 and near to the median presenting BCVA in the TAPAS study (ClinicalTrials.gov identifier: NCT01835067)

It is not possible to mask participants to their allocation because there is no sham for vitrectomy, and intravitreal gas is easily visible to participants. However, the BCVA primary outcome (BCVA at 12 months) will be assessed by masked observers using an established protocol, encouraging participants to "try their hardest", to minimise any differences in decision criterion and measurement bias. Secondary BCVA assessments will also be undertaken by masked observers.

## 1.4 Study duration and frequency of follow up

Participants will be enrolled for a total period of 12 months (from baseline treatment to last visit). During that time, participants will be followed up at 1 day (surgical group only), 1 week (surgical group only), 1 month, 2 months and then every 2 months.

## 1.5 Data collection

The trial will randomize 210 patients with SMH secondary to wet AMD. After giving fully informed written consent, patients will be screened for participation in the study. Patients should fulfil the following criteria to be eligible for enrolment:

### 1.5.1 *Eligibility screening*

***General inclusion criteria***

1. Males or females aged at least 50 years

***Study eye inclusion criteria***

2. SMH, comprising sub-neuroretinal haemorrhage with or without sub-RPE haemorrhage, that occurs secondary to treatment naïve, or previously treated exudative AMD, including choroidal neovascularisation (CNV), idiopathic polypoidal choroidal vasculopathy (IPCV) and retinal angiomatous proliferation (RAP).

3. SMH involving the foveal centre that measures at least 1 disc diameter in greatest linear dimension.

4. Sub-neuroretinal haemorrhage at least 125 microns thick, measured at the foveal centre using spectral-domain optical coherence tomography (SD-OCT).
5. BCVA between counting fingers and an Early Treatment of Diabetic Retinopathy Study (ETDRS) letter score of 70, inclusive.

***General exclusion criteria***

1. Serious allergy to fluorescein or indocyanine green (ICG).
2. Hypersensitivity to alteplase, gentamicin, arginine, phosphoric acid, polysorbate 80 or aflibercept (Eylea).
3. Stroke, transient ischaemic attack or myocardial infarction within 6 months.
4. Participation in another interventional study within 12 weeks of enrolment or planned to occur during this study.
5. Women who are breast feeding, pregnant, or planning to become pregnant during the clinical trial. Any sexually active women of childbearing potential must agree continued abstinence from heterosexual intercourse or to use highly effective methods of birth control for the duration up to 12 weeks after administration of IMP or the last administration of aflibercept on the trial. Men must also agree to use a condom if their partner is of child-bearing potential, even if they have had a successful vasectomy. Females of childbearing potential are females who have experienced menarche and are not surgically sterilised (e.g. hysterectomy or bilateral salpingectomy) or post-menopausal (defined as at least 1 year since last regular menstrual period). Highly effective methods of birth control are those with a failure rate of < 1% per year when employed consistently and correctly, eg. combined (oestrogen and progestogen containing) hormonal contraception associated with inhibition of ovulation via oral, intravaginal, and transdermal routes; progestogen-only hormonal contraception associated with inhibition of ovulation via oral, injectable, implantable, intrauterine device (IUD), or intrauterine hormone-releasing system (IUS); or vasectomised partner.
6. International Normalised Ratio (INR) greater than 3.5, unless it is anticipated that the INR can be brought below this level prior to vitrectomy, balancing the systemic risks with those of intraocular haemorrhage.*
7. Unwilling, unable, or unlikely to return for scheduled follow-up for the duration of the trial.
8. Any other condition which, in the opinion of the investigator, would prevent the participant from granting informed consent or complying with the protocol, such as dementia, mental illness, or serious systemic medical disease.

***Study eye exclusion criteria***

9. SMH that is known or estimated to have been present for longer than 15 days, as evidenced by history, pre-trial clinical documentation, or fundus appearance.
10. SMH due to eye disease other than exudative AMD.
11. Current active proliferative diabetic retinopathy.
12. Current intraocular inflammation.
13. Current ocular or periocular infection other than blepharitis.
14. Current or known former high myopia (>6 dioptres).
15. Aphakia.
16. Other current or pre-existing ocular conditions that, in the opinion of the Investigator, will preclude any improvement in BCVA following resolution of SMH, such as severe central macular atrophy or fibrosis, dense amblyopia, macular hole involving the fovea, or very poor BCVA prior to presentation with SMH (counting fingers or worse).
17. Inadequate pupillary dilation or significant media opacities, which will prevent adequate clinical evaluation of the posterior segment or fundus imaging.
18. Intraocular surgery within 12 weeks of enrolment except for uncomplicated cataract surgery, which is permitted within 8 weeks of enrolment.

*Applies only to participants receiving warfarin.

### *1.5.2 Efficacy Measures*

The following outcomes will be reported.

***Primary Measures***

- Gain ≥10 ETDRS letters of BCVA in the study eye at the month 12 visit.

***Secondary Measures***

The following secondary outcomes will be reported, with respect to the study eye, in addition to the National Eye Institute 25-item visual function questionnaire (NEI VFQ-25).

- Vision gain ≥10 ETDRS letters (at the 6 month visit).
- Mean ETDRS BCVA (6 and 12 month).
- Radner maximum reading speed (6 and 12 months).
- Area of scotoma size using Humphrey Field Analyser 10-2 or equivalent (6 and 12 months).
- NEI VFQ-25 composite score (6 and 12 months).

- Presence or absence of subfoveal fibrosis and/or atrophy and area of fovea-involving fibrosis/atrophy assessed using multimodal imaging by an independent reading centre, combining spectral domain optical coherence tomography (SD-OCT), fundus autofluorescence (FAF) and stereo fundus photographs (month 12).

### *1.5.3 Safety Outcome Measures*

Safety will be evaluated by assessing adverse events (AEs) and serious adverse events (SAEs). The percentage of participants and study eyes in which AEs are observed will be reported within each group with 95 % confidence intervals. The trial will specifically report the incidence of cataract, retinal tear/detachment, elevated IOP (>25 mmHg), and hypotony (IOP <5 mmHg). The number of intraocular eye operations will be recorded for both groups.

## 1.6 Sample size estimation (including clinical significance)

To calculate our sample size we undertook a bracketing exercise with our patient focus group. We asked them what improvement in 'treatment success', defined as meaningful gain in vision, they would require to undergo eye surgery, with the attendant downsides such as discomfort, head positioning, complications, recovery, and possible cataract surgery, and assuming a 1 in 4 chance of success with anti-VEGF injections alone.[1]

The findings ranged from one patient who would not want surgery 'at my age', regardless of outcome, to another who would have surgery even if it improved his success from only 25% to 26%. The most common response was that patients wanted at least a 50% chance of success to consider vitrectomy.

What also emerged in this elderly group was a consensus that they wanted the doctor to make the best decision on their behalf. Paradoxically then, our public patient involvement (PPI) exercise led us to ask 10 Ophthalmologists, from junior to senior, across a range of subspecialities, what they would do, 'if it was their eye'. We asked them to assume they were older adults with ≈25% success with anti-VEGF therapy. The median 'success rate' (defined as a 10 letter gain) needed to consider surgery differed if it was their potentially better or worse seeing eye (47.5% vs 55% respectively), but overall the average/median/mode were 49%, 50% and 50% respectively. Thus we used 50% as the minimum success rate needed to justify surgery.

Our synthesis of the literature[1] found that 27% of patients receiving anti-VEGF monotherapy for AMD-related SMH gained 2 Snellen lines (≈10 letters).

A two group $\chi^2$ test with a 5% two-sided significance level has 90.62% power to detect a difference between a Group 1 proportion, $\pi 1$, of 0.27 and a Group 2 proportion, $\pi 2$ of 0.5 (odds ratio 2.704) when the sample size in each group is 94 (NQuery Advanced software v 8.2.1). With ≈12% attrition the sample size inflates to 210 participants.

## 1.7 Brief description of proposed analyses

Analyses will be carried out by the trial statistician. In the first instance data will be analysed under intention-to-treat assumptions (i.e. analyse all those with data in groups as randomised irrespective of treatment received). Appendix A shows the range of variables to be extracted from MACRO database and the randomisation system which will be used to derive the measures needed for the analyses described below.

# 2 Data analysis plan – Data description

## 2.1 Recruitment and representativeness of recruited patients

Recruitment, randomisation, and follow-up for TIGER will be summarised by arm in a CONSORT flow-diagram. This will include the main reasons for there being missing data (withdrawal, lost to follow up) by stages of the trial, and will also include the numbers for whom this occurs per arm. Also included will be the number randomised, who comprise the intention to treat trial population, the number received the treatment, to comprise the population for safety evaluation, and the numbers followed-up to be in the analyses of the primary outcome.

## 2.2 Baseline comparability of randomised groups

Baseline characteristics of each group will be summarised as mean and standard deviation for continuous variables with median and interquartile range for highly skewed data, and count and percentage for categorical variables. No significance testing on baseline variables will be performed.

The baseline characteristics will include patient demographics, randomisation stratifiers, ophthalmic history, medical history, drug history, EDTRS BCVA, and other baseline (screening) clinical measures. This will allow an assessment of whether there is clinically important imbalance in any variables.

## 2.3 Loss to follow-up on outcome data

The proportions of participants with any missing data will be summarised by variable in each arm and at each time point. The baseline characteristics of those with missing primary outcome data will be compared statistically to those with complete follow up using appropriate univariate statistical tests. The reasons for withdrawal from the trial will be summarised in the CONSORT flow diagram.

Our study size and power calculations allow for a 12% loss to follow up at the one-year primary endpoint. To address any missingness that occurs, we will conduct a sensitivity analysis of the primary outcome that adjusts for any factors shown to be different between those present and those with full primary outcome data.

## 2.4 Adverse event reporting

Adverse events (AE), adverse reactions (AR), serious adverse events (SAE) and serious adverse reactions (SAR) will be summarised as counts and percentages with 95% confidence intervals by trial arm. Where patients have not received the allocated treatment, this will be noted in reporting AEs so that the denominator for AEs is the number who actually received each treatment.

## 2.5 Descriptive statistics for outcome measures

The following outcomes will be reported:

***Primary Measure***

- Percentage of participants who gain at least 10 ETDRS letters in their study eye at the month 12 visit.

***Secondary Measure***

- Percentage of participants gaining ≥10 ETDRS letters at the 6 months visit.
- Mean ETDRS BCVA (6 and 12 month).
- Radner maximum reading speed (6 and 12 months).
- Area of scotoma size by Humphrey Field Analyser 10-2 or equivalent (6 and 12 months).
- NEI VFQ-25 composite score (6 and 12 months).
- Presence or absence of subfoveal fibrosis and/or atrophy and area of fovea-involving fibrosis/atrophy assessed using multimodal imaging by an independent reading centre, combining spectral domain optical coherence tomography (SD-OCT), fundus autofluorescence (FAF) and stereo fundus photographs (month 12).

Continuous outcome measures (per treatment arm) will be summarised as mean and standard deviation, with median and interquartile range where there is extreme skewness; categorical outcome measures as count and percentage.

# 3 Data analysis plan – Inferential analysis

## 3.1 Main analysis of treatment differences

### *3.1.1 Analysis of primary outcomes*

The principal analyses of primary outcome will be performed according to "intent-to-treat" principle. All randomized patients in these analyses will be classified according to their assigned treatment at randomization, regardless of patient's adherence. The primary analysis is to test the difference between treatment groups in terms of the proportion of participants gaining ≥10 ETDRS letters in their study eye at the month 12 visit. In this case, a multiple logistic regression analysis will be used to assess the treatment effect (odds ratio) with adjustment for the baseline minimisation factors. The treatment effect is evaluated at the two-sided 0.05 significance level.

### *3.1.2 Analysis of secondary outcomes*

Data from the other efficacy outcomes (listed in Section 1.5.2) will be summarized. Statistical analysis of these outcomes will mostly be descriptive, with differences and 95% confidence intervals where possible. Secondary continuous outcomes measured at randomisation and on more than one occasion during follow-up will be analysed using a linear mixed effect model. The value at baseline, treatment group, follow-up time, and the stratifying variables will be included as fixed effects. Model assumptions will be assessed, and a logarithmic transformation used if this improves normality of residuals. Secondary dichotomous outcomes will be examined using the same techniques as the primary analysis.

### *3.1.3 Planned subgroup analyses*

A prespecified subgroup analyses will assess the possible interactions between treatment and each of the following parameters:

- *Lens status (phakic vs pseudophakic):* Removal of pre-existing cataract may improve vision, whereas post-vitrectomy lens opacity that does not trigger cataract surgery in the 12 months' follow-up period may reduce vision. This may impact on our analysis of the effect of surgery. It is much less problematic if we aim for a pragmatic trial design, wherein routine cataract management forms part of the "real world" patient pathway. However, we realise some clinicians and reviewers will want to isolate the effects of vitrectomy from lens events, and for them an analysis of pseudophakic eyes will provide a useful mechanistic insight.
- *SMH size (fully within the retinal vascular arcades, or not):* Many clinicians believe that large SMHs do better with surgery, as vitrectomy, TPA and gas is expected to provide more complete and rapid removal of blood from the fovea. We have chosen this size as it is a pragmatic and clinically useful size differentiator. Outcome has previously been shown to be closely related to the size of the haemorrhage. A SMH of less than 30 $mm^2$ (approximately just up to the arcades) has been shown to be predictive of an outcome of 6/60 or better in cases treated with vitrectomy and subretinal TPA.[2]
- *Lesion type (choroidal neovascularisation vs idiopathic polypoidal choroidal vasculopathy):* Some clinicians consider IPCV as part of the AMD spectrum, others consider it to be a distinct entity. Therefore, we cannot assume the effects of surgery will be the same in those with/without IPCV. IPCV is an important cause of SMH and is over-represented in case series of AMD with SMH. It is more common in Black and Asian patients. Retrospective studies report that SMH due to CNV has a worse outcome than SMH due to IPCV.[3] IPCV is often associated with blood below the RPE, and this space is not accessed via a sub-neuroretinal injection. Therefore, it is possible that surgery may be less effective for IPCV than CNV.
- *Duration of SMH (≤7 days vs >7 days):* It is possible that the benefits of surgery may diminish with a longer duration SMH, if the sustained toxic effects of blood reduce the impact of blood removal, yet the surgical risks remain constant. We chose 7 days as numerous studies have suggested that SMH duration of less than 7 days is associated with an improved visual outcome in surgically treated cases. These studies have typically been retrospective and the time point chosen arbitrarily, based on previous publications and animal studies. Regardless, it is an easy timepoint for clinicians to use and remember if it is found to be important.

These factors will be explored by adding interaction terms to the regression model for the primary outcome.

### *3.1.4 Image Analysis*

The Network of Ophthalmic Reading Centres UK (NetwORC UK), a network of three Ophthalmic Image Reading Centres in the United Kingdom (Belfast, Moorfields Eye Hospital in London, and Liverpool), will be responsible for masked image analysis (https://www.networcuk.com/). NetwORC UK will provide all training materials for

image acquisition and will support sites throughout the trial. Image submission will be via a safe online submission system. All graders involved in TIGER are trained and certified for grading AMD at clinical trials level and will have passed their study specific certification for TIGER before grading commences.

For TIGER, there will be multimodal grading performed using all imaging modalities to enable the grader to provide a grade for relevant AMD-related abnormalities such as haemorrhage, atrophy and fibrosis in the study eye, and an overall AMD-phenotype decision in the study and fellow eye. All data will be entered into the TIGER database by certified NetwORC UK personnel. Quality assurance and quality control will be conducted according to NetwORC UK protocols and will be reported on to the study team and in the final report.

## 3.2 Statistical considerations

### *3.2.1 Missing outcome data*

We will report missingness wherever present. Reasons for missingness may be important and these will be investigated using logistic regression of covariates on an indicator of missingness.

Sensitivity analyses will investigate the validity of the missing data completely at random assumption and will explore imputation for missing data. In relation to the primary outcome variable we will conduct an available case analysis, but will then conduct a worst-case best-case analysis to examine the impact of missing data. The sensitivity analysis will consider participants in the surgery group with missing outcomes having a meaningful change in BCVA and participants in the anti-VEGF monotherapy control group with missing data not having a meaningful change in BCVA, and then the opposite. Our missing data analysis is complete if the results show that they are consistent with the available case analysis. If not, a range of more plausible assumptions will be explored following principles laid out in Carpenter & Kenwood.[4]

We will examine patterns of missingness and the reasons which caused the data to be missing. This will be achieved by examining the observed data and reasons for withdrawal in discussion with the clinical investigators. We will use this information to derive a series of missing data models. We will use these models to impute values in order to undertake a sensitivity analysis of the treatment effect estimate. If data are thought to be missing at random (MAR), conditional on additional variables not included in the primary analysis model, then the treatment effect will be estimated conditioning on the identified variable for example: conditioned on BCVA or size of haemorrhage at baseline. The scenario of missing not at random scenarios (MNAR) will be explored using a range of plausible assumptions and viewed graphically using a mean score approach via the multivariate imputation by chained equations (mice) package in R. The impact of missing data will be mitigated against by incorporating information from earlier timepoints using the mixed model approach.

### *3.2.2 Method for handling non-compliance*

The number of patients who have not completed their full treatment protocol is expected to be few but will be noted. In addition to the primary intention-to-treat analysis, the

effect of actually receiving treatment as defined in the protocol will also be estimated by comparing the two arms in just those who have received the full protocol.

Compliance will be presented graphically for all patients using a novel "compliance cube". This will show the follow-up timeline up to the month 12 primary endpoint as a horizontal line for each participant. Sequential visits will be colour coded according to predefined rules (see appendix B) into green (compliant), amber (some deviation but not one likely to materially affect the primary outcome), or red (deviation that may have affected the primary outcome). Withdrawals will be shown in white and deaths in black. Finally, the colour coded visit timeline for each participant will be stacked one above the other, from first to last participant recruited.

#### *3.2.3 __Method for handling non-conformity in randomisation__*

In the case that randomised treatment code is incorrectly applied by unforeseen reason, we will identify the patients potentially affected and establish which, if any, of those patients received the opposite treatment allocation to that randomised. Analyses will be based on intention-to-treat (ITT). A sensitivity analysis will be carried out using the 'actually received' treatment.

### 3.3 Exploratory analyses

Any examination of subgroups, not specifically identified in the protocol, will be considered exploratory in nature and will be clearly identified.

### 3.4 Interim analysis

The usual rationale for an interim analysis is to consider stopping the treatment (or the trial) however as this treatment is given at baseline, it is not possible to subsequently stop treatment for a given participant. Although, retreatment is allowed, only minority of the patients might require repeated surgery. Due to the estimated recruitment timelines it is likely most participants will have been treated by the time a meaningfully interim analysis of the 12-month primary outcome could be completed. As such we elected not to include an interim analysis. The DMC will examine the recruitment rate, data completeness and monitor safety, and will recommend whether the study should continue, stop, be suspended, or be modified, based on their findings. If necessary for urgent safety reasons the Sponsor may stop or pause the trial immediately, without DMC review.

### 3.5 Software

Data management: An online data collection system for clinical trials (MACRO; InferMed Ltd) will be used. This is hosted on a dedicated server at King's College London and managed by King's CTU (KCTU). The KCTU Data Manager will extract data periodically as needed and provide these in comma separated (.csv) format.

Statistical analysis: Statistical software package R will be used for data description and the main inferential analysis.

## C) SCHEDULE OF ASSESSMENTS AND MEASURES

| Activity | Screening** | Baseline** | D1 | W1 | M1 | M2 | M4 | M6 | M8 | M10 | M12 |
|---|---|---|---|---|---|---|---|---|---|---|---|
| Visit window (±days) | Day -7 to 0 | - | 0 | ±3 | ±7 | ±7 | ±7 | ±7 | ±7 | ±7 | ±7 |
| Consent | **X** | | | | | | | | | | |
| Medical and ophthalmic history | **X** | | | | | | | | | | |
| Randomisation | **X** | | | | | | | | | | |
| Vitrectomy, TPA and gas (Arm A only) | | **Arm A**[§] | | | | | | | | | |
| Intravitreal aflibercept | | X[§] | | | X | X | X | X | X | X | X |
| Full refracted ETDRS BCVA[$] | **X** | | | | | | | **X** | | | **X** |
| Clinic ETDRS VA[†] | | | **Arm A** | **Arm A** | X | X | X | | X | X | |
| Radner reading vision[‡] | **X** | | | | | | | **X** | | | **X** |
| Visual field (HFA 10-2)(sent to reading centre)[¢] | **X** | | | | | | | **X** | | | **X** |
| Slit-lamp examination and intraocular pressure (IOP)[Ø] | X | | **Arm A** | **Arm A** | X | X | X | X | X | X | X |
| Lens grading[#] | **X** | | | | | | **X** | **X** | | **X** | **X** |
| VFQ-25, EQ-5D-5L and SWEMWBS questionnaires[¥] | **X** | | | | | | | **X** | | | **X** |
| Service user questionnaire | | | | | **X** | **X** | **X** | **X** | **X** | **X** | **X** |
| Fluorescein angiography (sent to reading centre)<br><br>[completed once only-see footnote]* | **Can be delayed until SMH clears sufficiently*** | | | | * | * | * | * | * | * | * |

| | | | | | | | | | | | |
|---|---|---|---|---|---|---|---|---|---|---|---|
| ICG angiography (sent to reading centre)<br><br>[completed once only-see footnote]* | **Can be delayed until SMH clears sufficiently*** | | | | * | * | * | * | * | * | * |
| OCT (sent to reading centre)[!] | **X** | | | | | | | | | | **X** |
| Clinic OCT (not sent to reading centre)[!] | | | | | X | X | X | X | X | X | |
| Stereo fundus photography (sent to reading centre)* | **X*** | | | | | | | | | | **X** |
| Autofluorescence (sent to reading centre)* | **X*** | | | | | | | | | | **X** |
| Adverse events (safety) | **X** | **X** | **Arm A** | **Arm A** | **X** | **X** | **X** | **X** | **X** | **X** | **X** |
| Concomitant medications | **X** | **X** | **Arm A** | **Arm A** | **X** | **X** | **X** | **X** | **X** | **X** | **X** |

Items in **red** occur primarily due to participation in TIGER, others are likely to occur regardless of trial participation but data will nonetheless be collected on the trial paper case report forms (CRFs) and electronic case report forms (eCRFs). Surgery (Arm A) may or may not be standard of care depending on the eye unit. D1=day 1; W1=week 1; M1= calendar month 1.

Participants withdrawing early should be invited, but not pressured, to attend an optional Exit Visit mirroring month 12 data collection.

**As blood is rapidly toxic to photoreceptors excessive delay may mean surgery is less effective, and the surgical risks may start to outweigh the potential benefits. Therefore, **it is very important that both screening and surgery are expedited**. Ideally, screening is completed in 1 day and surgery scheduled within 3 days of confirmed eligibility. Screening and surgery can occur on the same day, to avoid delay. In cases where the SMH onset is known, it should not have been present for more than 15 days at the point eligibility is confirmed. The maximum time between known SMH onset and surgery is 18 days, for example screening on days 13 and 14 after SMH onset, and surgery 4 days after that. If SMH onset is unknown, then the total time between the start of screening and surgery should be no more than 7 days (if the clinical features suggest SMH has been present >15 days patients are ineligible, even if this cannot be confirmed by history or pre-trial documentation). These allowances should not be used to delay surgery, which remains urgent in all cases.

**$** Full refracted ETDRS VA is undertaken in both eyes separately at baseline and month 12 and study eye only at month 6. Details in the protocol.

† Clinic ETDRS VA should be undertaken in the study eye only using an ETDRS chart with correction of any refractive error, with and without pinhole. Details in the protocol.

‡ Radner reading vision should be measured in the study eye only at screening, month 6, and month 12. Details in the protocol.

¢ Visual field tests should be completed in study eye only, scanned, and sent to the Reading Centre, as detailed in the protocol..

**Ø** Slit lamp examination and IOP should be undertaken in the both eyes at screening, month 6 and month 12, and study eye only at other visits.

# Lens grading is in both eyes at screening and month 12, but study eye only at months 4, 6 and 10. Details in the protocol.

¥ The VFQ-25 questionnaire includes the ‘optional’ questions listed in the protocol. The EuroQol questionnaire includes the 5-item vision bolt-on (EQ-5D-5L). SWEMWBS=Short Warwick-Edinburgh Mental Well-being Scale.

§ The initial intravitreal aflibercept injection can be administered on the day of screening, after eligibility is confirmed, in those randomised to the non-surgical group. In those randomised to surgery (Arm A), aflibercept is injected towards the end of surgery, straight after fluid-air exchange.

! At screening and month 12 ‘per protocol’ OCT images should be obtained in both eyes using certified staff and equipment, as per the Reading Centre’s instructions. These images are sent to the Reading Centre. At other visits a ‘Clinic OCT’ should be acquired in the study eye only, using standard staff and methodology. ‘Clinic OCT’ images are not sent to the reading centre. The attending clinical investigator should review all OCTs to monitor progress, watch for any emergent adverse events, and measure subretinal haemorrhage height. The same OCT machine should be used on a given participant throughout the study.

*Fluorescein and ICG angiography should be acquired in both eyes, once only. Either or both should be delayed if needed, to allow the SMH to clear sufficiently to enable visualisation of choroidal neovascularisation and/or polyps. The screening stereo fundus photography and fundus autofluorescence (FAF), undertaken in both eyes, should be repeated with delayed angiography, to help interpretation by the Reading Centre. Clinical investigators should review imaging to detect any emergent adverse events

## Reference List

1. Stanescu-Segall D, Balta F, Jackson TL. Submacular hemorrhage in neovascular age-related macular degeneration: A synthesis of the literature. *Surv Ophthalmol*. 2016;61(1):18-32. doi:10.1016/j.survophthal.2015.04.004

2. González-López JJ, McGowan G, Chapman E, Yorston D. Vitrectomy with subretinal tissue plasminogen activator and ranibizumab for submacular haemorrhages secondary to age-related macular degeneration: retrospective case series of 45 consecutive cases. *Eye (Lond)*. 2016;30(7):929-935. doi:10.1038/EYE.2016.65

3. Kunavisarut P, Thithuan T, Patikulsila D, et al. Submacular Hemorrhage: Visual Outcomes and Prognostic Factors. *Asia-Pacific J Ophthalmol (Philadelphia, Pa)*. 2018;7(2):109-113. doi:10.22608/APO.2017389

4. Carpenter J, Kenward M. *Missing Data in Randomised Controlled Trials: A Practical Guide*. Health Technology Assessment Methodology Programme; 2007. https://researchonline.lshtm.ac.uk/id/eprint/4018500/. Accessed November 15, 2021.

# Appendix A: Database variables

The main measures needed for the analyses are summarised below along with the corresponding variables—as named in MACRO database and the randomisation system—needed to define those measures.

| Measure | Derived from |
| --- | --- |
| **Primary outcome** | |
| Gaining ≥10 letters of BCVA in the study eye at the month 12 visit | REG_06, BCV_06, BCV_15 |
| **Secondary outcomes** | |
| Vision gain ≥10 ETDRS letters (at the 6 month visit). | REG_06, BCV_06, BCV_15 |
| Mean ETDRS BCVA (6 and 12 month). | REG_06, BCV_06, BCV_15 |
| Radner maximum reading speed (6 and 12 months). | REG_06, RAD_03, RAD_07 |
| Area of scotoma size using Humphrey Field Analyser 10-2 or equivalent (6 and 12 months). | VF_01, VF2_01, VF2_02, VF2_03, VF2_04, VF2_05, VF2_06, VF2_07 |
| NEI VFQ-25 composite score (6 and 12 months). | VFQ_01, VFQ_02, VFQ_03, VFQ_04, VFQ_05, VFQ_06, VFQ_07, VFQ_08, VFQ_09, VFQ_10, VFQ_11, VFQ_12, VFQ_13, VFQ_10, VFQ_11, VFQ_12, VFQ_13, VFQ_14, VFQ_15, VFQ_15a, VFQ_15b, VFQ_15c, VFQ_16, VFQ_16A, VFQ_17, VFQ_18, VFQ_19, VFQ_20, VFQ_21, VFQ_22, VFQ_23, VFQ_24, VFQ_25 |
| EQ-5D-5L with vision bolt-on score (6 and 12 months). | *To be identified by the health economist in the HEAP* |
| Presence or absence of subfoveal fibrosis and/or atrophy and area of fovea-involving fibrosis/atrophy assessed using multimodal imaging by an independent reading centre, combining spectral domain optical cMHerence tomography (SD-OCT), fundus autofluorescence (FAF) and stereo fundus photographs (month 12). | *To be identified by the Ophthalmic Image Reading Centres* |
| **Minimisation factors** | |
| Study site | Site Number |
| Lens status (phakic/ pseudophakic) | L0Item Value |
| SMH size (fully within the retinal vascular arcades, or not) | L1Item Value |
| BCVA (≥35 letters or not) | L2Item Value |
| **Demography** | |
| Age | REG_02 |
| Sex | REG_05 |
| Race | DEM_01, DEM_02 |
| **Ophthalmic history** | |
| Procedures/diagnoses | OH_01, OH_02, OH_03, OH_04, OH_05, OH_06 |
| Wet AMD | HIS_04 |
| Dry AMD | HIS_09 |

| | |
|---|---|
| **Medical history** | |
| Smoking status | DEM_03 |
| Hypertension | MH_02, MH_03 |
| Diabetes type 1 | MH_02, MH_03 |
| Diabetes type 2 | MH_02, MH_03 |
| Dyslipidaemia | MH_02, MH_03 |
| Stroke (cerebrovascular events) | MH_02, MH_03 |
| Transient ischaemic attack | MH_02, MH_03 |
| Atrial fibrillation | MH_02, MH_03 |
| Other | MH_02, MH_03 |
| **Drug history** | |
| Antiplatelets | HIS_15 |
| Anticoagulants | HIS_16 |
| Intravitreal Anti-VEGF injections | HIS_07, HIS_08 |
| **Adverse events** | |
| Severity | AE_8 |
| Relationship to surgery | AE_9 |
| Relationship to SF6 gas | AE_10 |
| Relationship to TPA | AE_11 |
| Relationship to aflibercept | AE_12 |
| Relationship to procedure of intravitreal injection | AE_13 |
| SAEs | AE_14 |
| Specific adverse events | |
| Cataract | AE_04 |
| Elevated IOP (>25 mmHg) | REG_06, IOP_01, IOP_02, AE_04 |
| Hypotony (IOP <5 mmHg) | REG_06, IOP_01, IOP_02, AE_04 |
| Retinal tear/detachment | AE_04 |
| Vitreous haemorrhage | AE_01, AE_02, AE_04 |
| Other | AE_01, |

# Appendix B: Compliance rules

| **Assessment Categorisation**<br><br>Red, Amber, Green. | **Visits** | | | | | | |
|---|---|---|---|---|---|---|---|
| | **M1** ± 7 days | **M2** ± 7 days | **M4** ± 7 days | **M6** ± 7 days | **M8** ± 7 days | **M10** ± 7 days | **M12** ± 7 days |
| Primary Outcome (Full Refracted ETDRS BCVA)<br><br>- Missing Month 12 Full Refracted BCVA (No, 777, 888, or 999)<br>- Month 12 Full Refracted BCVA more than 21 days outside of ±7 day visit window, as defined by visit planner or from last visit<br>- Missed aflibercept (Eylea) injection from M1 to M10<br><br>- Missing Full Refracted BCVA at Month 6<br>- Missing Clinic ETDRS BCVA from Months 1 to 10<br>- Missing slit lamp examination from Month 1 to 10<br>- Missing OCT from Month 1 to 10 (including both those sent to reading centre and those read on site)<br><br>- Per protocol | | | | | | | x |
| Visit Attendances<br><br>- Missed visit<br>- Visit more than 7 days outside of ±7 day visit window for Month 10, as defined by visit planner or from last visit<br><br>- Visit more than 21 days outside of ±7 day visit window for Month 1 to 8, as defined by visit planner or from last visit<br><br>- Per protocol | x | x | x | x | x | x | x |
