# Supplementary material for: Vitrectomy, subretinal Tissue plasminogen activator and Intravitreal Gas for submacular haemorrhage secondary to Exudative Age-Related macular degeneration (TIGER): update to study protocol and addition of a statistical analysis plan and health economic analysis plan for a randomised controlled surgical trial
Source: Trials. 2025 Apr 14;26:131. doi: 10.1186/s13063-025-08727-8 (PMC11995560; doi:10.1186/s13063-025-08727-8)
Supplement: Supplementary file 6 — Additional file 6: Appendix 6. TIGER health economic analysis plan. [file 13063_2025_8727_MOESM6_ESM.pdf]

A health economics bolt-on analysis of the Vitrectomy, subretinal Tissue plasminogen activator and Intravitreal Gas for submacular haemorrhage secondary to Exudative age-Related macular degeneration (TIGER) study

HEALTH ECONOMICS ANALYSIS PLAN (HEAP)

Version: V0.5

Date: 27/11/2023

Trial registration number: IRAS 276366

Trial funder: Fight for Sight and European Society of Retinal Specialists (EURETINA)

Trial Sponsor: King's College London and King's College Hospital NHS Foundation Trust

Health Economics analysis funder: Macular Society

Health Economics Sponsor: Bangor University

Clinical trial.gov identifier: NCT04663750; EudraCT: 2020-004917-10

Chief Investigator:

Professor Tim Jackson

King's College London, Department of Ophthalmology, King's College Hospital NHS Foundation Trust

Clinical Co-investigators:

Professor David Steel

Newcastle University

Professor Noemi Lois

Queen's University Belfast

Reading Centre Lead:

Professor Tunde Peto

Network of Ophthalmic Reading Centres UK Central Administrative Research Facility

Queen's University Belfast

Trial Methodologist:

Professor Barnaby Reeves

University of Bristol

Lead Statistician:

Dr Yanzhong Wang

King's College London

HEAP AUTHORS

The trial health economists Dr Victory Ezeofor, Miss Kodchawan (Pim) Doungsong, and Professor Rhiannon Tudor Edwards are responsible for operational conduct and reporting of the economic evaluation in accordance with the HEAP. Professor Edwards will oversee and contribute to the analysis and write up of the health economics analysis and act as guarantor of the economic evaluation.

Dr Victory Ezeofor

Signature and date

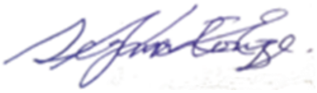

08/08/23

Miss Kodchawan Doungsong

Signature and date:

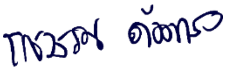

28/07/2023

Professor Rhiannon Tudor Edwards

Signature and date:

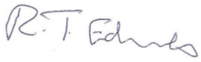

27/07/23

Lead health economist and guarantor of the economic evaluation

Professor Rhiannon Tudor Edwards

Signature and date:

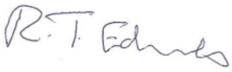

27/07/23

Chief Investigator

Prof Tim Jackson

Signature and date:

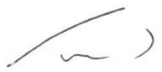

17/11/23

Table of Contents

1 Amendment history 7

2 Abbreviations, acronyms and definitions of terms 8

3 Trial overview 10

3.1 Trial background and rationale 10

3.2 Trial aim 10

3.3 Trial objectives 10

3.4 Trial design 11

Figure 1. TIGER Flow diagram 11

4 Economic analysis approach 12

4.1 Key dates 12

A GANTT chart outlining the tasks and key dates for the economic evaluation is shown in Appendix 1.12

4.2 Aims of the economic evaluation 12

4.3 Objectives of the economic evaluation 12

4.4 Economic evaluation design 12

Figure 2. Economic evaluation logic model of the TIGER trial 13

4.5 Perspective 14

4.6 Time horizon and discount rates 14

4.7 Data entry and management 14

4.8 Data validation and cleaning 14

4.9 Statistical software 14

4.10 Costing the intervention 14

4.11 Measurement and valuation of resource use data 14

4.12 Measurement and valuation of outcome data 14

EQ-5D-5L with vision bolt-on 14

National Eye Institute Visual Functioning Questionnaire-25 (VFQ-25) 15

Short Form Warwick Edinburgh Mental Wellbeing Scale (SWEMWBS) 15

4.13 Population for the base-case economic analysis 15

5 Cost-effectiveness analysis 16

5.1 Missing data 16

5.2 Analysis of costs 16

5.3 Analysis of outcomes 16

5.4 Analysis of cost-effectiveness 16

5.5 Handling uncertainty 16

5.6 Sensitivity analysis 17

6 Economic modelling 18

6.1 Modelling 18
6.2 Model structure 18
7 Wider cost consequence analysis 20
8 Budget impact analysis 20
9 Reporting checklists/standards 20
10 References 21
Appendix 1: GANTT chart for economic evaluation (v.1 18/11/22) 23
Appendix 2: Items recorded for surgery costing 24
Appendix 3: TIGER service user questionnaire 25

1 Amendment history

HEAP Version No.	Date issued	Author(s) of changes	Details of changes made
0.1	18/11/22	VE, RTE, JD	Draft HEAP started 18/11/2022
0.2	01/02/23	KD, VE	Added ‘What we already know’ section, copy of questionnaires, and updated health economic analysis
0.3	27/07/23	RTE	Update of team members and references and minor additions, added logic model
0.4	16/10/23	KD, VE	Revised HEAP, removed ‘What we already know’ section, and updated references
0.5	27/11/2023	KD, VE	Removed copied of questionnaires, checklists, and revised HEAP

2 Abbreviations, acronyms and definitions of terms

Acronym	Meaning
ADL	Activities of daily living
AMD	Age-related macular degeneration
BCVA	Best-corrected visual activity
CEAC	Cost-effectiveness acceptability curve
CHEERS	Consolidated Health Economic Evaluation Reporting Standards
CHEME	Centre for Health Economics and Medicines Evaluation, Bangor University
CRF	Case report form
CSRI	Client Service Receipt Inventory
EU	European Union
ETDRS	Early Treatment Diabetic Retinopathy Study
GDPR	General Data Protection Regulation
HEAP	Health Economics Analysis Plan
HRQOL	Health-related quality of life
ICER	Incremental cost-effectiveness ratio
ISPOR	International Society for Pharmacoeconomics and Outcomes Research
ITT	Intention to treat
KCTU	King’s Clinical Trial Unit
NEI	National Eye Institute (USA)
NHS	National Health Service
QALY	Quality-Adjusted Life Year
RCT	Randomised controlled trial

SAP	Statistical Analysis Plan
SMH	Submacular haemorrhage
TPA	Tissue plasminogen activator
VAS	Visual analogue scale
VEGF	Vascular endothelial growth factor
VFQ-25	25-item Visual Function Questionnaire
SWEMWEBS	Short Warwick Edinburgh Mental Wellbeing Scale

3 Trial overview

Section 3 is adapted from the TIGER study protocol version 3.0 dated 29th March 2023.[1]

3.1 Trial background and rationale

Wet age-related macular degeneration (AMD) is one of the commonest causes of blindness in developed nations. It is usually treated with repeated injections of anti-vascular endothelial growth factor (anti-VEGF) drugs into the eye. A small proportion of people with wet AMD develop a large clot in the back of their eye, called a 'submacular haemorrhage' (SMH). Untreated, SMH usually causes permanent, severe vision loss.

The ongoing TIGER study is a pan-European randomised-controlled surgical trial including up to 10 countries (United Kingdom, Bulgaria, France, Germany, Ireland, Italy, Netherlands, Poland, Spain, and Switzerland). This study evaluates surgery called vitrectomy to deliver a 'clot busting' drug (tissue plasminogen activator, TPA) underneath the retina to dissolve SMH, combined with a gas bubble in the eye to push the dissolved clot away from the macula, and a course of regular injections of aflibercept anti-VEGF; compared to a course of aflibercept injections alone.

TIGER already collects health economics data about the resources used by participants and their quality of life as they progress through the trial, but the original funding cap was not sufficient to fund a health economics analysis. Funding of a health economics bolt-on study was provided by the Macular Society.

Rationale

The need to present evidence for the relative cost-effectiveness of vitrectomy, TPA, gas, plus aflibercept compared to aflibercept alone in the treatment of SMH is twofold. Firstly, it is not clear whether surgery followed by aflibercept injections will improve vision more than aflibercept injections alone, and whether the costs and potential complications of surgery will offset any visual benefit. Secondly, TPA is not licensed for ocular use (although it is commonly used in an off-license capacity to treat SMH in routine practice) and anti-VEGF agents (including aflibercept) have strict visual acuity criteria to qualify for National Health Service (NHS)-funded treatment, which patients with SMH due to AMD are often unable to meet at presentation due to their sight being too poor.

If surgery and aflibercept is superior to aflibercept alone, this may expand patient's access to surgery, with potential implications on licensing of TPA for ocular use. If surgery and aflibercept is not superior to aflibercept alone, patients may be spared the risk, discomfort and inconvenience of surgery, and healthcare providers may be spared the expense of surgery. In either situation, a health economic analysis will be highly valuable to health planners and commissioners to weigh up the relative benefits and costs of surgery vs no surgery, and also the value of offering licensed anti-VEGF in patients with SMH due to AMD who would otherwise not qualify for NHS-funded treatment.

3.2 Trial aim

To assess the safety and efficacy of vitrectomy, subretinal TPA, gas tamponade and intravitreal anti-VEGF as a treatment for SMH secondary to exudative AMD, versus standard of care with anti-VEGF monotherapy.

3.3 Trial objectives

Primary outcome

The primary outcome is the proportion of participants with a BCVA gain ≥10 Early Treatment Diabetic Retinopathy Study (ETDRS) letters in the study eye at the 12-month follow-up visit.

Secondary outcomes

- Vision gain ≥10 ETDRS letters (at the 6-month visit)

- Mean ETDRS BCVA (6 and 12 months)
- Radner maximum reading speed (6 and 12 months)
- Area of central scotoma size using Humphrey Field Analyser 10-2 or equivalent (6 and 12 months)
- National Eye Institute VFQ-25 composite score (6 and 12 months)
- Presence or absence of subfoveal fibrosis and/or atrophy and area of fovea-involving fibrosis/atrophy using multimodal reading centre image analysis (month 12).

3.4 Trial design

TIGER is a phase 3, multicentre, pan-European, non-commercial, randomised, two-group, active control, superiority, observer-masked, surgical trial. It is summarized in Figure 1.

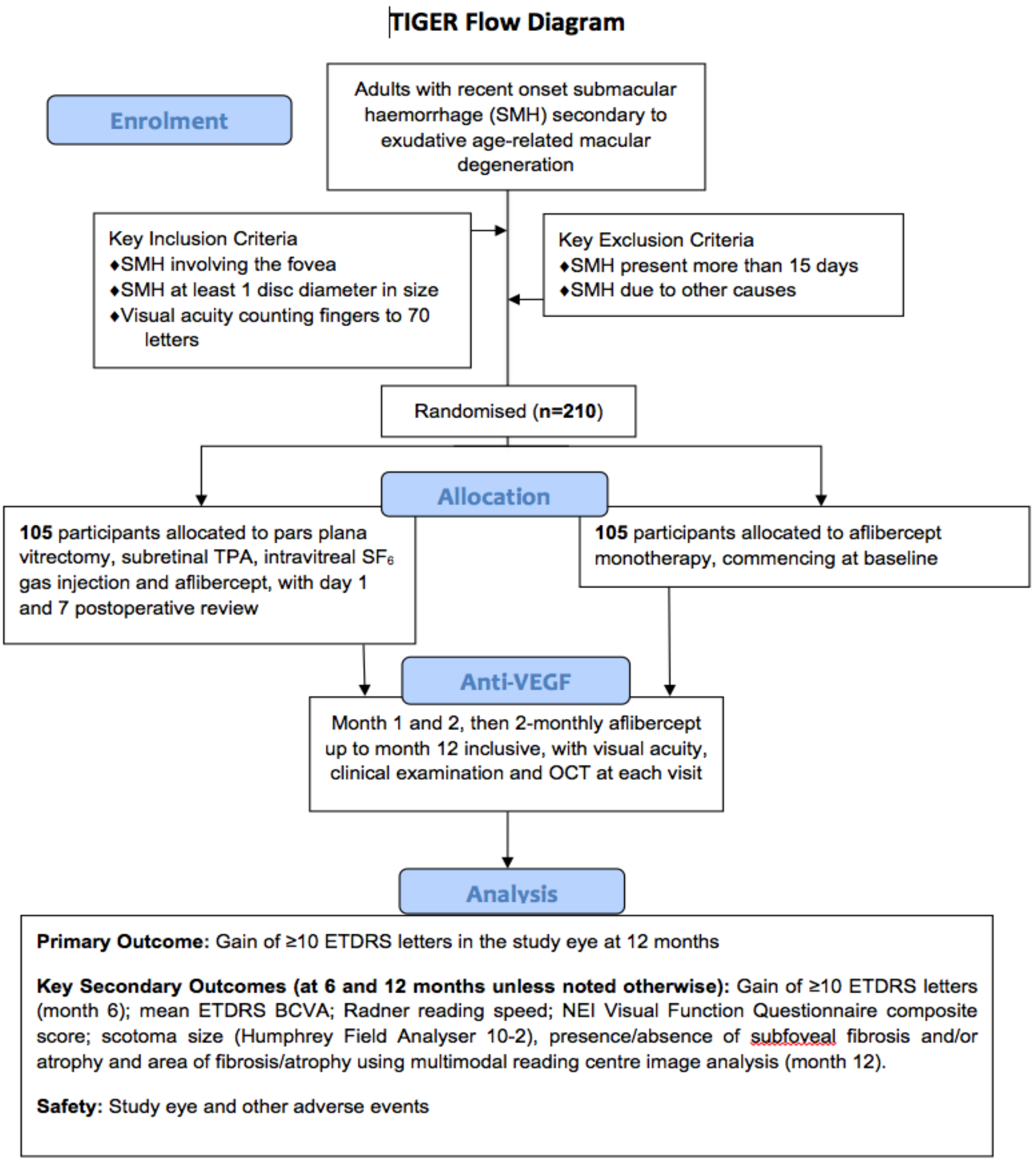

Figure 1. TIGER Flow diagram

4 Economic analysis approach

4.1 Key dates

Recruitment for the main trial began in April 2021 and is projected to run for a period of at least 38 months.

In the full trial, participants will complete economic outcome measures (NEI-VFQ-25, SWEMWBS, and EQ-5D-5L) at baseline, 6 months and 12 months.

A GANTT chart outlining the tasks and key dates for the economic evaluation is shown in Appendix 1.

4.2 Aims of the economic evaluation

The aims of the TIGER study health economics bolt-on are to address the following research questions:

- How cost-effective is vitrectomy, subretinal TPA, intravitreal gas, plus aflibercept, as compared with aflibercept monotherapy, at achieving a 10 letter gain in BCVA in the treatment of SMH secondary to wet AMD?
- What is the budget impact to the NHS of rolling out vitrectomy, TPA, intravitreal gas and aflibercept for SMH secondary to wet AMD, if found to be cost-effective?
- How do the ways we measure visual acuity, well-being and health-related quality of life in patients with SMH interplay with each other?

4.3 Objectives of the economic evaluation

Although this is a pan-European study, this health economic evaluation will be conducted using data from the UK sites only. The template and data will enable similar analyses in other participating countries involved in the trial, subject to funding and relevant approvals.

The primary objective of the economic evaluation is to estimate the cost-effectiveness of vitrectomy, subretinal TPA, intravitreal gas, plus aflibercept for SMH secondary to wet AMD, as compared with usual care (in this case, aflibercept monotherapy).

The secondary objectives are to estimate the impact on the NHS budget of rolling out vitrectomy, TPA, intravitreal gas, plus aflibercept for SMH secondary to wet AMD, if it is found to be cost-effective. The other secondary objective is to assess the different outcome measurements of this trial: visual acuity, well-being and health-related quality of life. This will be achieved through comparisons of respondent outcome measures across the different instruments to identify any potential trends or correlation between them. This analysis will take the form of a subsidiary paper to the economic evaluation.

4.4 Economic evaluation design

The economic evaluation will take the form of cost-effectiveness and cost-utility analyses using individual level participant data from the trial[2,3] and existing evidence identified from a rapid review of cost-effectiveness literature in this area. The cost-utility analysis will use the patient reported outcome from the quality-of-life questionnaire (EQ-5D-5L with vision bolt-on)[4,5] to generate the cost of each Quality Adjusted Life Year (QALY) gained by participants across both trial arms.

If SMH surgery is found to be cost-effective we will estimate the financial impact to the NHS of offering SMH (‘budget impact analysis’), based on the resources used.

The logic model below (Figure 2) summarises our approach to economic evaluation.

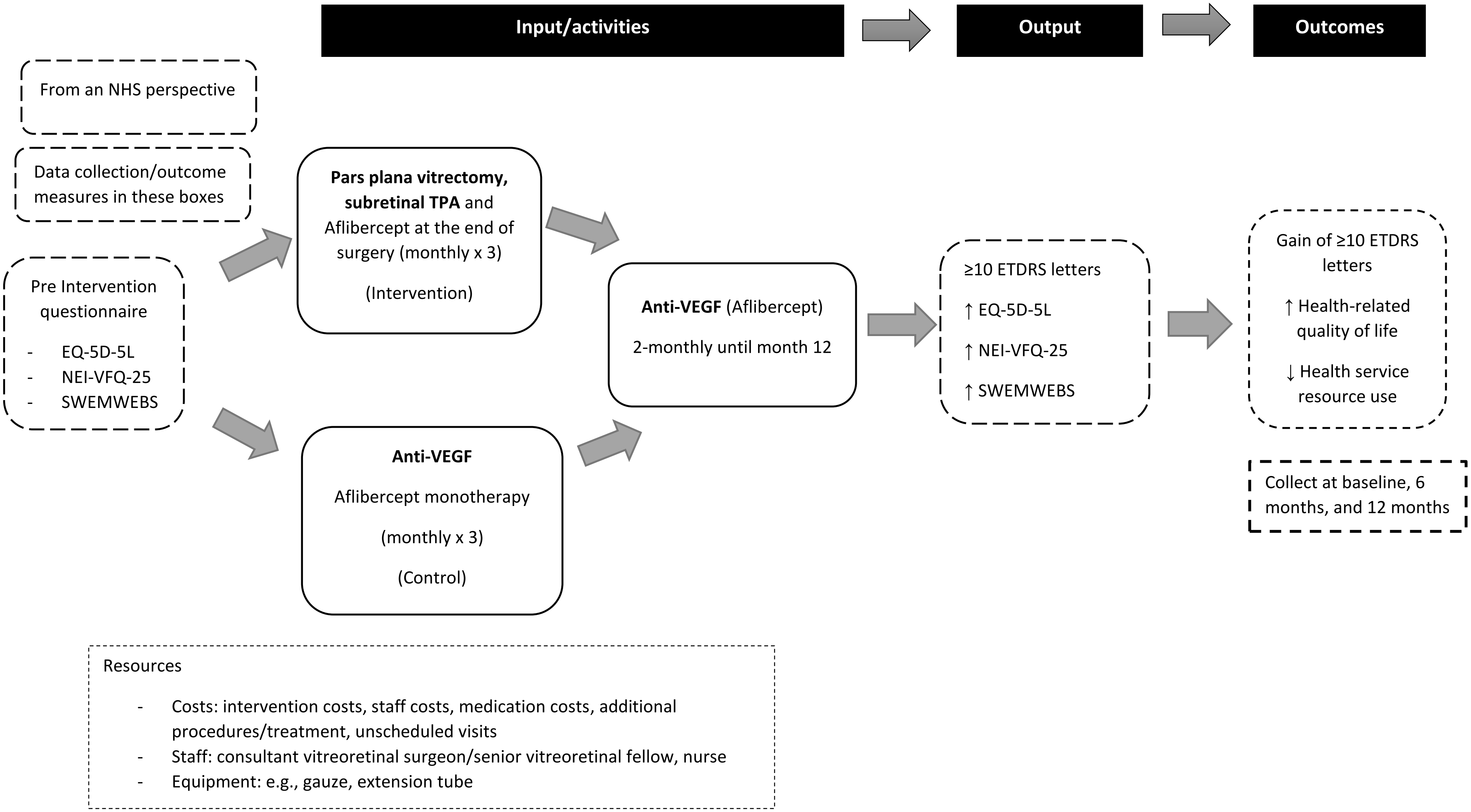

Figure 2. Economic evaluation logic model of the TIGER trial

4.5 Perspective

This health economics analysis will be conducted using data that were obtained from the UK site only (data will be made available to enable similar analyses in other European countries, subject to funding and relevant approvals). The cost-effectiveness analysis will be undertaken from an NHS perspective and personal social services (PSS) perspective.

4.6 Time horizon and discount rates

The cost-effectiveness analysis will take a life-time horizon in accordance with NICE guidance.[6] Costs and benefits will be discounted at 3.5% for analysis beyond 12 months.[6]

4.7 Data entry and management

Trial data will be entered on electronic case report forms (eCRFs) as pseudo-anonymised or fully anonymised format, in accordance with local legal and ethics committee regulations, by each participating site's research teams. The eCRF data capture system for TIGER is Elsevier MACRO, managed by the King's Clinical Trials Unit (KCTU), who will also provide extracts of the trial data to the health economics team on request. All received data will be protected by password conforming to the Bangor University's data security policy with data compliance and European Union (EU) General Data Protection Regulation (GDPR) policies.

4.8 Data validation and cleaning

Face validity checks will be conducted on the data to identify typos and numerical outliers. Discrepancies will be checked against the source documents and a log of errors kept. The health economists will notify KCTU of any errors identified.

4.9 Statistical software

This analysis will be undertaken in STATA version 13.0 and Microsoft Office Excel 365 which will be used to carry out the cost-effectiveness analyses.

4.10 Costing the intervention

Data on the intervention costs will be collected during the trial; this will include surgery costs, equipment/instrument costs, medication costs, and staff costs. A sample list of items used to record intervention costs is shown in Appendix 2.

4.11 Measurement and valuation of resource use data

Resource use data will be collected as part of the eCRF at each data collection point; see Appendix 3. Resource use will be costed using national unit costs.[7,8] For resource use data, a baseline equivalent test will be conducted, and base case analysis of the trial data will be investigated.

4.12 Measurement and valuation of outcome data

All outcomes will be measured at baseline, 6 months, and 12 months.

EQ-5D-5L with vision bolt-on

The EQ-5D-5L is a Quality-of-Life measurement instrument that asks respondents to rate their health on a given day across five dimensions: Mobility, Self-care, Usual activities, Pain / discomfort, Anxiety / depression.[4,9]

For this study, a vision bolt-question on[10] will be added to the EQ-5D-5L questionnaire to assess the quality-of-life impacts of respondents' vision throughout the trial across both arms.

National Eye Institute Visual Functioning Questionnaire-25 (VFQ-25)

The VFQ-25 is a 25-item, ophthalmology specific questionnaire which was developed to measure the impact of vision impairment on physical and social functioning, and emotional well-being.[11] Bressler et al. (2009) found 10-point difference in NEI VFQ-25 scores is clinically important and correlates with a 15-letter change in visual acuity.[12] It was also suggested that a 5-point change is also clinically important.[12]

Short Form Warwick Edinburgh Mental Wellbeing Scale (SWEMWBS)

The 7-item short form Warwick-Edinburgh Mental Wellbeing Scales has 5 response categories, summed to provide a single score that requires transforming from a provided table. The items cover both feeling and functioning aspects of mental wellbeing of a respondents' last two weeks up to questionnaire administration.[13]

4.13 Population for the base-case economic analysis

The trial will recruit 210 participants randomised 1:1 to surgery or standard care arms.

5 Cost-effectiveness analysis

5.1 Missing data

Missing data will be handled in accordance with the Statistical Analysis Plan (SAP).

In the case of the EQ-5D-5L with vision bolt-on, we will follow guidance on how to handle missing items/ data points.[14]

5.2 Analysis of costs

Differences in overall mean costs between the arms will be presented.

5.3 Analysis of outcomes

The primary outcome for the cost-effectiveness analysis will be quality-adjusted life years (QALYs) at 12 months. Utility values will be obtained from responses to the EQ-5D-5L with vision bolt-on at baseline, 6 months and 12 months for the primary analysis. A scoring algorithm using UK tariff values will be used to convert responses into an index score of between -0.594 and 1, with 1 representing full HRQoL.

Other secondary outcomes will be investigated and compared with the primary outcome results. These secondary outcomes will include generic and disease specific measures such as the VFQ-25 and the ETDRS visual acuity score, and the well-being measure, SWEMWBS.

5.4 Analysis of cost-effectiveness

Cost and QALY data will be combined to calculate an incremental cost-effectiveness ratio (ICER) using a life-time horizon:

$$\mathrm{ICER} = \frac{(\mathrm{C1} - \mathrm{C0})}{(E1 - E0)}$$

C1= cost in intervention group
C0= cost in control group
E1= effect in intervention group
E0= effect in control group

The data derived from the clinical trial intervention will be used to compare outcomes such as cost, benefits and consequences.

5.5 Handling uncertainty

The nonparametric bootstrapping approach will be used to determine the level of sampling uncertainty surrounding the mean ICER by generating 5,000 estimates of incremental costs and benefits.[15] A cost-effectiveness plane such as the one below will be plotted to illustrate where the distribution of the estimates fall.

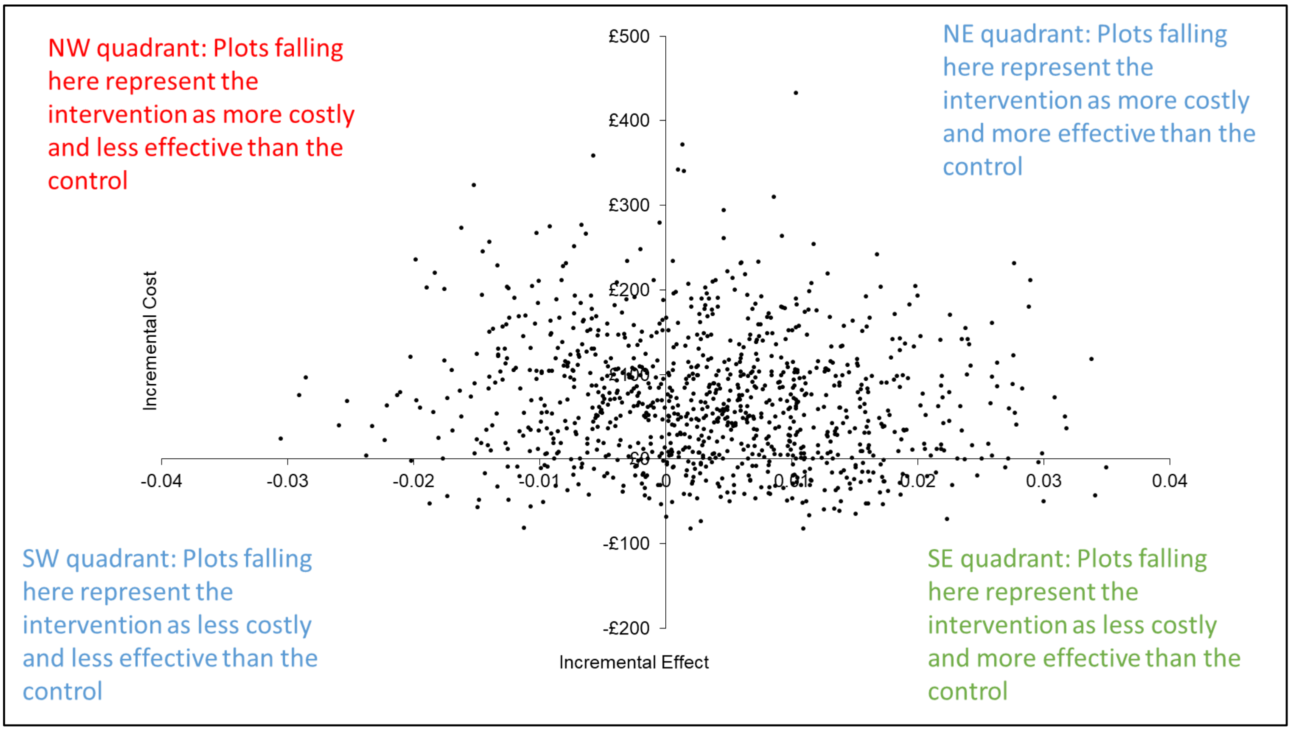

A cost-effectiveness acceptability curve (CEAC) will be plotted to represent the probability of the intervention at any given willingness-to-pay threshold.[16] For example, in the CEAC below, the probability that the intervention is cost-effective at a threshold of £30,000 per QALY gained is 55%.

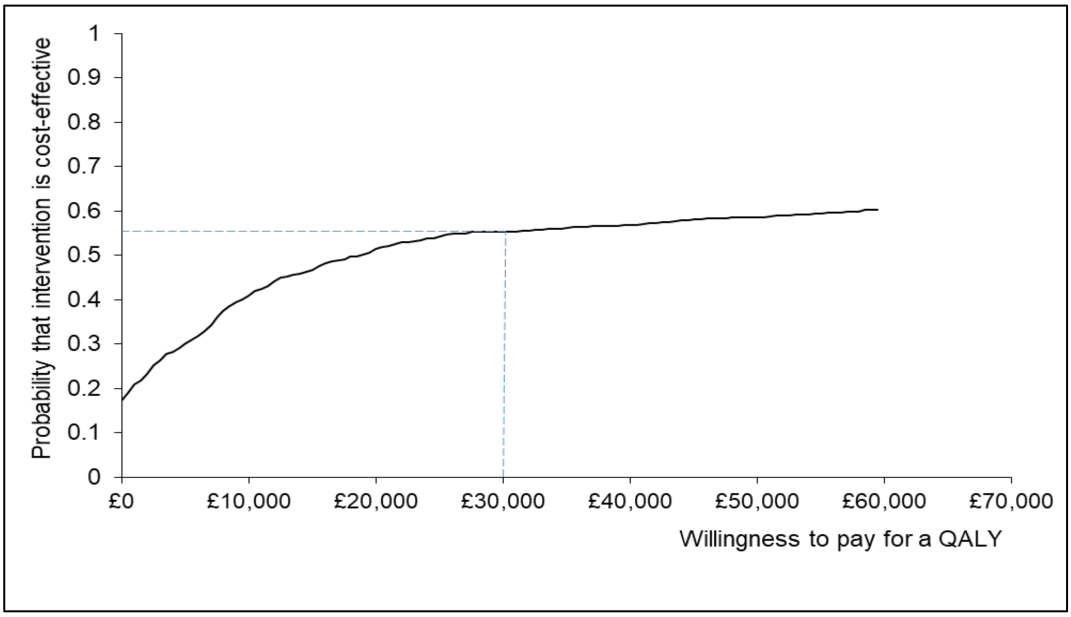

5.6 Sensitivity analysis

There is the possibility of variability and heterogeneity affecting the outcome of the cost-effectiveness analysis. To address the issues of variability arising from randomness in data[17] we will follow standard practice by running the model repeatedly to obtain a stable estimate.

Heterogeneity will be investigated for different subgroups to avoid unrealistic differences in cost-effectiveness that invalidates or diminishes the robustness of outcomes from the study.[18] To achieve this, we will identify and select the subgroups appropriately.

The results for complete cost and quality of life data (i.e., those with no missing data) as well as a strict per-protocol analysis of the data will be provided to identify the impact of missing data on the analysis and any sensitivity to protocol violations. Deterministic and probabilistic sensitivity analysis will be undertaken. Sensitivity analyses will also be conducted to vary the costs of inputs (e.g., the associated resource cost of surgery for SMH), and to vary the discount rate applied (base case rate of 3.5%, sensitivity analysis rates of 0% and 1.5%) in accordance with NICE guidelines.[6,19]

6 Economic modelling

6.1 Modelling

The synthesis and design of this study will be conducted using the decision analytical modelling technique[20] from which the Markov Monte Carlo simulation model will be structured.[21]

6.2 Model structure

The Markov model (figure 3) will simulate patients' transit through defined Markov states in time represented by cycles. During this, patients can remain in their current state, move to another health state or reach the absorbed state, in this case loss of sight. The movement of patients from one state to another is according to certain transition probabilities which will be captured by patient data collected at baseline and the follow-up period.[22] The Markov states are finite and based on health or QoL properties suited for the intervention and illness. The patients in our economic model will be classed under two groups; intervention and control group. This model will be used to extrapolate evidence for a lifetime horizon.[23]

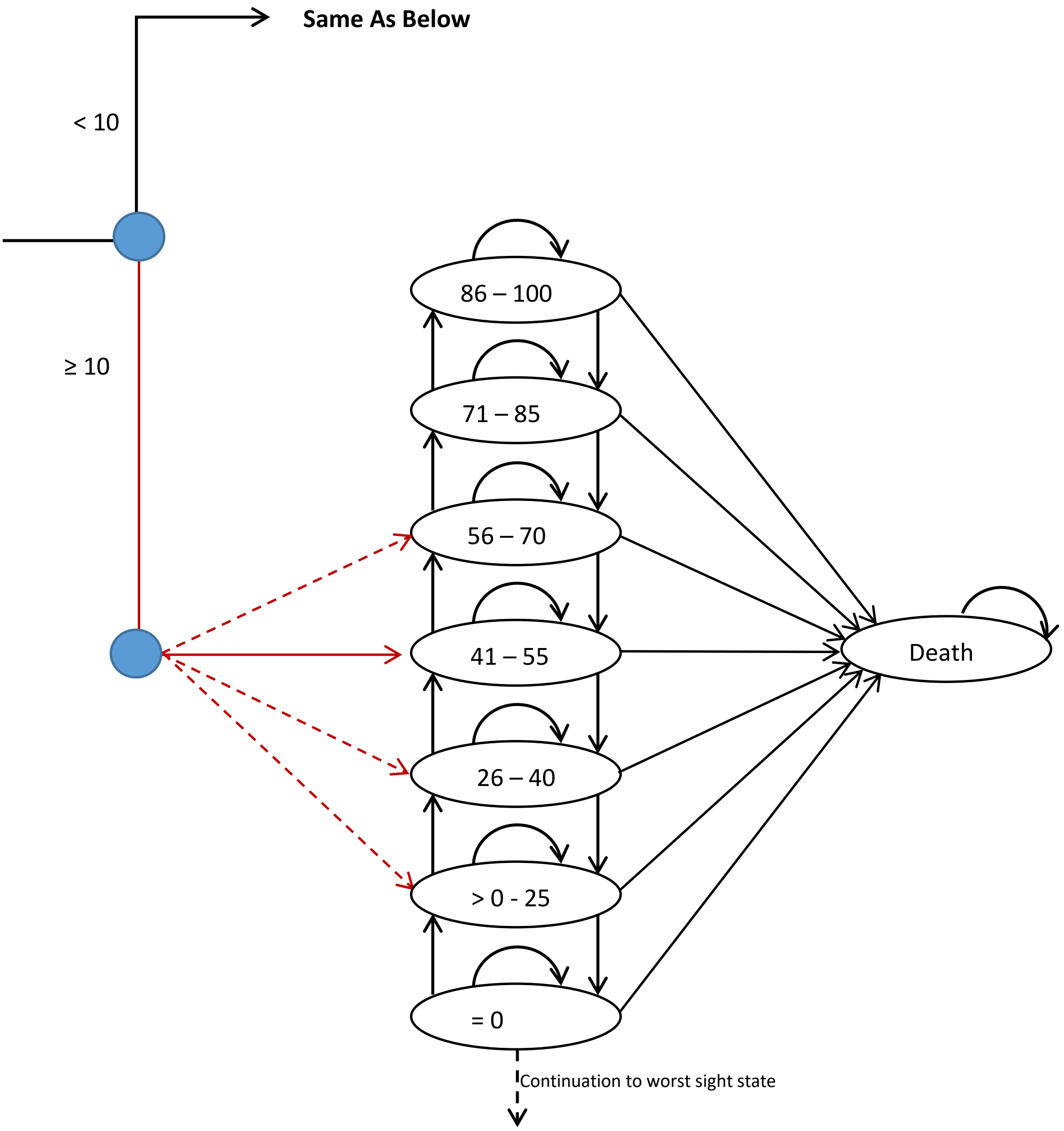

Figure 3. Schematic diagram of the Markov model representing the clinical progression of submacular haemorrhage. Scores obtained from the EDTRS scale. Red broken line are possibilities. The arrows show the transition from one health state to another or the same health state in the next cycle.

The Markov model will consist of a two-month cycle length for the analysis. The model contains eight different health states with arrows indicating possible transitions among them. Best corrected visual activity (BCVA) is typically used to generate health state in ophthalmology.[24] There will be six health states classified according to levels of BCVA, ranging from ≤25 ETDRS letters to 100. Total loss of sight is represented with the least BCVA score, see figure 3. After these patients received the treatments, they would proceed to the states based on the above classification of 10 letters with the possibility of total loss of sight or death.

The model would compare the cost of the intervention to the benefit derived by evaluating the cost-effectiveness of this study. The decision analytical modelling technique provides decision and policy makers

with the best evidence to reach a decision about a healthcare intervention.[25] Though there are other techniques that could be applied to this study, the benefits of applying the decision analytical modelling are far greater.[26]

7 Wider cost consequence analysis

In order to reflect the fact that changes in visual acuity measurement does not capture the full impact of changes in vision on a person, we are calculating a cost per QALY and also including a cost-consequence analysis which sets out costs of the intervention relevant to a range of outcomes i.e. change in vision-related quality of life measured by the VFQ-25 and changes in patient-reported well-being using the SWEMWBS.

8 Budget impact analysis

An economic budget impact model will be developed to determine the budget implications and any expected cost savings as a direct result of the NHS offering surgery to clear SMH.

We will be guided by the International Society for Pharmacoeconomics and Outcomes Research (ISPOR) good practice recommendations usually used with the introduction of new pharmaceutical products; these guidelines are equally useful for the development of a budget impact model for the use of surgery to treat SMH.[27] We will model the budget impact. We will undertake sensitivity analyses, varying relevant assumptions (i.e., alternative scenarios). We will validate the model in terms of face validity with ophthalmic surgeons. We will populate the model with data from the TIGER trial and the literature. We will use a budget impact cost calculator approach.

The budget impact analysis will lead to potentially improved resource allocation by the NHS as it takes a payer's perspective analysis.

9 Reporting checklists/standards

The Consolidated Health Economic Evaluation Reporting Standards (CHEERS)[28] and the Assessment of the Validation Status of Health-Economic decision models (AdVisHE)[29] will be followed when reporting the health economic evaluation. These checklists will ensure that bias is minimised.

10 References

1. Jackson TL, Bunce C, Desai R, et al. Vitrectomy, subretinal Tissue plasminogen activator and Intravitreal Gas for submacular haemorrhage secondary to Exudative Age-Related macular degeneration (TIGER): study protocol for a phase 3, pan-European, two-group, non-commercial, active-control, observer-masked, superiority, randomised controlled surgical trial. *Trials* 2022; 23: 99.

2. Drummond M, Claxton M, Stoddart G, et al. *Methods for the Economic Evaluation of Health Care Programmes Fourth Edition*. Oxford University Press, 2015.

3. Glick HA, Doshi J, Sonnad S, et al. Economic Evaluation in Clinical Trials, 2nd Edition. *Aust N Z J Public Health*; 39. Epub ahead of print 2015. DOI: 10.1111/1753-6405.12438.

4. EuroQol Research Foundation. EQ-5D-5L User Guide 2019, https://euroqol.org/publications/user-guides. (2019, accessed 24 May 2023).

5. De Sousa Peixoto R, Krstic L, Hill SCL, et al. Predicting quality of life in AMD patients—insights on the new NICE classification and on a bolt-on vision dimension for the EQ-5D. *Eye* 2021; 35: 3333–3341.

6. National Institute for Health and Care Excellence (NICE). *Guide to the processes of technology appraisal*, https://www.nice.org.uk/Media/Default/About/what-we-do/NICE-guidance/NICE-technology-appraisals/technology-appraisal-processes-guide-apr-2018.pdf (April 2018, accessed 28 July 2023).

7. Jones K, Burns A. *Unit Costs of Health and Social Care 2021*. Canterbury, https://www.pssru.ac.uk/project-pages/unit-costs/unit-costs-of-health-and-social-care-2021/ (2021, accessed 30 March 2023).

8. National Health Services (NHS). National Cost Collection: National schedule of NHS costs - Year 2021/22, https://www.england.nhs.uk/publication/2021-22-national-cost-collection-data-publication/ (2023, accessed 28 July 2023).

9. Herdman M, Gudex C, Lloyd A, et al. Development and preliminary testing of the new five-level version of EQ-5D (EQ-5D-5L). *Quality of Life Research*; 20. Epub ahead of print 2011. DOI: 10.1007/s11136-011-9903-x.

10. de Sousa Peixoto R, Krstic L, Hill SCL, et al. Predicting quality of life in AMD patients—insights on the new NICE classification and on a bolt-on vision dimension for the EQ-5D. *Eye (Basingstoke)*; 35. Epub ahead of print 2021. DOI: 10.1038/s41433-021-01414-3.

11. Mangione CM, Lee PP, Gutierrez PR, et al. Development of the 25-list-item National Eye Institute Visual Function Questionnaire. *Archives of Ophthalmology* 2001; 119: 1050–1058.

12. Bressler NM, Chang TS, Fine JT, et al. Improved Vision-Related Function After Ranibizumab vs Photodynamic Therapy: A Randomized Clinical Trial. *Archives of Ophthalmology* 2009; 127: 13–21.

13. Stewart-Brown S, Tennant A, Tennant R, et al. Internal construct validity of the Warwick-Edinburgh Mental Well-being Scale (WEMWBS): a Rasch analysis using data from the Scottish Health Education Population Survey. *Health Qual Life Outcomes* 2009; 7: 15.

14. Simons CL, Rivero-Arias O, Yu L-M, et al. Multiple imputation to deal with missing EQ-5D-3L data: Should we impute individual domains or the actual index? *Quality of Life Research* 2015; 24: 805–815.

15. Briggs AH, Gray AM. Methods in health service research: Handling uncertainty in economic evaluations of healthcare interventions. *BMJ : British Medical Journal* 1999; 319: 635.

16. Fenwick E, O'Brien BJ, Briggs A. Cost-effectiveness acceptability curves – facts, fallacies and frequently asked questions. *Health Econ* 2004; 13: 405–415.

17. Briggs A, Sculpher M, Buxton M. Uncertainty in the economic evaluation of health care technologies: The role of sensitivity analysis. *Health Econ* 1994; 3: 95–104.

18. Bago d'Uva T, Van Doorslaer E, Lindeboom M, et al. Does reporting heterogeneity bias the measurement of health disparities? *Health Econ* 2008; 17: 351–375.

19. Andronis L, Barton P, Bryan S. Sensitivity analysis in economic evaluation: an audit of NICE current practice and a review of its use and value in decision-making. 2009; 13: 29.

20. Karnon J. Alternative decision modelling techniques for the evaluation of health care technologies: Markov processes versus discrete event simulation. *Health Econ* 2003; 12: 837–848.

21. Sonnenberg FA, Beck JR. Markov Models in Medical Decision Making. *http://dx.doi.org/101177/0272989X9301300409* 1993; 13: 322–338.

22. Siebert U. When should decision-analytic modeling be used in the economic evaluation of health care? *European Journal of Health Economics* 2003; 4: 143–150.

23. Buxton MJ, Drummond MF, van Hout BA, et al. Modelling in economic evaluation: an unavoidable fact of life. *Health Econ* 1997; 6: 217–227.

24. Claxton L, Hodgson R, Taylor M, et al. Simulation Modelling in Ophthalmology: Application to Cost Effectiveness of Ranibizumab and Aflibercept for the Treatment of Wet Age-Related Macular Degeneration in the United Kingdom. *Pharmacoeconomics* 2017; 35: 237–248.

25. Petrou S, Gray A. Economic evaluation using decision analytical modelling: design, conduct, analysis, and reporting. *BMJ*; 342. Epub ahead of print 11 April 2011. DOI: 10.1136/BMJ.D1766.

26. Sun X, Faunce T. Decision-Analytical Modelling in Health-Care Economic Evaluations. *The European Journal of Health Economics* 2008; 9: 313–323.

27. Mauskopf JA, Sullivan SD, Annemans L, et al. Principles of Good Practice for Budget Impact Analysis: Report of the ISPOR Task Force on Good Research Practices-Budget Impact Analysis. 2007; 1098.

28. Husereau D, Drummond M, Augustovski F, et al. Consolidated Health Economic Evaluation Reporting Standards 2022 (CHEERS 2022) Statement: Updated Reporting Guidance for Health Economic Evaluations. *BMJ* 2022; 376: 067975.

29. Vemer P, Corro Ramos I, van Voorn GAK, et al. AdViSHE: A Validation-Assessment Tool of Health-Economic Models for Decision Makers and Model Users. *Pharmacoeconomics*; 34. Epub ahead of print 2016. DOI: 10.1007/s40273-015-0327-2.

Appendix 1: GANTT chart for economic evaluation (v.1 18/11/22)

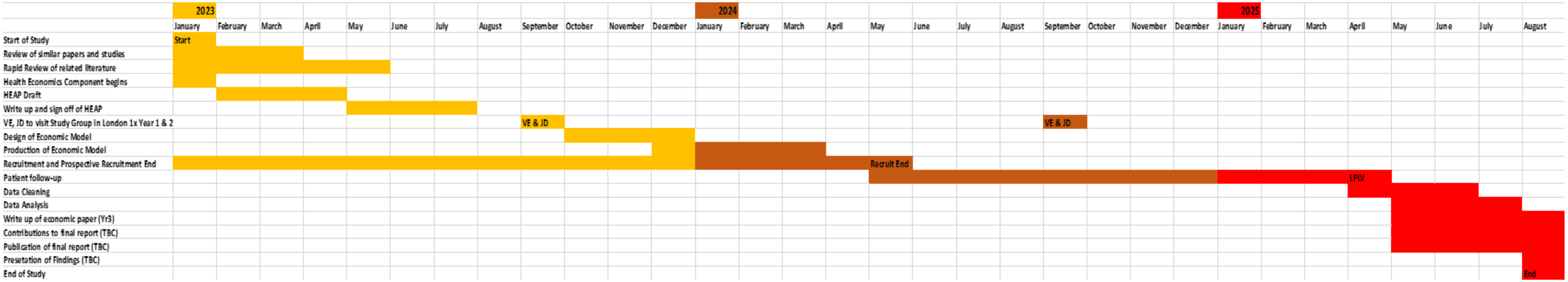

Appendix 2: Items recorded for surgery costing

Note that research costs are not collected e.g. training time spent instructing staff on questionnaires, time spent arranging interviews with participants, time spent completing questionnaires

**Item**	**Unit (cost/time)**	**Number of units**
Non-recurrent initial training and set up costs		
Materials e.g. equipment, handouts, DVDs given to staff or participants. Please do not include the cost of research materials		
Staff training for surgery<br>a. Time at training for staff<br>b. Time spent travelling to training sessions<br>c. Mileage to/from training<br>d. Staff grade attending training<br>e. Was backfill required? I.e. were replacement staff put on the rota to cover training related absences?<br>f. Venue costs<br>g. Refreshment costs<br>h. Ongoing supervision (i.e. senior staff coaching/ providing guidance to intervention staff)		
Surgery costs<br>a. In-theatre costs<br>a. Staff costs: calculate from using a length of surgery (hourly rate); 1-2 surgeons (consultant and fellow); 1 anaesthetist (consultant or associated), 1 anaesthetist, 2 circulating nurses, 1 scrub nurse<br>b. Consumables<br>c. Equipment<br>b. Out-of-theatre costs<br>a. Staff costs: ophthalmologists, anaesthetist, pre-/post-operative nursing<br>b. Overnight stays<br>c. Emergency visits related surgery<br>d. Identifiable out-of-surgery costs: eye drops<br>e. Other costs: pharmacists (fixed rate)		
Overheads (a fixed % will be added, to be agreed between Bangor/Nottingham)	N/A	N/A

Appendix 3: TIGER service user questionnaire

TIGER Service User Questionnaire

Please add the following questions to the TIGER CRF at ALL data collection points.

This section asks about the health care services that you have used over the past month (baseline) and throughout the study at each data point since the last assessment. It also asks about the medications that you use.

1. Hospital Service Use

Interviewer instructions: Please complete the table to show the hospital services that the participant has used at baseline or since the last follow-up.

Service [used by participant]	Name of ward, clinic, hospital or centre	Reason for using service (e.g. nature of illness, regular respite arrangement)	Unit of measurement	Total number of units received	Number of online/phone appointments
Assessment/rehabilitation inpatient ward			Inpatient day		
Medical/surgical inpatient ward			Inpatient day		
Ophthalmology inpatient ward			Inpatient day		
Other inpatient			Inpatient day		
Ophthalmology outpatient			Appointment		
Outpatient services			Appointment		
Accident and Emergency (A&E)			Attendance		

Day hospital			Day attendance		
Counselling service			Appointment		
Other (1)			Please specify:		
Other (2)			Please specify:		
Other (3)			Please specify:		

**Service** **[Used by participant]**	**Number of home visits**	**Number of visits to surgery or clinic**	**Number of online/phone appointments**	**Average duration of contact (minutes)**
General practitioner (GP)				
Practice nurse (GP clinic)				
Optician				

Example Current Hospital Service User Questionnaire (Case Report Forms Version 3.0; 13.04.2023):

TIGER	Vitrectomy and subretinal TPA for submacular haemorrhage secondary to wet AMD (TIGER)	PIN	P	Participant Initials			
		Study Eye	OD / OS	Date	DD	MM	YY

SERVICE USER QUESTIONNAIRE – HOSPITAL SERVICES

Record any face-to-face or virtual consultations, or admissions, ONLY if they relate to the STUDY eye. Please include those with secondary care such as eye clinics, and hospitals. For example, emergency/eye department attendance for post-operative raised intraocular pressure in the study eye, hospital admission due to a fall caused by low vision in the study eye, or virtual consultation with a psychologist for depression caused by worsening vision in the study eye.

Hospital services used since last questionnaire (if first questionnaire, include services received since randomisation)	If other hospital service, please specify	Reason for using service(s)	Total Inpatient days	Total face-to-face Outpatient / Day case / Emergency attendances	Total virtual appointments (e.g., phone or online)
☐ No hospital services to record at this timepoint<br>☐ Inpatient (Ophthalmology)<br>☐ Inpatient (Medicine/Surgery/Other)<br>☐ Outpatient (Ophthalmology)<br>☐ Outpatient (Medicine/Surgery/Other)<br>☐ Emergency department including Eye Emergency<br>☐ Day Hospital visit e.g. day case surgery<br>☐ Counselling service<br>☐ Other not included in above list			☐☐☐	Outpatient: ☐☐☐<br>Day Case: ☐☐☐<br>Emergency: ☐☐☐	☐☐☐

Investigator Signature: ____________________ **DATE:** ____/_____/_____

TIGER STUDY v3.0 13/04/23

Example Current Community Service User Questionnaire (Case Report Forms Version 3.0; 13.04.2023):

TIGER	PIN	P	Participant Initials				
	Study Eye	OD / OS	Date	DD	MM	YY	

SERVICE USER QUESTIONNAIRE – COMMUNITY SERVICES

Instructions: Record any face-to-face or virtual consultations ONLY if they relate to a STUDY eye condition including those with primary care such as family doctors. If this is the first Service User Questionnaire (i.e. Month 1), ensure community services used since randomisation are recorded. At subsequent visits, record community services used since the last Service User Questionnaire was completed.

General practitioner (GP) or family doctor used by participant? ☐ Yes ☐ No

Number of home visits ☐☐☐

Number of visits to surgery or clinic ☐☐☐

Number of online or telephone appointments ☐☐☐

Average duration of contact with doctor (minutes) ☐☐☐

Practice nurse (GP or family doctor clinic) used by participant? ☐ Yes ☐ No

Number of home visits ☐☐☐

Number of visits to surgery or clinic ☐☐☐

Number of online or telephone appointments ☐☐☐

Average duration of contact with doctor (minutes) ☐☐☐

TIGER STUDY v3.0 13/04/23

TIGER	PIN	P	Participant Initials				
	Study Eye	OD / OS	Date	DD	MM	YY	

Optician service used by participant? ☐ Yes ☐ No

Number of home visits ☐☐☐

Number of visits to optician office ☐☐☐

Number of online or telephone appointments ☐☐☐

Average duration of contact with optician (minutes) ☐☐☐

Investigator Signature: ____________________ **DATE:** ____/_____/_____

TIGER STUDY v3.0 13/04/23
